# Coupling with opinion dynamics promotes prosocial behavior in multilayer networks

**DOI:** 10.1371/journal.pcbi.1014810

**Published:** 2026-09-23

**Authors:** Jnanajyoti Bhaumik, Naoki Masuda

**Affiliations:** 1 Department of Mathematics, State University of New York at Buffalo, Buffalo, New York, United States of America; 2 Gilbert S. Omenn Department of Computational Medicine and Bioinformatics, University of Michigan, Ann Arbor, Michigan, United States of America; 3 Department of Mathematics, University of Michigan, Ann Arbor, Michigan, United States of America; 4 Center for Computational Social Science, Kobe University, Kobe, Japan; Interdisciplinary Transformation University IT:U, AUSTRIA

## Abstract

Various theoretical and empirical studies have accounted for why humans cooperate in competitive environments. Although prior work has revealed that network structure and multiplex interactions can promote cooperation, most theory assumes that individuals play dilemma games in all social contexts. However, real-world agents may participate in a diversity of interactions, not all of which present dilemmas. We develop an evolutionary game model on multilayer networks in which one layer supports the prisoner’s dilemma game, while the other follows constant-selection dynamics, representing biased but non-dilemmatic competition, akin to opinion or fad spreading. Our theoretical analysis reveals that coupling a social dilemma layer to a non-dilemmatic constant-selection layer robustly enhances cooperation in many cases, across different multilayer networks, updating rules, and payoff schemes. These findings suggest that embedding individuals within diverse networked settings—even those unrelated to direct social dilemmas—can be a principled approach to engineering cooperation in socio-ecological and organizational systems.

## Author summary

Why do people cooperate even when selfish behavior looks rewarding? Network models show that who interacts with whom matters, but most assume the same kind of dilemma in every social setting. We develop a theory for populations embedded in two overlapping interaction networks: one in which individuals face a prisoner’s dilemma game, and another in which two opinions spread under competition with a built-in bias but no dilemma. We show that linking these layers can systematically increase the chance that cooperation takes over, across network structures, update rules, and how public goods are distributed. The work suggests new, principled ways to foster cooperation in dilemmatic situations by shaping non-dilemmatic social contexts.

## 1 Introduction

Altruistic behavior is a key feature of both humans and non-human organisms. Over past decades, studies of social dilemma games, most famously represented by the prisoner’s dilemma game, have revealed various mechanisms with which individuals cooperate when prosocial behavior is desirable for the society but apparently irrational for individuals. Such mechanisms include kin selection, repeated interactions between the same individuals, reputations, and population structure [1–3].

Networks, or population structure in general, promote prosocial behavior in multiple ways. For example, clustering of individuals, quantified by the abundance of short cycles composed of a few individuals, promotes cooperation because short cycles create locally connected clusters of cooperators that protect themselves against exploitation by non-cooperators [4–6]. Any edge (i.e., dyad) forming a network in fact creates assortative connectivity between cooperators, promoting cooperation [7]. Heterogeneity in the degree (i.e., the number of neighbors that an individual has) across individuals also promotes cooperation under some assumptions [8–10]. Time-varying nature of networks can also promote cooperation [11–14], whereas the opposite holds true in some situations [15]. All these network features are shared by a vast majority of empirical social networks [16–18].

In fact, humans, as well as animals and organizations, can interact with others in multiple social contexts and modalities. For example, we may interact with each other both in person and online. This situation can be modeled by a multilayer network, of which each network layer is defined by one type of edge (i.e., interaction) [19–22]. Various numerical studies have shown that cooperation can be enhanced in multilayer networks [23–27]. Su et al. pioneered a theoretical framework to understand cooperation in multilayer networks [28]. They assumed that each player is involved in a distinct prisoner’s dilemma game with peers in each network layer and that the payoff of each player that guides evolutionary dynamics in the different layers is the sum of the payoffs that the player obtains across all the network layers. They showed that multilayer networks enhance cooperation under broad conditions, compared to each network layer separately considered.

Social dilemmas are not the only situation that humans are coping with. Even if we face a social dilemma in our daily life, we would also be simultaneously involved in other types of social but non-dilemmatic interactions. A game formulation of this situation is to make individuals play different types of games in different network layers [27,29–31]. Seminal studies also analyzed a related situation in which the social dilemma game and imitation of behavior occur in different network layers [32,33]. How robust is enhanced cooperation in multilayer networks when individuals are not always participating in social dilemma games? Although there are some numerical results along this direction [27], can we provide theoretical underpinnings to elucidate how non-dilemmatic social dynamics modify the likelihood of prosocial behavior in dilemmatic situations? To answer these questions, we propose an evolutionary game model on two-layer networks (which can be readily extended to the case of more than two layers) in which players are involved in the prisoner’s dilemma game in one network layer and constant-selection dynamics in the other layer. The constant-selection dynamics is a simple social dynamics in which two types (e.g., opinions) compete in a population. It is equivalent to the biased voter model, a model of consensus formation dynamics used in mathematics [34,35] and interdisciplinary physics [36,37] for many years. By extending a recently developed theoretical framework [28], we show that the constant-selection dynamics can promote cooperation played in the opposite network layer in many cases. This study expands the set of scenarios that promote cooperation in multilayer populations and furnishes a theoretical framework to study agents’ multifaceted payoff-seeking behavior when social-dilemma interactions account for only part of their payoffs.

## 2 Model

Fig 1 is a schematic of our evolutionary dynamics model on two-layer networks. We assume that there are *N* individuals. The replica node refers to a node of the undirected network layer. Therefore, there are 2*N* replica nodes, and each individual is represented by two replica nodes. Each edge directly connects two replica nodes in the same layer and represents one type of pairwise interaction between individuals. We assume that the individuals play the donation game, a special case of the prisoner’s dilemma game, with each of their neighbors in layer 1 and that the evolutionary dynamics are governed by constant selection in layer 2.

**Fig 1:**
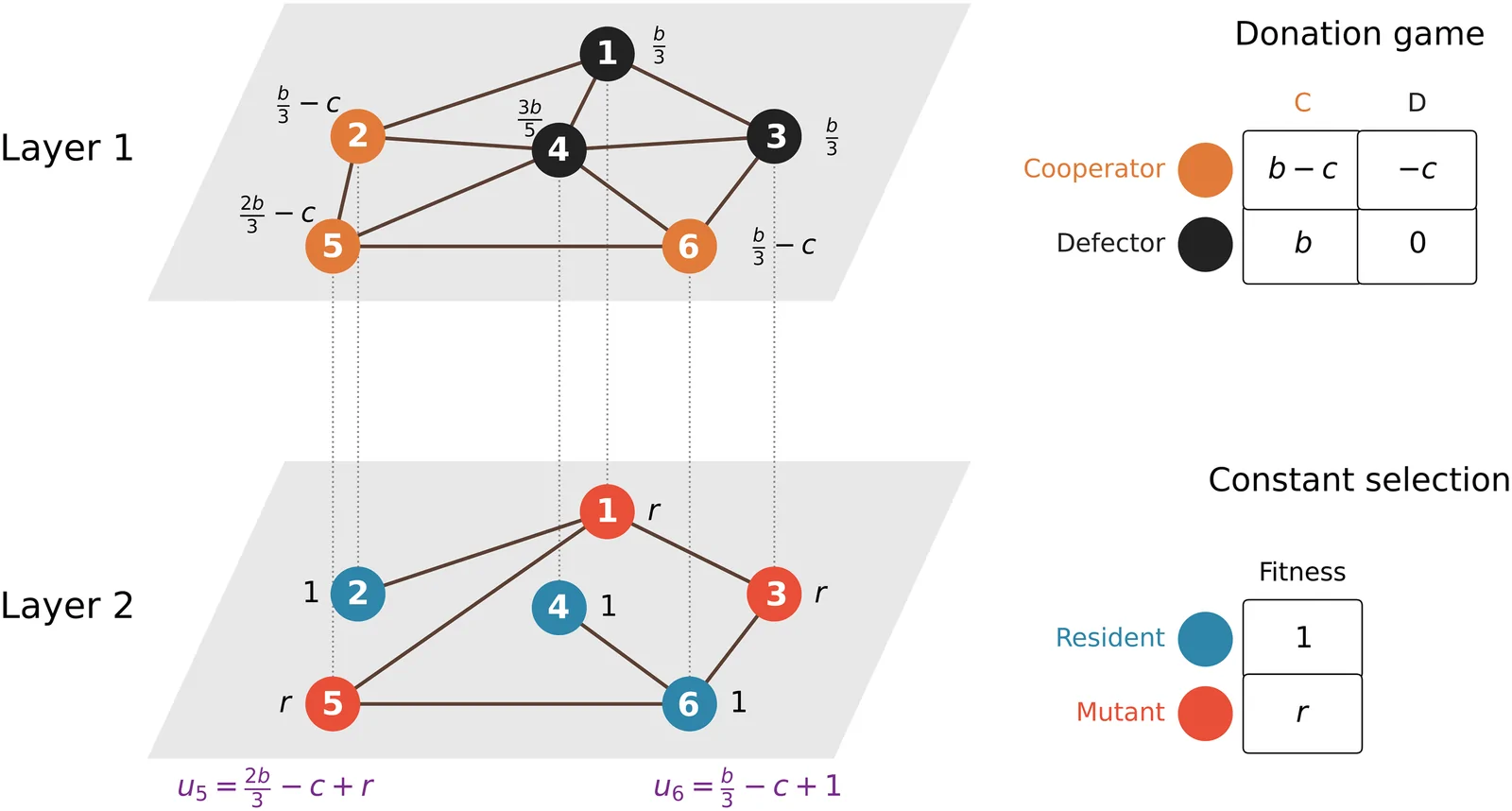
Schematic of the two-layer game. The donation game is played in layer 1. The constant-selection dynamics occurs in layer 2. The total payoff for each individual, which drives evolutionary dynamics in both layers, is the sum of the payoffs that the individual obtains from both layers. The figure shows the total payoffs for individuals 5 and 6 as examples. In both layers, replica nodes (shown by circles) that have a high total payoff tend to spread its type to its neighbors.

In layer 1, each *i*th individual is either a cooperator or a defector, encoded into $x_{i}^{\left[1\right]}=1$ and $x_{i}^{\left[1\right]}=0$, respectively. Each cooperator pays a cost *c* and provides a benefit *b* to each of its neighbors; this is called a pf goods scheme [38]; we will examine a different goods scheme later. The weight of an edge from the *i*th replica node to the *j*th replica node within layer L∈{1,2} is denoted by $w_{ij}^{\left[L\right]}$. We denote the weighted degree (which we simply call the degree in the following text) of the *i*th replica node in layer *L* by $s_{i}^{\left[L\right]}:=\sum_{j=1}^{N}w_{ij}^{\left[L\right]}$. The payoff of the *i*th replica node in layer 1 is given by [28,39]


$$\begin{array}{c} u_{i}^{\left[1\right]}=-cx_{i}^{\left[1\right]}+\sum_{j=1}^{N}bp_{ij}x_{j}^{\left[1\right]}, \end{array}$$ (1)

where $p_{ij}=w_{ij}^{\left[1\right]}/s_{i}^{\left[1\right]}$. This form of $p_{ij}$ models a variant of the “pf goods” scheme in [38], which dictates that the cooperator pays the cost *c* per neighbor and each of its neighbor receives a benefit *b*. We divide the total payoff that each individual receives by the (weighted) degree of the node, $s_{i}^{\left[1\right]}$, for normalization. We examine a different payoff scheme in the section “Fixed benefits, fixed costs” goods scheme.

In layer 2, each *i*th individual is assumed to be either the mutant or resident type, encoded into $x_{i}^{\left[2\right]}=1$ and $x_{i}^{\left[2\right]}=0$, respectively. The fitness of the mutant and resident type is denoted by *r* and 1, respectively. Therefore, the payoff of the *i*th replica node in layer 2 is given by


$$\begin{array}{c} u_{i}^{\left[2\right]}=x_{i}^{\left[2\right]}\left(r-1\right)+1. \end{array}$$ (2)

The total payoff of the *i*th individual, denoted by $u_{i}$, is assumed to be the sum of payoff across both layers, i.e., $u_{i}=u_{i}^{\left[1\right]}+u_{i}^{\left[2\right]}$.

We assume that the evolutionary dynamics is governed by the death-Birth (dB) updating rule. (Capitalization of B indicates that selection occurs at birth [40]; also see [41–43] for earlier studies that distinguish death-birth processes depending on whether selection occurs at death or birth.) At the beginning of every time step in the evolutionary dynamics, we choose a replica node *i* in layer 1 uniformly at random (with probability 1/*N*) to update its strategy. Then, the neighbors of *i* in layer 1 compete to disseminate its strategy to *i*. A neighbor of *i* is chosen with the probability proportional to their fecundity, where fecundity of an individual *i* is defined as $F_{i}=1+\delta u_{i}$; note that δ (≥0) is the strength of selection. The type of *j* (i.e., cooperation or defection) replaces that of *i* in layer 1. Simultaneously, in layer 2, in a similar fashion, an individual *j* is selected for death uniformly at random. Next, a neighbor of *j* in layer 2 is selected for reproduction with the probability proportional to $u_{j}$. Then, the type of *j* (i.e., mutant or resident) replaces that of *i* in layer 2, concluding one time step of the evolutionary dynamics. This process is repeated until there are either only cooperators or only defectors in layer 1 and there are either only mutants or only residents in layer 2. Throughout the text, we assume weak selection, that is, δ≪1. With the form of $F_{i}$ and reproduction rule described above, we are assuming that the selection strength is the same between the two layers.

We use the dB updating rule for layer 1 because it is often used for studying prosociality on networks [7,28,44–47]. We use the same updating rule in layer 2 for simplicity. We call the combined updating rule the dB-dB rule. In fact, a different updating rule called the Birth-death (Bd; selection occurs at birth) is more commonly used for constant-selection evolutionary dynamics [40,48]. Therefore, we also investigate the Bd rule for layer 2. In this case, we call the combined updating rule the dB-Bd rule.

## 3 Results

### 3.1 Conditions under which cooperators and mutants are favored

We derived the condition under which the cooperator is favored in layer 1 and that under which the mutant is favored in layer 2 for arbitrary two-layer networks (see Text A in S1 Text). More specifically, we derived the fixation probability for a single cooperator introduced to the population of defectors in layer 1 under weak selection, and similar for a single mutant introduced in layer 2. The derivation expands that in [28], where individuals are assumed to play the donation game in both networks. A key observation that we have exploited in our derivation is that constant-selection dynamics can be expressed as evolutionary game dynamics where the payoff matrix entries do not depend on the opponent’s action. Nonetheless, the constant selection produces terms that are not present in the case of the two-layer donation game. After obtaining a general solution (i.e., last equation in Text A in S1 Text), we substitute the quantities for a given updating rule to obtain the conditions for the dB-dB and dB-Bd rules (see Texts B and C in S1 Text, respectively).

Under the dB-dB rule, we have found that the condition under which cooperation is favored in layer 1 is given by


$$\begin{array}{c} c\theta_{2}+b\left(\theta_{1}-\theta_{3}\right)-\left(r-1\right)\phi_{2,0}>0. \end{array}$$ (3)

Here, $\theta_{n}$ (denoted by $\theta_{n}^{\xi \left[1\right]}$ in Text B in S1 Text) is the equilibrium probability that the initial and final nodes of a *n*-step random walk occurring within layer 1 have the same strategy, with a minor modification. It is calculated from a combination of the solution of a set of *O*(*N*^2^) linear equations, the *n*-step transition probability matrix of the random walk on the layer-1 network, and its stationary probability at each replica node. Quantity $\phi_{n,m}$ (denoted by $\phi_{n,m}^{\xi \left[1,2\right]}$ in Text B in S1 Text) is the equilibrium probability that the initial node of the random walk is in layer 1 and the final node in layer 2 have the same strategy, with a minor modification, conditioned that the first *n* steps of the random walk occur in layer 1 and the subsequent *m* steps occur in layer 2. Quantity $\phi_{n,m}$ is similarly obtained from a combination of the solution of a different set of *O*(*N*^2^) linear equations, the transition probability matrix of the aforementioned random walk with *n* + *m* steps, and the stationary probability of the layer-1 random walk at each replica node.

Under the same dB-dB rule, the condition under which the mutant is favored in layer 2 is given by


$$\begin{array}{c} -\left(r-1\right){\overset{―}{\theta }}_{2}+c\phi_{0,2}+b\left(\phi_{1,0}-\phi_{1,2}\right)>0, \end{array}$$ (4)

where ${\overset{―}{\theta }}_{2}$ is the same as $\theta_{2}$ except that the random walk occurs within layer 2 instead of layer 1 (denoted by $\theta_{n}^{\xi \left[2\right]}$ in Text B in S1 Text). Equations (3) and (4) are our main theoretical results. While they are outcomes of detailed mathematical analyses shown in Texts A and B in S1 Text, we provide more intuitive explanation of these inequalities in Text D in S1 Text. We derive that explanation by imposing that a cooperator or mutant neighbor of an individual that is being updated, denoted by *i*, has a higher average payoff than a random neighbor of *i*. A similar explanation was provided for the two-layer network model in which individuals play the donation game in both layers [28].

These theoretical results hold for weighted two-layer networks. In the following demonstration, however, we focus on unweighted networks for simplicity.

### 3.2 Classification of two-layer networks and exhaustive examination of networks with *N* = 6 individuals

#### 3.2.1 Evolution of cooperators.

Let us more closely examine the conditions given by Eqs. (3) and (4). When *r* = 1, Eq. (3) reduces to the condition for favoring cooperation in one-layer networks [28,39]. This is because setting *r* = 1 implies that the payoff from the constant-selection layer (i.e., layer 2) is equal to 1 for all individuals and therefore only makes the selection weaker for the donation game layer (i.e., layer 1).

To classify consequences of Eq. (3) depending on the parameter and variable values, we first assume that $\theta_{1}-\theta_{3}>0$ and rewrite Eq. (3) as follows:


$$\begin{array}{c} \frac{b}{c}>{\left(\frac{b}{c}\right)}^{*}\equiv \frac{-\theta_{2}}{\theta_{1}-\theta_{3}}+\frac{(r-1)\phi_{2,0}}{c\left(\theta_{1}-\theta_{3}\right)}. \end{array}$$ (5)

The network-dependent constant $(b/c{)}^{*}$ is the value of *b*/*c* above which cooperation is favored over defection in layer 1 of the two-layer network; however, we also discuss other interpretations of $(b/c{)}^{*}$ below. When *r* = 1, it is known that $(b/c{)}^{*}$ is positive because $\theta_{2}<0$ [13,28,39], and Eq. (5) gives the condition for cooperation. Relative to this one-layer baseline case first shown in [39], coupling with the constant-selection dynamics layer, i.e., r≠1, changes $(b/c{)}^{*}$ as follows. Note that (r−1)/c is the relative weight of the constant selection to the donation game. We distinguish between the following three cases (see Fig 2A).

**Fig 2:**
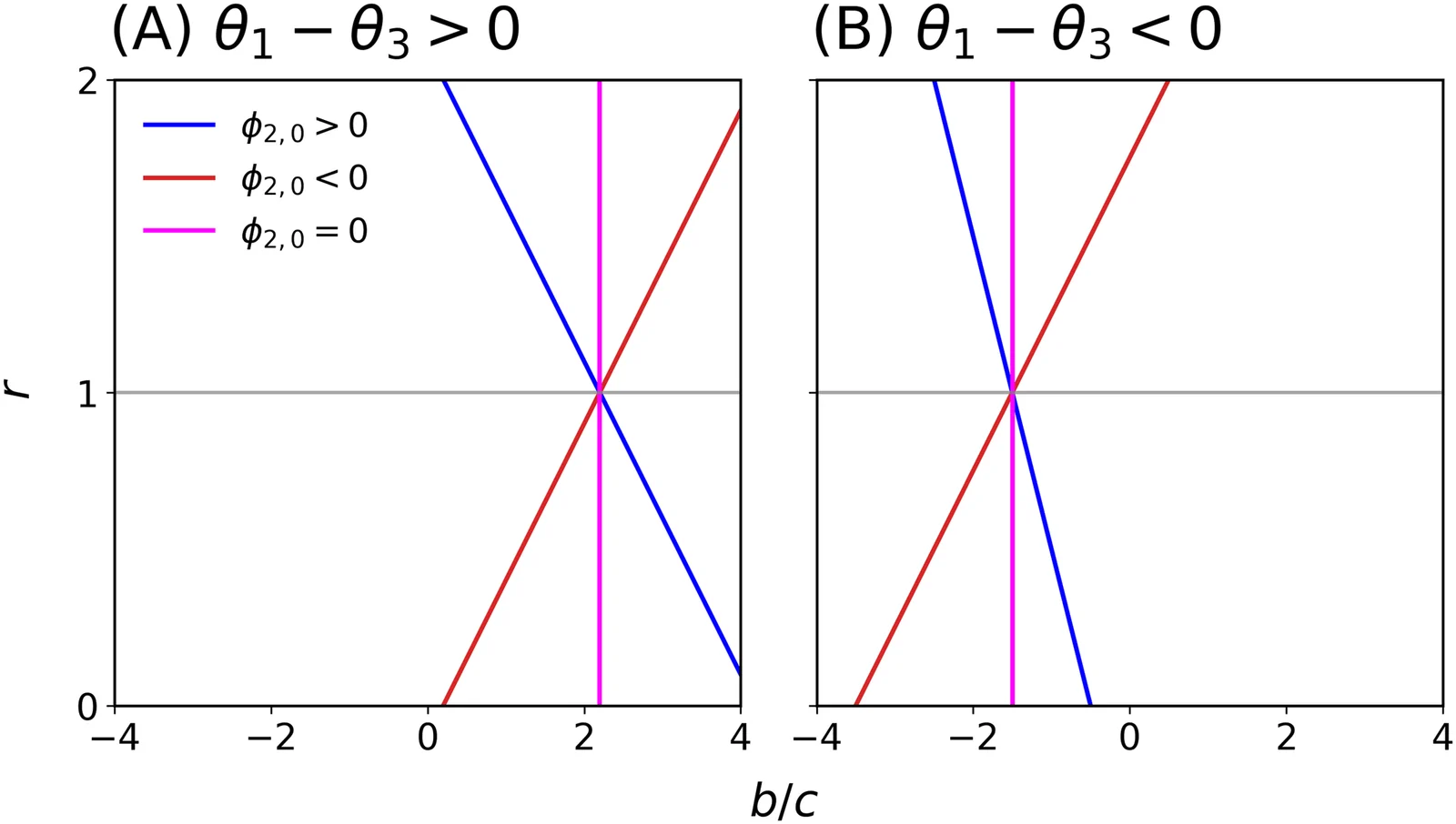
$(b/c{)}^{*}$ for various values of *r.* (A) $\theta_{1}-\theta_{3}>0$. (B) $\theta_{1}-\theta_{3}<0$. In (A), cooperation is favored when *b*/*c* is larger than the value specified by the line, whose slope depends on $\phi_{2,0}$. In (B), spite is favored when *b*/*c* is smaller than the value specified by the line depending on the $\phi_{2,0}$ value.

1. If $\phi_{2,0}>0$, then $(b/c{)}^{*}$ is lower in the two-layer than the one-layer network if 0≤r<1, with the two-layer network easing cooperation. In contrast, the two-layer network makes cooperation harder if *r* > 1.
2. If $\phi_{2,0}<0$, then $(b/c{)}^{*}$ is lower in the two-layer than the one-layer network if $1<r<\overset{―}{r}$, facilitating the cooperation. Constant $\overset{―}{r}$ (>1) is given below. The two-layer network makes cooperation harder if 0≤r<1.
3. If $\phi_{2,0}=0$, then $(b/c{)}^{*}$ does not depend on *r* such that $(b/c{)}^{*}$ remains the same as the case of *r* = 1. In this case, coupling two network layers does not change the condition for cooperation.

In case 1, if $\phi_{2,0}>-c\theta_{2}$ (> 0) and $0<r<\overset{―}{r}\equiv 1+\left(c\theta_{2}/\phi_{2,0}\right)$ (<1), then cooperation is favored when $b/c>(b/c{)}^{*}$ with a negative threshold $(b/c{)}^{*}$. It should be noted that, when the spite behavior (in place of cooperation) is favored over defection, the condition generally reads $b/c<(b/c{)}^{*}<0$; this is how we interpret the $b/c<(b/c{)}^{*}<0$ case in general [39]. In contrast, the inequality is in the opposite direction in the present case. When $b/c>(b/c{)}^{*}$ with a negative $(b/c{)}^{*}$, the interpretation of the result is difficult. Note that the same interpretability problem can occur when players are involved in different donation games in each network layer [28]. However, we confirmed in our numerical simulations described in the following text that there is no network yielding $b/c>(b/c{)}^{*}$ with a negative $(b/c{)}^{*}$. Similarly, in case 2, if $r>\overset{―}{r}$ (>1), cooperation is favored when $b/c>(b/c{)}^{*}$ with a negative $(b/c{)}^{*}$. However, we have numerically confirmed that two-layer networks can show $b/c>(b/c{)}^{*}$ with a negative $(b/c{)}^{*}$ only when *r* is much larger than 1, where the weak-selection assumption is compromised.

Second, when $\theta_{1}-\theta_{3}<0$, we obtain Eq. (5) but with the inequality being flipped. Then, the first term on the right-hand side of Eq. (5) is negative. Therefore, if *r* = 1 or $\phi_{2,0}=0$ (see Fig 2B), then the condition reads $b/c<(b/c{)}^{*}<0$ such that spite is favored with the same value of $(b/c{)}^{*}$ as that for the one-layer network. If $\phi_{2,0}\neq 0$, depending on whether $\phi_{2,0}$ is positive or negative, spite occurs more easily or less easily for different ranges of *r*, relative to *r* = 1 (see Fig 2B). Interpretation of the condition for the spite behavior $b/c<(b/c{)}^{*}$ would be difficult if $(b/c{)}^{*}$ is positive. However, we again numerically confirmed that this unphysical situation can only occur when *r* is much larger than 1.

Finally, when $\theta_{1}-\theta_{3}=0$, cooperation is favored if $\theta_{2}>\frac{(r-1)\phi_{2,0}}{c}$.

In this manner, we can classify evolutionary outcomes with a linear analysis assuming weak selection. To assess which outcomes are frequent, we exhaustively considered unique two-layer networks with six individuals and all possible unique initial conditions with just one cooperator in layer 1 and one mutant in layer 2. Following the convention, we use the same initial condition (i.e., single cooperator and single mutant) in all the following numerical simulations as well. We set *c* = 1 in this and the following numerical simulations unless we state otherwise. We show the classification of the 2,763,739 unique pairs of two-layer network and initial condition in the left panel of Table 1. We have several observations: First, more than 80% of the unique pairs of network and initial condition yield negative $\theta_{1}-\theta_{3}$, implying spite behavior. In fact, this result is not specific to two-layer networks because setting *r* = 1 provides the condition for one-layer networks and the sign of $\theta_{1}-\theta_{3}$ is independent of *r*. Second, the condition for cooperation when $\theta_{1}-\theta_{3}=0$ is hard to interpret, but this case rarely occurs. Third, by additional numerical simulations, we confirmed that there is no case in which cooperation is selected for $b/c>(b/c{)}^{*}$ with a negative $(b/c{)}^{*}$ or the spite is selected for $b/c<(b/c{)}^{*}$ for a positive $(b/c{)}^{*}$ when *r* is sufficiently close to 0. The result that less than 20% networks with *N* = 6 individuals foster cooperation is underwhelming. However, we will later see that cooperation rather than spite is favored in a majority of larger networks.

**Table 1:** Classification of all unique pairs of the two-layer network with six individuals and the initial condition. (a) dB-dB. (b) dB-Bd. The marginal distribution of $\theta_{1}-\theta_{3}$ is the same between the dB-dB and dB-Bd updating rules. This is because $\theta_{1}-\theta_{3}$ only depends on the network structure and the updating rule used in layer 1, which are common between the dB-dB and dB-Bd rules.

dB-dB	$\phi_{2,0}$			
$\theta_{1}-\theta_{3}$	<0	=0	>0	Total
>0	6.40%	0.00%	10.98%	17.38%
=0	0.54%	0.00%	1.08%	1.61%
<0	21.46%	0.00%	59.54%	81.01%
Total	28.40%	0.00%	71.60%	100.00%

dB-Bd	$\phi_{2,0}$			
$\theta_{1}-\theta_{3}$	<0	=0	>0	Total
>0	8.23%	0.00%	9.15%	17.38%
=0	0.65%	0.00%	0.96%	1.61%
<0	38.29%	0.00%	42.72%	81.01%
Total	47.17%	0.00%	52.83%	100.00%

To explore which networks yield cooperation versus spite and enhancement of cooperation by coupling with the constant-selection layer, we first examined correlation between pairs of two outcome variables and three network metrics for single-layer networks with six nodes. The two outcome variables are $\theta_{1}-\theta_{3}$ and *n*_init,C_; the latter is the number of initial conditions out of the six initial conditions (i.e., choice of the single node which initially has the cooperator) for which $(b/c{)}^{*}>0$. The three network metrics are the mean degree, clustering coefficient, and mean closeness centrality, where the mean is taken over the six nodes. Focusing on the dB-dB rule, we find that $\theta_{1}-\theta_{3}$ and *n*_init,C_ are strongly positively correlated with each other, that these two outcome variables are strongly negatively correlated with the three network metrics, and that the three network metrics are strongly positively correlated with each other (see Text E in S1 Text for details). Therefore, the small mean degree, small clustering coefficient, and small mean closeness tend to enhance cooperation. We remark that the small mean degree enhancing cooperation is an established result [7,39]. The clustering coefficient and mean closeness are negatively correlated with $\theta_{1}-\theta_{3}$ and *n*_init,C_ even after partialing out the mean degree (see Text E in S1 Text).

For single-layer networks with six nodes fostering cooperation (i.e., $(b/c{)}^{*}>0$), we also find that the initial cooperator located at a node with a small degree, closeness, or local clustering coefficient promotes cooperation (i.e., by decreasing $(b/c{)}^{*}$). Finally, for two-layer networks with six nodes fostering cooperation, coupling with the constant-selection layer (i.e., layer 2) tends to allow enhancement of cooperation (i.e., large $\left|\text{d}(b/c{)}^{*}/\text{d}r\right|$) when the layer-1 network is dense (i.e., many edges). However, the edge density and other properties of the layer-2 network do not contribute to modulating the condition for cooperation. See Text E in S1 Text for details. While exploratory, these additional numerical results suggest that some network properties and initial conditions favor cooperation or enhance the effect of coupling with the constant-selection layer.

By numerical simulations of the stochastic evolutionary dynamics on some select two-layer networks with *N* = 6 individuals, we also verified that correlation between the strategy in layer 1 (i.e., cooperation or defection) and the type in layer 2 (i.e., resident or mutant) does not particularly build up during the stochastic process (see Text F in S1 Text for results). For example, even if we start from a correlated initial condition in which the initial sole cooperator in layer 1 and the initial sole mutant in layer 2 are the same individual, the strategy-type correlation between the two layers decays regardless of whether cooperation or defection eventually fixates.

#### 3.2.2 Evolution of mutants.

We turn to the condition under which the mutant is favored in the two-layer network, Eq. (4). Because $\theta_{2}<0$, we can rewrite Eq. (4) as


$$\begin{array}{c} r>r^{*}\equiv 1+\frac{b\left(\phi_{1,0}-\phi_{1,2}\right)+c\phi_{0,2}}{{\overset{―}{\theta }}_{2}}\equiv 1+A. \end{array}$$ (6)

The network-dependent constant $r^{*}$ is the value of *r* above which the mutant is favored over the resident in layer 2 of the two-layer network. This result is in stark contrast with that for one-layer networks because, in two-layer networks, both the payoff of the donation game and the structure of the two-layer networks in both layers influence the propensity that the mutant is favored through *A*. Remarkably, mutants whose fitness (i.e., *r*) is smaller than that of the resident type (i.e., 1) can be favored if *A* < 0. If *A* > 0, then *r* has to be sufficiently larger than 1 for the mutant to be favored. Another observation is that setting *b*/*c* to the $(b/c{)}^{*}$ value for the one-layer network does not lead to $r^{*}=1$ in general. Therefore, in contrast to the condition for cooperation, for which setting *r* = 1 recovered the one-layer network results, we do not have a mathematically solid baseline one-layer case for the constant selection layer. Furthermore, Eq. (6) indicates that *b* and *c* independently affect $r^{*}$, and whether an increase in *b* or *c* increases or decreases $r^{*}$ depends on the network structure.

To examine responsiveness of $r^{*}$ to the coupling with the donation game network layer, we investigated the same 2,763,739 unique pairs of two-layer network with *N* = 6 individuals and initial condition to calculate $dr^{*}/db=(\phi_{1,0}-\phi_{1,2})/{\overset{―}{\theta }}_{2}$ and $dr^{*}/dc=\phi_{0,2}/{\overset{―}{\theta }}_{2}$. We show the distribution of ($dr^{*}/db$, $dr^{*}/dc$) in Fig 3. The figure suggests that $dr^{*}/db$ and $dr^{*}/dc$ can have either sign depending on the network and initial condition. The effect of *b* and *c* on $r^{*}$ is modest; the average of $\left|dr^{*}/db\right|$ and $\left|dr^{*}/dc\right|$ over all the possible networks and initial condition is 0.0351 and 0.0665, respectively (with ranges $-0.211\leq dr^{*}/db\leq 0.294$ and $-0.262\leq dr^{*}/dc\leq 0.247$; the density of unique pairs of two-layer network and initial condition is invisibly small outside the ($dr^{*}/db$, $dr^{*}/dc$) region shown in the figure). The different stripes present in Fig 3 partially owe to the network structure and initial condition of layer 1 (see Text G in S1 Text). We conclude that the *b* and *c* values modestly, but consistently, affects $r^{*}$ in almost all cases.

**Fig 3:**
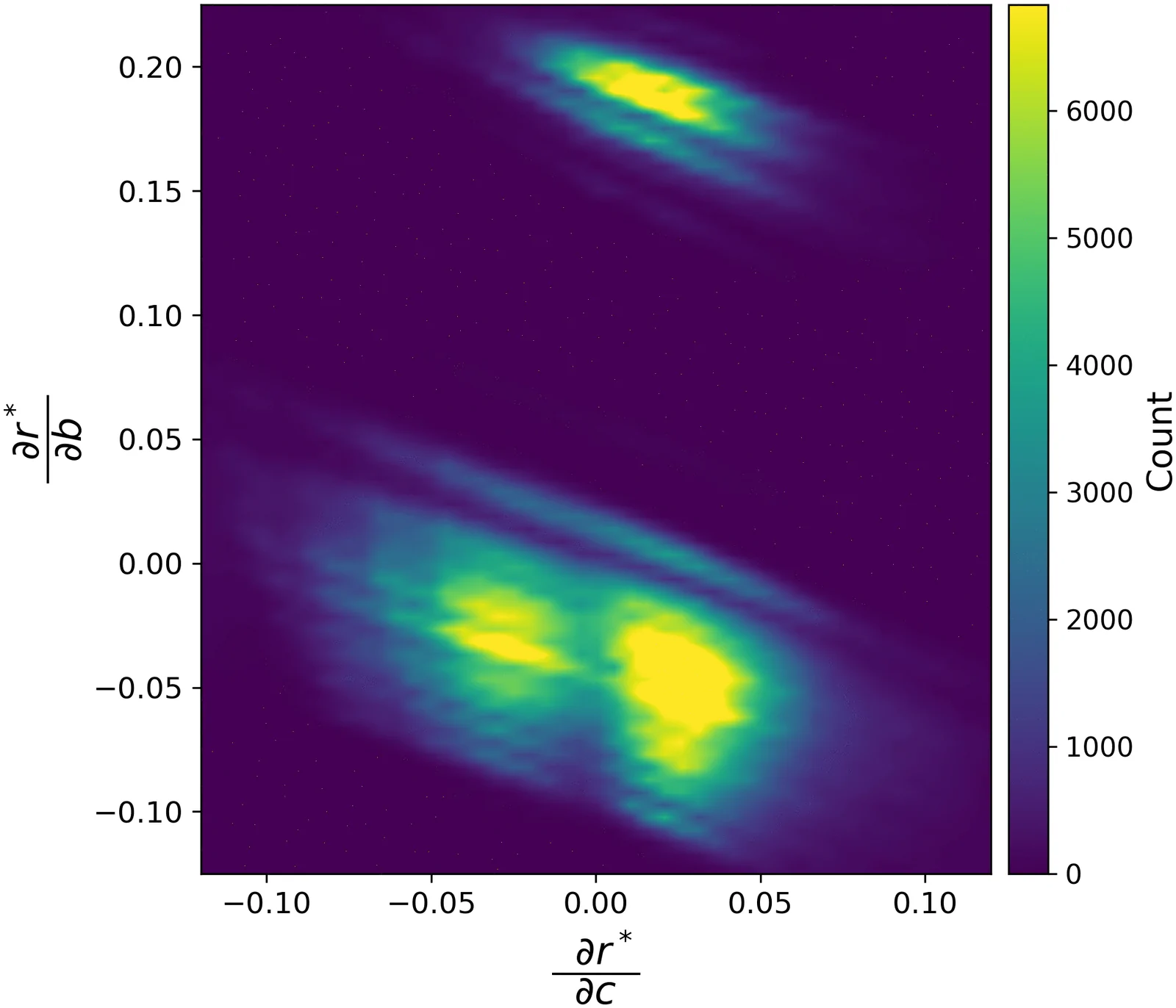
Distribution of $dr^{*}/db$ and $dr^{*}/dc$ over the 2,763,739 unique pairs of the two-layer network with six individuals and initial condition. This is a non-smoothed two-dimensional histogram.

### 3.3 Sample two-layer networks

To examine whether various two-layer networks favor cooperators or mutants and how, we examine four larger synthetic networks. The exact structure of these networks and the initial condition are shown in the left panels of Fig 4. The first two of them were used in [28]. First, consider a coupled ring network with *N* = 10 individuals shown in Fig 4A. When each layer evolves independently, one obtains $(b/c{)}^{*}=8/3$. When the two layers are coupled, *r* > 1 causes cooperation to emerge in layer 1 under a more generous condition than in the case of a one-layer network, i.e., $(b/c{)}^{*}<8/3$ (see the middle panel in Fig 4A). For example, we obtain $(b/c{)}^{*}=2.57$ when *r* = 2. Mathematically, in the limit of infinitely large two-layer ring network, $(b/c{)}^{*}$ decreases by 4(r−1)/c from the one-layer case (i.e., $(b/c{)}^{*}=1/2$) if the individual that initially cooperates in layer 1 is of the mutant type in layer 2 (see Text H in S1 Text). In other cases, the effect of the constant-selection layer to modulate $(b/c{)}^{*}$ diminishes as the size of the ring increases.

**Fig 4:**
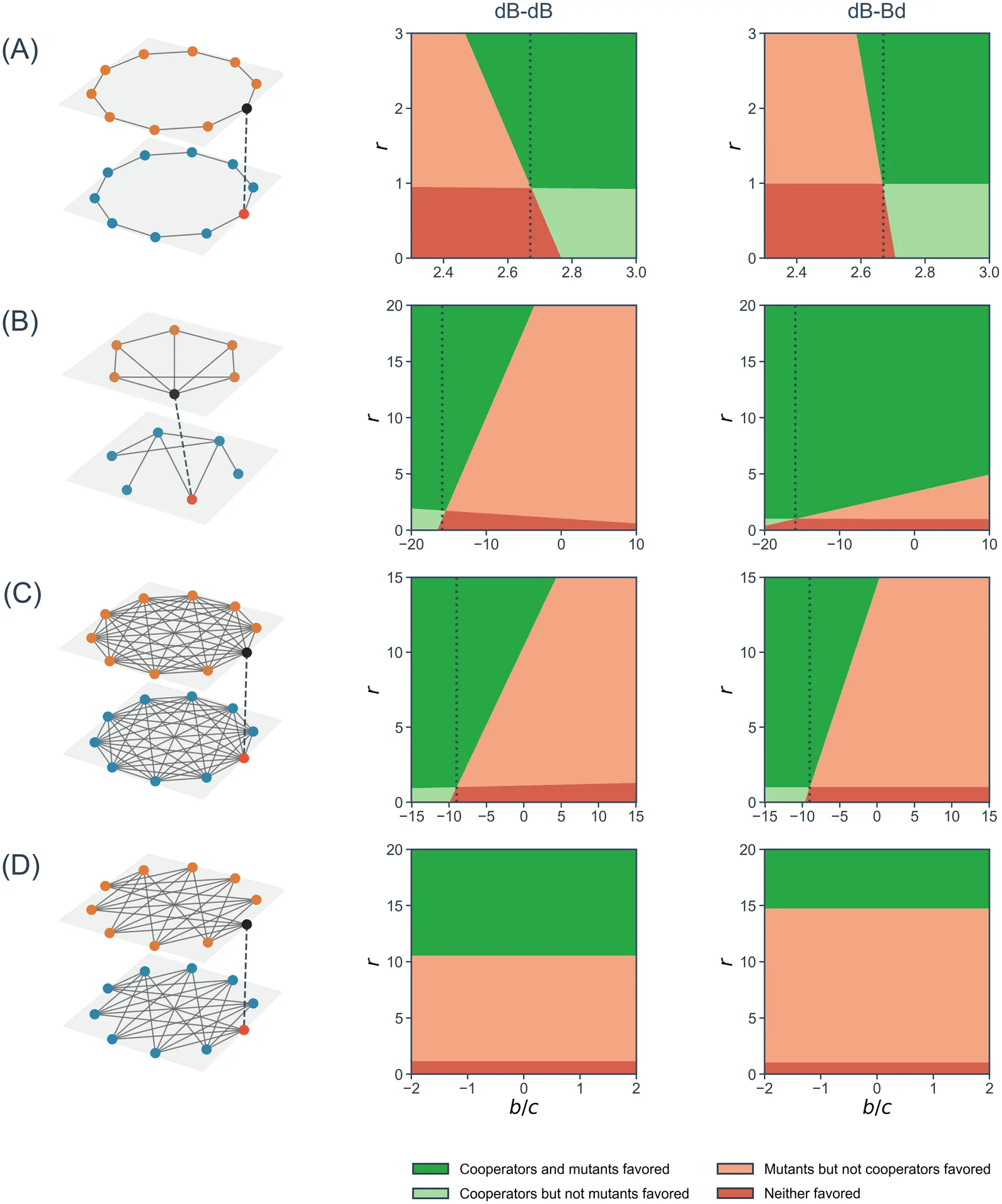
Selection of cooperators and mutants in four two-layer networks. Each row corresponds to the two-layer network and its initial condition visualized in the left panel. The colors of the replica nodes are the same as those used in Fig 1; orange, black, blue, and red represent cooperator, defector, resident, and mutant, respectively. The second column of each row of the figure shows the results under the dB-dB updating rule. The third column shows the results under the dB-Bd updating rule. Each colored region shows the (*b*/*c*, *r*) region in which selection favors or disfavors cooperators in layer 1 or mutants in layer 2. **(A)** Coupled ring networks with *N* = 10 individuals. We obtain $(b/c{)}^{*}=8/3$ for the uncoupled one-layer ring, shown by the dotted lines. **(B)** Coupled heterogeneous networks with *N* = 6. We obtain $(b/c{)}^{*}=-15.89$ for the uncoupled layer-1 network. **(C)** Coupled complete graphs with *N* = 10. The one-layer counterpart yields $(b/c{)}^{*}=-9$. **(D)** Coupled complete bipartite graphs with *N* = 10. The one-layer counterpart yields $(b/c{)}^{*}=\infty$.

Second, we investigated a heterogeneous two-layer network shown in Fig 4B. In this network, each layer promotes the evolution of spite (i.e., $(b/c{)}^{*}<0$) when the two layers are uncoupled. We find for this two-layer network that spite is favored with a smaller punishment (i.e., negative *b*/*c* values closer to 0) when *r* > 1 relative to the one-layer network and vice versa when *r* < 1.

Third, we investigated a two-layer network composed of two complete graph layers shown in Fig 4C. The results are qualitatively the same as those for the heterogeneous two-layer network investigated in Fig 4B. For this coupled complete graph, we analytically obtained the condition for favoring spite as $b/c<(b/c{)}^{*}=-(N-1)+\frac{r-1}{c}\cdot \frac{2(N-1{)}^{2}}{N(2N-3)}$; see Text I in S1 Text for the derivation. This result implies that spite can occur only in small coupled complete graphs because $(b/c{)}^{*}\to -\infty$ as N→∞.

Fourth, we investigated a two-layer network composed of two complete bipartite networks shown in Fig 4D. We find that selection favors cooperation if *r* is approximately larger than 10.5, regardless of the value of *b*/*c* (see the middle panel of Fig 4D). In contrast, a single complete bipartite network gives $(b/c{)}^{*}=\infty$, i.e., no cooperation.

Fifth, we also derived analytical results for the coupled star graph of arbitrary size (Text J in S1 Text).

These results are similar to those when the individuals play different donation games in two network layers of similar sizes [28]. Regardless of whether the second layer is subject to the social dilemma game or constant-selection dynamics, coupling of networks modulates the threshold value of *b*/*c*, i.e., $(b/c{)}^{*}$, for cooperation or spite. Quantitatively, in the coupled ring network (Fig 4A), $(b/c{)}^{*}$ decreases from 8/3 in the case of the one-layer ring network to 1.74 when the donation game with *b*/*c* = 10 is played on the other ring layer [28]. In our model, reduction of $(b/c{)}^{*}$ to 1.74 requires *r* = 10.78, which is by chance close to the aforementioned value of *b*/*c* = 10.

### 3.4 Random graphs

As a next test, we examined larger two-layer networks from two random network models. The first model is a two-layer Erdős-Rényi (ER) random graph. For each layer, we generate an instance of the ER random graph with *N* = 15 replica nodes with the probability of edge between each pair of nodes $p_{L}\in \{0.1,0.2,0.3,0.4,0.5\}$ for layer L∈{1,2}. The two network layers are independently generated. We evaluated all the 225 possible initial conditions with one cooperator in layer 1 and one mutant in layer 2 for each generated two-layer network with given *p*_1_ and *p*_2_.

We first analyzed the layer-1 ER random graphs as single-layer networks. Specifically, we counted the fraction of the layer-1 ER random graphs for which cooperation as opposed to spite is favored when $b/c>(b/c{)}^{*}$ with a positive value of $(b/c{)}^{*}$. We are interested in this case because, if this is the case, it is likely that one can choose a value of *r* that lowers $(b/c{)}^{*}$ in the two-layer network such that cooperation is facilitated by the coupling with the constant-selection network layer (i.e., layer 2). The upper part of Fig 5A shows the fraction of pairs of one-layer ER random graph and initial condition yielding $(b/c{)}^{*}>0$ as a single-layer network. Because this inquiry only concerns one-layer networks, we only show the result as a function of *p*_1_. We find that the all instances of networks enable cooperation when $p_{1}\leq 0.4$ and that 88% of them do so when *p*_1_ = 0.5. These values are much larger than for smaller networks; compare these numbers with those for networks with *N* = 6 individuals shown in Table 1.

**Fig 5:**
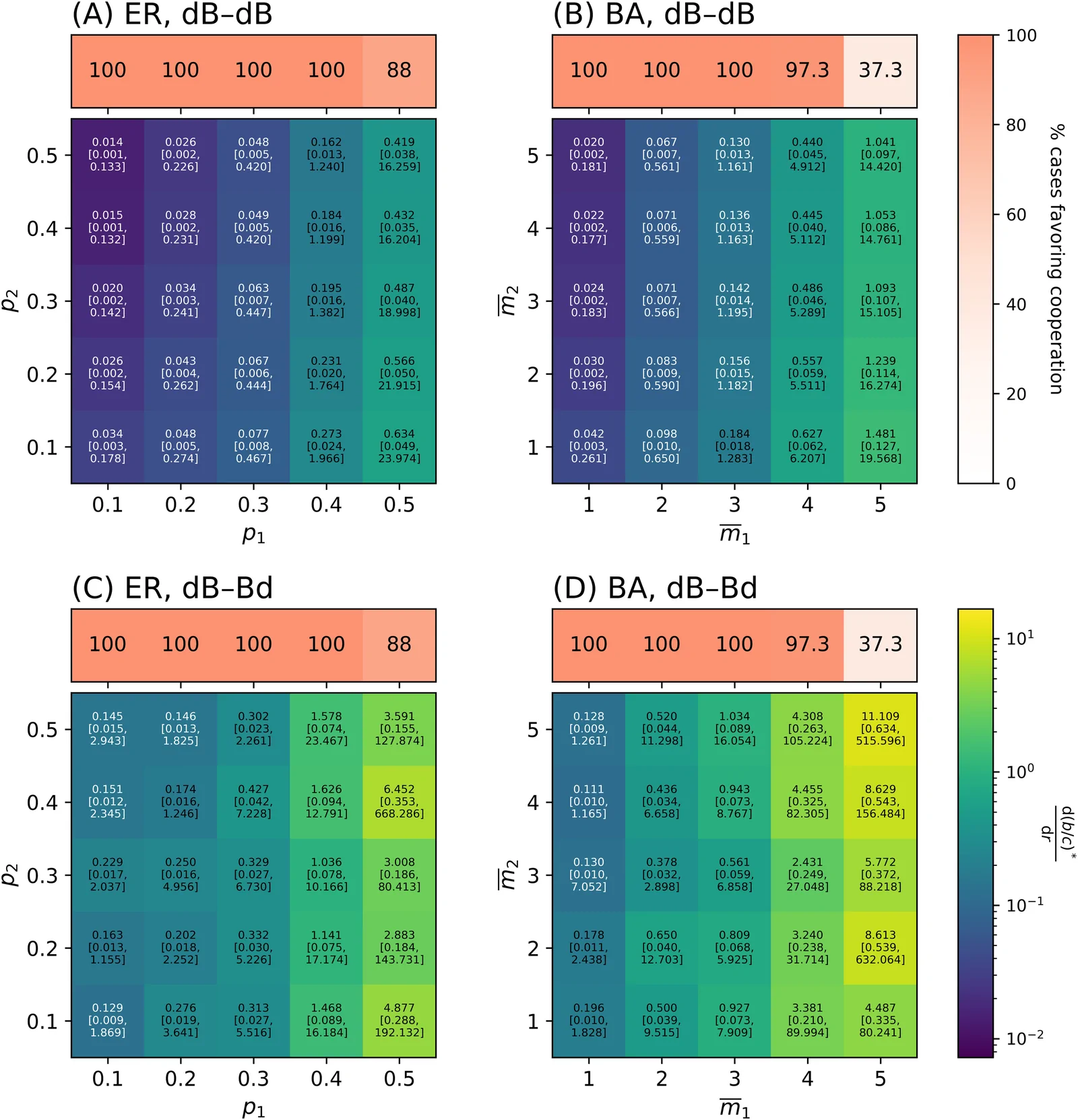
Evolution of cooperation in coupled ER and BA networks. **(A)** ER, dB-dB rule. **(B)** BA, dB-dB rule. **(C)** ER, dB-Bd rule. **(D)** BA, dB-Bd rule. The upper part of each panel shows the fraction of pairs of one-layer network and its initial condition that yield cooperation when $b/c>(b/c{)}^{*}$ with a threshold value $(b/c{)}^{*}>0$. The fraction values are the same between (A) and (C) and between (B) and (D) because this fraction only depends on the layer-1 network. The lower part of each panel shows the median along with the 5th and 95th percentiles (in square brackets) of $\left|\text{d}(b/c{)}^{*}/\text{d}r\right|$ for pairs of two-layer network and initial condition. Each two-layer network is composed of *N* = 15 individuals.

Next, we turned to two-layer networks. Specifically, we examined, among the runs yielding cooperation in layer 1, how much $(b/c{)}^{*}$ can be changed by the constant-selection dynamics. To quantify this effect, we measured $\left|\text{d}(b/c{)}^{*}/\text{d}r\right|$, i.e., how much $(b/c{)}^{*}$ moves by changing *r* for each pair of two-layer network and initial condition. For each pair of *p*_1_ and *p*_2_, we show in the lower part of Fig 5A the median along with the 5th and 95th percentiles of $\left|\text{d}(b/c{)}^{*}/\text{d}r\right|$. We show the median and percentiles instead of the mean and standard deviation because there are occasional outliers that yield a huge $\left|\text{d}(b/c{)}^{*}/\text{d}r\right|$ relative to typical pairs of two-layer network and initial condition. However, we have confirmed that the tendency that we are reporting with the median and percentiles remains similar when we measure the mean and standard deviation of $\left|\text{d}(b/c{)}^{*}/\text{d}r\right|$ (see Text K in S1 Text). In Fig 5A, we find that $\left|\text{d}(b/c{)}^{*}/\text{d}r\right|$ is larger for denser layer-1 networks (i.e., larger *p*_1_) and sparser layer-2 networks (i.e., smaller *p*_2_). The $(b/c{)}^{*}$ value is reasonably responsive to the changes in *r* (with median $\left|\text{d}(b/c{)}^{*}/\text{d}r\right|>0.4$) when *p*_1_ = 0.5 and modestly so (i.e., median $\left|\text{d}(b/c{)}^{*}/\text{d}r\right|>0.16$) when *p*_1_ = 0.4.

Given that most empirical contact networks are heterogeneous in terms of the node’s degree, we ran the same analysis for two-layer Barabási-Albert (BA) networks, where each new node added to the network layer *L* has ${\overset{―}{m}}_{L}$ nodes, making the average degree of each network layer approximately equal to $2{\overset{―}{m}}_{L}$ (see Methods for the procedure for generating networks). Fig 5B shows the proportion of network layer 1 that facilitates cooperation as a one-layer network (upper part) and the median and percentiles of $\left|\text{d}(b/c{)}^{*}/\text{d}r\right|$ (lower part). The results are qualitatively similar to those for the ER random graph. Specifically, one-layer BA networks favor cooperation in most cases if ${\overset{―}{m}}_{1}\leq 4$. Note that ${\overset{―}{m}}_{1}\leq 4$ does not imply particularly sparse networks because we are using networks with *N* = 15 nodes. Furthermore, $\left|\text{d}(b/c{)}^{*}/\text{d}r\right|$ is overall larger for the two-layer BA than ER networks. In particular, at ${\overset{―}{m}}_{1}=4$, a majority of BA networks support cooperation, and cooperation is substantially eased (i.e., median $\left|\text{d}(b/c{)}^{*}/\text{d}r\right|>0.44$) by coupling with another BA network layer subject to the constant-selection dynamics.

These results reinforce our main finding that coupling with the constant-selection dynamics can facilitate cooperation on networks.

### 3.5 Empirical networks

We examined two empirical two-layer networks, i.e., the Vickers–Chan 7th Graders (VC7) network with *N* = 29 individuals [49] and the Lazega Law Firm (LLF) network with *N* = 71 individuals [50] (see Methods for the network description). We show in the left panels of Fig 6 the (*b*/*c*, *r*) regions in which the cooperator or mutant is favored or disfavored. We obtain $(b/c{)}^{*}=-89.5$ for the VC7 two-layer network such that spite can evolve (see Fig 6A). By coupling with the constant-selection dynamics layer, every change in the *r* value by 1 changes $(b/c{)}^{*}$ value by 3.64 (4.07%). In the case of the LLF network, cooperation can be favored, and one obtains ${\left(\frac{b}{c}\right)}^{*}=52.4$ for the uncoupled layer-1 network. When the two layers are coupled, cooperation occurs more easily if *r* > 1 and less easily if *r* < 1, as shown in Fig 6C.

**Fig 6:**
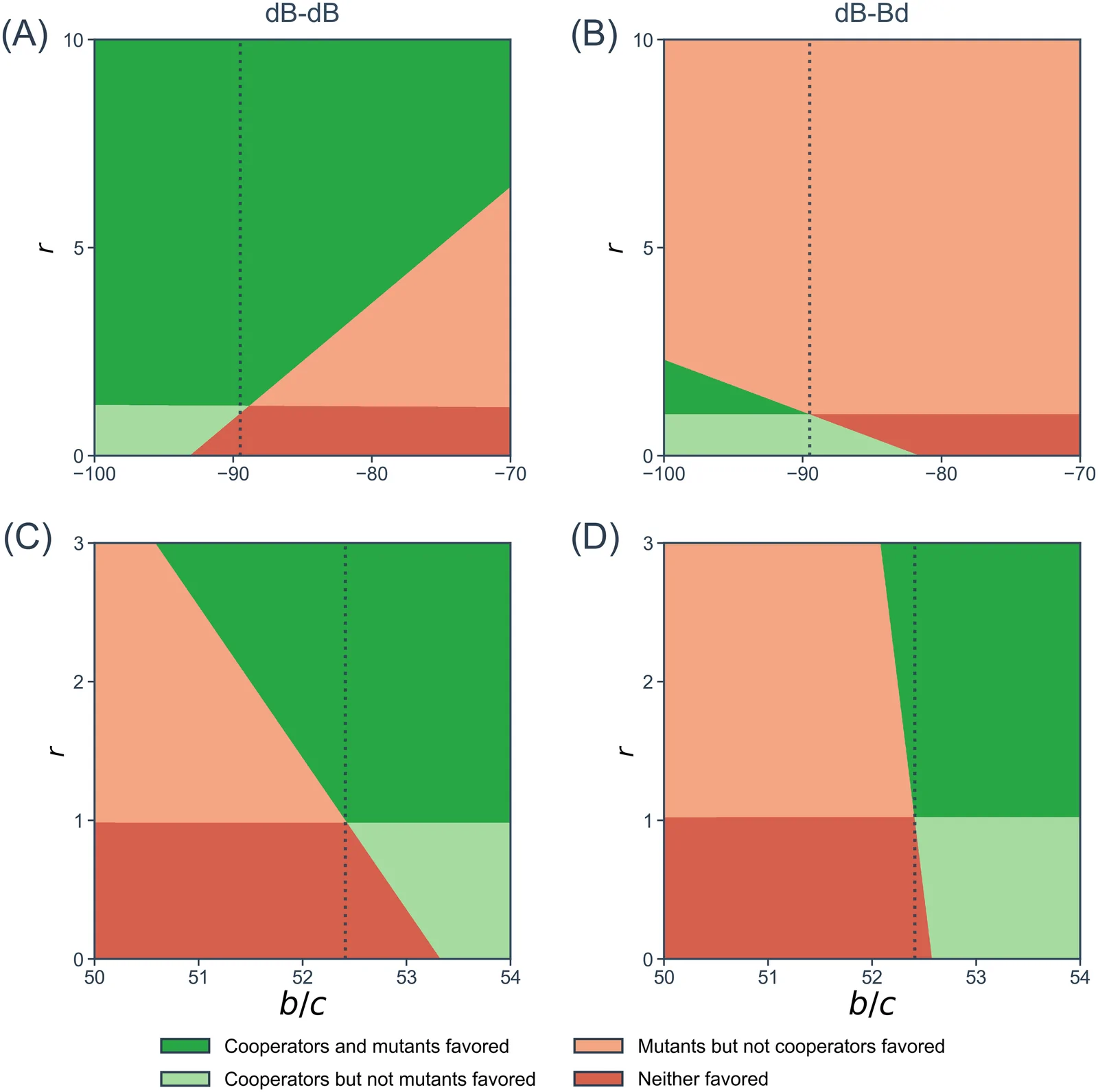
Parameter regions in which the cooperator or mutant is selected in empirical two-layer networks. The vertical lines represent the $(b/c{)}^{*}$ value for the uncoupled layer-1 network. **(A)** VC7, dB-dB. **(B)** VC7, dB-Bd. **(C)** LLF, dB-dB. **(D)** LLF, dB-Bd.

### 3.6 Birth-death (Bd) rule in the constant-selection layer

In the remainder of the Results section, including this section, we describe results of several robustness tests.

Constant selection on networks has most popularly been investigated under the Birth-death (Bd) updating rule, partly because the Bd rule amplifies the effect of selection in a majority of networks [40,51,52]. Therefore, we also derived the condition for favoring cooperators or mutants when layer 1 uses the dB rule and layer 2 uses the Bd rule, i.e., under the dB-Bd rule (see Text C in S1 Text).

Under the dB-Bd rule, because the evolutionary dynamics of the donation game is still governed by the dB rule, the condition under which cooperation is favored in layer 1 is given by Eq. (3). However, the value of $\phi_{2,0}^{\xi [1,2]}$ changes from the case of the dB-dB rule, affecting the value of $(b/c{)}^{*}$. Note that $\theta_{1}^{\xi [1]}$, $\theta_{2}^{\xi [1]}$, and $\theta_{3}^{\xi [1]}$ remain the same because they only depend on the network structure and updating rule in layer 1.

The results for the dB-Bd rule on two-layer networks with *N* = 6 individuals (right panel of Table 1), four sample networks (right panels of Fig 4), and two-layer ER and BA networks (Fig 5C and 5D) are qualitatively the same to those for the dB-dB rule (left panel of Table 1, middle panels of Fig 4, and Fig 5A and 5B). Notable quantitative differences are: (i) $(b/c{)}^{*}$ under the dB-Bd rule is 10.1 times more sensitive to variation in *r* than under the dB-dB rule in the two-layer network shown in Fig 4B. (ii) $\left|\text{d}(b/c{)}^{*}/\text{d}r\right|$ is substantially larger for the two-layer ER and BA networks under the dB-Bd than dB-dB rule across the network density parameters (i.e., *p*_1_, *p*_2_, ${\overset{―}{m}}_{1}$, and ${\overset{―}{m}}_{2}$). In the VC7 network, while $(b/c{)}^{*}$ increases as *r* increases under the dB-dB rule, $(b/c{)}^{*}$ decreases as *r* increases under the dB-Bd rule. Another observation on empirical two-layer networks is that $(b/c{)}^{*}$ is more responsive to the change in *r* under the dB-Bd than dB-dB rule for the VC7 network and vice versa for the LLF network. We conclude that the effect of layer-to-layer coupling on evolution of cooperation is overall similar between the dB-Bd and dB-dB rules, broadening the generality of our results.

### 3.7 Verification with stochastic numerical simulations

To explore the validity of our theory, we computed the fixation probability for cooperation by direct numerical simulations of the evolutionary dynamics with the strength of selection being δ=0.02 and δ=0.2. We used the two-layer ring network with *N* = 10 individuals shown in Fig 4A, two-layer BA networks with *N* = 15 and *N* = 100, and a two-layer ER random graph with *N* = 100. See Text L in S1 Text for the methods and results in detail.

Across these networks and the two updating rules (i.e., dB-dB and dB-Bd), we found that, for δ=0.02, the $(b/c{)}^{*}$ values estimated by the direct numerical simulations agree well with our theoretical prediction. In all these networks, coupling with the constant-selection layer promoted cooperation with an appropriate value of *r*. For δ=0.2, the numerically estimated $(b/c{)}^{*}$ considerably deviated from the theoretical prediction. This deviation is expected because our theory strictly holds true in the weak selection limit (i.e., δ→0). Nonetheless, the numerical results with δ=0.2 indicate that coupling with the constant-selection layer promotes cooperation as it does for δ=0.02.

It should be noted that the present numerical test includes larger networks (i.e., *N* = 100) than those used in the previous sections. We conclude that our main theoretical results are consistent with numerically simulated results of the evolutionary dynamics, including on larger networks.

For ER, BA, and empirical networks of comparable sizes, we also investigated the extent to which coupling with the constant-selection network layer enhances cooperation depending on the network structure and the effects of initial condition on $(b/c{)}^{*}$ (see Text E in S1 Text). The results are more diverse than but roughly consistent with those for networks with *N* = 6.

### 3.8 “Fixed benefits, fixed costs” goods scheme

We have considered the payoff scheme in which the cooperator pays the cost *c* and any neighbor of the cooperator receives a benefit *b*. This payoff scheme is the “pf goods” scheme in [38], except the difference regarding whether or not one divides the total payoff for each individual by the degree of the node, denoted by *k* in this section, for normalization. To further explore generality of our results, here we consider the “ff goods” scheme [38], with which a producer (i.e., cooperator) pays a fixed cost *c* irrespectively of *k*, and each neighbor receives benefit *b*/*k*. Therefore, the benefit produced by a producer, *b*, does not scale with the producer’s *k*, and the benefit *b* is divided equally by all the neighbors of the producer.

We derived the condition for favoring cooperators and mutants under the ff goods scheme and then examined the derived condition for the model and empirical networks that we have used (see Text M in S1 Text). The results are qualitatively the same as those under the pf goods scheme shown in the previous sections.

### 3.9 Fixation time

On one-layer networks, the cooperator’s fixation probability and fixation time are often in a trade-off relationship [53,54]. The advantages of two-layer networks over one-layer networks in promoting cooperation would be compromised if fixation times are excessively longer for two-layer than one-layer networks. Therefore, we have numerically investigated fixation time in two-layer networks in some representative scenarios. To make a fair comparison between the one-layer and two-layer cases, we declared the fixation of cooperation once it is attained in layer 1 even if the resident or mutant type has not fixated in layer 2. We computed the fixation time for cooperators as the average over the runs for which the cooperator has fixated.

We show the mean fixation time for cooperators for different networks and values of *b*/*c*, *r*, and δ (i.e., selection strength) in Text N in S1 Text. For four synthetic networks, we find that the fixation time does not substantially increase (i.e., the increase is at most ≈10%) in two-layer networks relative to one-layer networks including the case in which cooperation is facilitated by the two-layer networks. For the VC7 network, the fixation of cooperation needs at most 2.8 times more time when the two network layers are coupled than uncoupled.

## 4 Discussion

The donation game and constant selection are two widely studied models of evolutionary dynamics. Both models allow substantial theoretical analysis, in particular via fixation theory, providing general and quantitative understanding. These two models have mostly been studied in isolation. They capture different social processes that people and institutions may simultaneously encounter. In particular, constant-selection dynamics is equivalent to a biased voter model, a standard model of competing opinions studied for decades [34–37]. We modeled this setting using a two-layer network in which each individual is involved in the donation game in one network layer and undergoes constant-selection dynamics in the other layer. By adapting the recently developed multilayer fixation theory [28], we showed that coupling with the layer can enhance prosociality in the donation-game layer. This effect is analogous to the cooperation enhancement previously reported when both layers host donation games [28]. In other words, enhancing prosociality does not require coupling two dilemma layers. Coupling a dilemma layer to a non-dilemmatic layer can also produce a cross-layer spillover effect that shifts the effective condition for cooperation in the dilemma layer. In our model, the condition for favoring cooperation (i.e., $(b/c{)}^{*}$) is reasonably responsive to the bias in the strength of the two opinions competing in the other layer (i.e., *r*). Conversely, the donation-game parameters (i.e., *b* and *c*) spill over to affect the condition for favoring the mutant type in the constant-selection layer. We tested robustness across different networks, updating rules, and goods (i.e., payoff) schemes. Extension to the case of more than two layers [23], different types of non-dilemma or dilemma dynamics in layer 2 [26,27,29–31], layers of hypergraphs modeling group interactions [10,22,25,55,56], different updating rules such as a pairwise comparison rule [7,57,58], and different initial conditions may be fruitful.

Engineering the structure of one network layer to enhance cooperation in the other network layer was briefly examined in [28] for the case of donation games in different network layers. Such an intervention method may be similarly effective at enhancing cooperation in our model. However, clarifying how one should manipulate layer 2, in which the constant-selection dynamics occurs, awaits investigation. In addition, the correspondence between the constant-selection dynamics and the biased voter model opens further avenues for engineering cooperation. Our results suggest that deploying a new opinion or fad currently dominated by a resident opinion may enhance cooperation in the other network layer. One may be able to target the initial adopters of the new opinion to further facilitate cooperation. Furthermore, cooperation may be better enhanced by introducing zealots, i.e., a small number of individuals who stick to their opinion regardless of social influence [59–61]. Introducing zealot cooperators [62] guarantees fixation of cooperation in finite populations [63]. In practice, however, it may be less costly to introduce zealots in the opinion layer to indirectly promote cooperation than to introduce zealot cooperators directly.

We assumed that all the individuals generate payoffs in both layers and that the payoff summed over the two layers, $F_{i}$, determines fecundity of the *i*th individual in both layers. This corresponds to a simple type of multilayer networks called multiplex networks. In fact, multilayer network research has advocated various ways in which different network layers depend on each other, driven by real-world examples [19–22]. Extending the present framework, as well as prior analyses of donation games in both layers [28] and constant-selection dynamics in both layers [64], to various types of multilayer network setting with analytical rigor warrants future work. First, inter-layer coupling may be weaker or unidirectional. A weaker inter-layer coupling implies that the fecundity in one layer more weakly depends on the payoff in the other layer [24,65]. The unidirectional coupling implies that the payoff in one layer influences the fecundity in the other layer, but not vice versa. Second, some nodes may be missing in one layer. Third, a “network of networks” setting would include more than two layers and specify which layer pairs interact. A network of networks may better reflect human behavior when individuals playing a social dilemma game use information from selected other social contexts, rather than from all layers equally.

The present work has two main limitations. First, as in fixation-based analyses of multilayer networks [28,64], it is computationally demanding to check conditions for favoring cooperators and mutants. Therefore, our present analysis is limited to small two-layer networks. However, we analyzed networks of different sizes to the best of our effort and also carried out robustness tests along additional dimensions. Second, as for a plethora of work in mathematically founded studies of evolutionary game and graph theory, we assumed weak selection. Focusing on simple network structure such as the coupled complete graph and the coupled ring network to analyze the case of strong selection may be a useful analytical approach. Our main message is that coupling a dilemma layer with the constant-selection dynamics, or the biased voter model dynamics, can enhance prosociality in the dilemma layer, similar to the case of coupling two dilemma layers. An important direction for future research is to test this prediction by controlled experiments and data-driven multilayer network modeling.

## 5 Methods

### 5.1 Generation of two-layer BA networks

For each layer L∈{1,2}, the initial condition of the network growth process was the star graph on ${\overset{―}{m}}_{L}+1$ nodes. Then, we grew the network by sequentially adding nodes with ${\overset{―}{m}}_{L}$ edges according to the preferential attachment rule [66]. For each ${\overset{―}{m}}_{L}\in \{1,2,3,4,5\}$, we generated 5 instances of BA networks with *N* = 15 nodes for each layer, yielding 25 two-layer BA networks. We then uniformly randomly shuffled the node label of layer 2 to avoid the tendency that individuals with small indices (i.e., joining the network early in the growth process) are hubs in both layers such that the node’s degree is positively correlated between the two layers.

### 5.2 Empirical two-layer networks

We have used two empirical two-layer networks. The first is the Vickers–Chan 7th Graders (VC7) network, which comprises two layers representing academic and friendship connections among 29 seventh-grade students from a school in Victoria, Australia [49]. We arbitrarily assigned the academic connection network to layer 1. The VC7 network has 126 and 152 edges in layer 1 and 2, respectively. The second network is the Lazega Law Firm (LLF) network, in which the two network layers represent professional and cooperative relationships among 71 partners within the firm [50]. We arbitrarily assigned the professional relation network to layer 1. The LLF network has 717 and 726 edges in layer 1 and 2, respectively.

## Supporting information

S1 TextSupporting information for “Coupling with opinion dynamics promotes prosocial behavior in multilayer networks”. The file contains Texts A–N, Figs A–N, and Tables A and B, listed below.**Text A.** Derivation of the condition under which the cooperator or mutant is favored. **Text B.** Evolution of the cooperator and mutant under the dB-dB rule. **Text C.** Evolution of cooperator and mutant under the dB-Bd rule. **Text D.** Interpretation of Eqs. (3) and (4). **Text E.** Impact of the network structure and initial condition on promotion of cooperation. **Text F.** Strategy-type correlation between the two layers during evolutionary dynamics. **Text G.** Effects of the network structure and initial condition in layer 1 on $\frac{\partial r^{*}}{\partial b}$ and $\frac{\partial r^{*}}{\partial c}$. **Text H.** Evolution of the cooperator and mutant on the coupled ring network. **Text I.** Evolution of the cooperator and mutant on the coupled complete graph. **Text J.** Evolution of the cooperator and mutant on the coupled star graph. **Text K.** Mean and standard deviation of $\left|\frac{d(b/c{)}^{*}}{dr}\right|$ for two-layer ER and BA networks with *N* = 15 individuals. **Text L.** Numerical simulations of the stochastic evolutionary dynamics. **Text M.** “Fixed benefits, fixed costs” goods scheme. **Text N.** Fixation time. **Fig A.** Association between prosociality and network structure for one-layer networks with six nodes under the dB-dB rule. **Fig B.** Correlation between $(b/c{)}^{*}$ and the initial condition for the six-node networks that yield $(b/c{)}^{*}>0$ regardless of the initial condition. **Fig C.** Dynamics of the correlation between the states in layer 1 (i.e., defection or cooperation) and those in layer 2 (i.e., resident or mutant). **Fig D.** Distribution of $\frac{\partial r^{*}}{\partial b}$ and $\frac{\partial r^{*}}{\partial c}$ over layer-2 networks and their initial conditions. **Fig E.** Two-layer star graph and the initial condition that we analyze. **Fig F.** Mean, μ, and standard deviation, σ, of $\left|d(b/c{)}^{*}/dr\right|$ for two-layer ER and BA networks with *N* = 15 individuals. **Fig G.** Fixation probability for cooperation compared between the weak selection theory and direct numerical simulations on the ring network with *N* = 10 and a BA network with *N* = 15. **Fig H.** Parameter regions in which the cooperator or mutant is selected in two-layer networks with *N* = 100 individuals. **Fig I.** Fixation probability for cooperation compared between the weak selection theory and direct numerical simulations on networks with *N* = 100. **Fig J.** Parameter regions in which the cooperator or mutant is favored under the ff good scheme for the two-layer network used in Fig 4B in the main text (replicated in the left panel of this figure). **Fig K.** Evolution of cooperation in two-layer ER and BA networks under the ff goods scheme. **Fig L.** Mean, μ, and standard deviation, σ, of $\left|d(b/c{)}^{*}/dr\right|$ for two-layer ER and BA networks with *N* = 15 individuals under the ff goods scheme. **Fig M.** Parameter regions in which the cooperator or mutant is selected in empirical two-layer networks under the ff goods scheme. **Fig N.** Mean fixation time for cooperation in the four synthetic networks and the VC7 network. **Table A.** Relationship between $\left|d(b/c{)}^{*}/dr\right|$ and properties of the two-layer network. **Table B.** Correlation between $(b/c{)}^{*}$ and the initial condition for larger networks.(PDF)
