# Supplementary material for: Coupling with opinion dynamics promotes prosocial behavior in multilayer networks
Source: PLoS Comput Biol. 2026 Sep 23;22(9):e1014810. doi: 10.1371/journal.pcbi.1014810 (PMC13632918; doi:10.1371/journal.pcbi.1014810)
Supplement: Supplementary file 1 [file pcbi.1014810.s001.pdf]

S1 Text

Supporting Information for:

Coupling with opinion dynamics promotes prosocial behavior in multilayer networks

Jnanajyoti Bhaumik, Naoki Masuda

Text A Derivation of the condition under which the cooperator or mutant is favored

A.1 Replacement events and the fixation axiom

We use the notations and assumptions, particularly regarding the replacement events and fixation axiom, in Ref. [1], which was originally introduced in Ref. [2] and also used in Ref. [3]. We consider a population with two layers. In each time step, we choose a replacement event, (R, α), which consists of a pair $\left(R^{[L]}, \alpha^{[L]}\right)$ for each layer, $L \in \{1, 2\}$, where $R^{[L]}$ is the set of 'offspring' nodes that are chosen for replacement, and $\alpha^{[L]} : R^{[L]} \to \{1, \ldots, N\}$ is the offspring-to-parent map in layer L. We refer to cooperators and mutants as type A, and defectors and residents as type B. We denote by $p_{(R,\alpha)}(\boldsymbol{x})$ the probability of choosing (R, α) in state $\boldsymbol{x} \in \{0, 1\}^N \times \{0, 1\}^N$, where each entry of $\boldsymbol{x}$, denoted by $x_i^{[L]}$, is 1 and 0 if individual i has type A and B, respectively, in layer L. In each layer L, the map $\alpha^{[L]} : R^{[L]} \to \{1, \ldots, N\}$ extends to a map $\tilde{\alpha}^{[L]} : \{1, \ldots, N\} \to \{1, \ldots, N\}$ defined by $\tilde{\alpha}^{[L]}(i) = \alpha^{[L]}(i)$ if $i \in R^{[L]}$ and $\tilde{\alpha}^{[L]}(i) = i$ if $i \notin R^{[L]}$. For any state $\boldsymbol{x} \in \{0, 1\}^N \times \{0, 1\}^N$, we write $\boldsymbol{x}^{[L]} \in \{0, 1\}^N$ for the state of the population in layer $L \in \{1, 2\}$. This extension of α, denoted $\tilde{\alpha}$, gives an updated state $\boldsymbol{x}_{\tilde{\alpha}} \in \{0, 1\}^N \times \{0, 1\}^N$ defined by $(\boldsymbol{x}_{\tilde{\alpha}})_i^{[L]} = \boldsymbol{x}_{\tilde{\alpha}^{[L]}(i)}^{[L]}$. In the case of the dB and Bd processes, $R^{[L]}$ is a singleton set containing the individual i_0 to be replaced by its parent j, and we obtain $\tilde{\alpha}^{[L]}(i) = i$ if $i \neq i_0$ and $\tilde{\alpha}^{[L]}(i_0) = j$.

For $\boldsymbol{x}, \boldsymbol{y} \in \{0, 1\}^N \times \{0, 1\}^N$, we then have the state transition probability as follows:

$$P_{\boldsymbol{x}\to\boldsymbol{y}} = \sum_{(R,\alpha) \text{ s.t. } \boldsymbol{x}_{\tilde{\alpha}}=\boldsymbol{y}} p_{(R,\alpha)}(\boldsymbol{x}). \tag{S1}$$

In addition to being smooth, we assume that the replacement rule satisfies the following fixation axiom [1]:

Axiom 1 (Fixation axiom) *There exists $(i_1, i_2) \in \{1, \ldots, N\} \times \{1, \ldots, N\}$, an integer $m \geq 1$, and a sequence of replacement events $\{(R_k, \alpha_k)\}_{k=1}^m$ such that:*

(i) $p_{(R_k,\alpha_k)}(\boldsymbol{x}) > 0$ for every $k \in \{1, \ldots, m\}$ and $\boldsymbol{x} \in \{0, 1\}^N \times \{0, 1\}^N$.

(ii) For each $L \in \{1, 2\}$, there exists k_L such that $i_L \in R_{k_L}^{[L]}$.

(iii) For $L \in \{1, 2\}$, we have $\tilde{\alpha}_1^{[L]} \circ \tilde{\alpha}_2^{[L]} \circ \cdots \circ \tilde{\alpha}_m^{[L]}(j) = i_L$ for every $j \in \{1, \ldots, N\}$.

A.2 Weak selection and a mutation-modified Markov chain

We assume weak selection. In Ref. [1], they considered a mutation-modified Markov chain obtained by sending each absorbing state to a fixed, transient state $\boldsymbol{\xi} \in \{0, 1\}^N \times \{0, 1\}^N$ with mutation probability $u > 0$ in each time step. With probability $1 - u$, the chain remains in the same absorbing state in each time

step. The benefit of this approach is that the mutation-modified Markov chain has a unique stationary distribution that facilitates further calculations. This chain has transition probabilities given by

$$P^{\circlearrowleft\{\boldsymbol{\xi}\}}_{\boldsymbol{x}\to\boldsymbol{y}} = \begin{cases} u & \text{if } \boldsymbol{x} \in \{\boldsymbol{AA}, \boldsymbol{AB}, \boldsymbol{BA}, \boldsymbol{BB}\}, \boldsymbol{y} = \boldsymbol{\xi}, \\ (1-u)P_{\boldsymbol{x}\to\boldsymbol{y}} & \text{if } \boldsymbol{x} \in \{\boldsymbol{AA}, \boldsymbol{AB}, \boldsymbol{BA}, \boldsymbol{BB}\}, \boldsymbol{y} \neq \boldsymbol{\xi}, \\ P_{\boldsymbol{x}\to\boldsymbol{y}} & \text{if } \boldsymbol{x} \notin \{\boldsymbol{AA}, \boldsymbol{AB}, \boldsymbol{BA}, \boldsymbol{BB}\}. \end{cases} \tag{S2}$$

In Eq. (S2), $\boldsymbol{AA}$ is the state in which all the individuals are of type A in both layers, and $\boldsymbol{AB}$ is the state in which all individuals are of type A in layer 1 and type B in layer 2. States $\boldsymbol{BA}$ and $\boldsymbol{BB}$ are similarly defined. The probability that i transmits its offspring to j in layer L and in state $\boldsymbol{x}$ is

$$e^{[L]}_{ij}(\boldsymbol{x}) := \sum_{(R,\alpha) \text{ s.t. } \alpha^{[L]}(j)=i} p_{(R,\alpha)}\,(\boldsymbol{x})\,. \tag{S3}$$

We recall that δ represents the strength of selection. If $\delta = 0$, then we have neutral drift, which is denoted by $\circ$. We denote by $e^{\circ[L]}_{ij}$ the probability that i transmits its offspring to j under neutral drift in layer L. We also denote by $\pi^{[L]}_i$ the reproductive value (RV) of individual i [4–8] in layer L under neutral drift. We obtain

$$\sum_{j=1}^{N} e^{\circ[L]}_{ij}\pi^{[L]}_j = \pi^{[L]}_i \sum_{j=1}^{N} e^{\circ[L]}_{ji} \tag{S4}$$

and

$$\sum_{i=1}^{N} \pi^{[L]}_i = 1. \tag{S5}$$

The RV-weighted frequency of state $\boldsymbol{x}$ in layer L is denoted by

$$\widehat{x}^{[L]} := \sum_{i=1}^{N} \pi^{[L]}_i x^{[L]}_i. \tag{S6}$$

The change in the RV-weighted frequency in layer L due to selection in one step of the evolutionary dynamics is

$$\widehat{\Delta}^{[L]}_{\text{sel}}(\boldsymbol{x}) = \sum_{i=1}^{N}\sum_{j=1}^{N} \pi^{[L]}_i (x_j - x_i) e^{[L]}_{ji}(\boldsymbol{x}). \tag{S7}$$

The change in the RV-weighted frequency in one time step due to both mutation and selection is given by

$$\widehat{\Delta}^{[L]}(\boldsymbol{x}) = \begin{cases} u\left(\widehat{\xi}^{[L]} - 1\right) & \text{if } \boldsymbol{x}^{[L]} = \boldsymbol{A},\ \boldsymbol{x}^{[-L]} \in \{\boldsymbol{A}, \boldsymbol{B}\}, \\ u\widehat{\xi}^{[L]} & \text{if } \boldsymbol{x}^{[L]} = \boldsymbol{B},\ \boldsymbol{x}^{[-L]} \in \{\boldsymbol{A}, \boldsymbol{B}\}, \\ \widehat{\Delta}^{[L]}_{\text{sel}}(\boldsymbol{x}) & \text{if } \boldsymbol{x} \notin \{\boldsymbol{AA}, \boldsymbol{AB}, \boldsymbol{BA}, \boldsymbol{BB}\}, \end{cases} \tag{S8}$$

where $-L$ means the other layer (i.e., layer 2 if $L = 1$ and layer 1 if $L = 2$); $\widehat{\xi}^{[L]} = \sum_{i=1}^{N} \pi^{[L]}_i \xi^{[L]}_i$ is the RV-weighted frequency of type A in state $\boldsymbol{\xi}$ in layer L.

Next, we compute the expectation of the change in the RV-weighted frequency, which will enable us to compute the fixation probability of the mutation-modified Markov chain under the rare mutation condition. Because we are computing the expectation with respect to the stationary probability distribution, we obtain

$$\mathbb{E}_{\circlearrowleft(\boldsymbol{\xi})}[\widehat{\Delta}^{[L]}] = 0. \tag{S9}$$

Equation (S9) yields

$$\mathbb{E}_{\circlearrowleft(\boldsymbol{\xi})}\left[\widehat{\Delta}_{\mathrm{sel}}^{[L]}\right]=\begin{cases}u\pi_{\circlearrowleft(\boldsymbol{\xi})}\left(\{\boldsymbol{A}\}\times\{\boldsymbol{A},\boldsymbol{B}\}\right)\left(1-\widehat{\xi}^{[L]}\right)-u\pi_{\circlearrowleft(\boldsymbol{\xi})}\left(\{\boldsymbol{B}\}\times\{\boldsymbol{A},\boldsymbol{B}\}\right)\widehat{\xi}^{[L]} & \text{if } L=1,\\ u\pi_{\circlearrowleft(\boldsymbol{\xi})}\left(\{\boldsymbol{A},\boldsymbol{B}\}\times\{\boldsymbol{A}\}\right)\left(1-\widehat{\xi}^{[L]}\right)-u\pi_{\circlearrowleft(\boldsymbol{\xi})}\left(\{\boldsymbol{A},\boldsymbol{B}\}\times\{\boldsymbol{B}\}\right)\widehat{\xi}^{[L]} & \text{if } L=2.\end{cases} \tag{S10}$$

We define $\rho_{\boldsymbol{A}}^{[L]}(\boldsymbol{\xi})$ as the fixation probability of the mutation-modified Markov chain reaching a state in which all individuals have type A in layer L when the initial state of the network is $\boldsymbol{\xi}$. By the known rare-mutation results [2,3], we have

$$\lim_{u\to 0}\pi_{\circlearrowleft(\boldsymbol{\xi})}\left(\{\boldsymbol{A}\}\times\{\boldsymbol{A},\boldsymbol{B}\}\right)=\rho_{\boldsymbol{A}}^{[1]}(\boldsymbol{\xi}), \tag{S11}$$

$$\lim_{u\to 0}\pi_{\circlearrowleft(\boldsymbol{\xi})}\left(\{\boldsymbol{B}\}\times\{\boldsymbol{A},\boldsymbol{B}\}\right)=1-\rho_{\boldsymbol{A}}^{[1]}(\boldsymbol{\xi}), \tag{S12}$$

$$\lim_{u\to 0}\pi_{\circlearrowleft(\boldsymbol{\xi})}\left(\{\boldsymbol{A},\boldsymbol{B}\}\times\{\boldsymbol{A}\}\right)=\rho_{\boldsymbol{A}}^{[2]}(\boldsymbol{\xi}), \tag{S13}$$

$$\lim_{u\to 0}\pi_{\circlearrowleft(\boldsymbol{\xi})}\left(\{\boldsymbol{A},\boldsymbol{B}\}\times\{\boldsymbol{B}\}\right)=1-\rho_{\boldsymbol{A}}^{[2]}(\boldsymbol{\xi}). \tag{S14}$$

Then, we can write down the fixation probability [1] as

$$\rho_{\boldsymbol{A}}^{[L]}(\boldsymbol{\xi})=\widehat{\xi}^{[L]}+\left.\frac{\mathrm{d}}{\mathrm{d}u}\right|_{u=0}\mathbb{E}_{\circlearrowleft(\boldsymbol{\xi})}\left[\widehat{\Delta}_{\mathrm{sel}}^{[L]}\right]. \tag{S15}$$

Because $\pi_{\circlearrowleft(\boldsymbol{\xi})}, \widehat{\xi}^{[L]}$, and u are smooth in terms of δ and u, we have [1,2]

$$\left.\frac{\mathrm{d}}{\mathrm{d}\delta}\rho_{\boldsymbol{A}}^{[L]}(\boldsymbol{\xi})\right|_{\delta=0}=\left.\frac{\mathrm{d}}{\mathrm{d}u}\right|_{u=0}\mathbb{E}_{\circlearrowleft(\boldsymbol{\xi})}^{\circ}\left[\left.\frac{\mathrm{d}}{\mathrm{d}\delta}\right|_{\delta=0}\widehat{\Delta}_{\mathrm{sel}}^{[L]}\right]. \tag{S16}$$

Finally, for $\boldsymbol{x}\notin\{\boldsymbol{AA},\boldsymbol{AB},\boldsymbol{BA},\boldsymbol{BB}\}$, consider the rare-mutation conditional (RMC) distribution [1] given by

$$\pi_{\mathrm{RMC}(\boldsymbol{\xi})}(\boldsymbol{x}):=K\left.\frac{\mathrm{d}}{\mathrm{d}u}\right|_{u=0}\pi_{\circlearrowleft(\boldsymbol{\xi})}(\boldsymbol{x}), \tag{S17}$$

where

$$K:=\left(\sum_{\boldsymbol{y}\notin\{\boldsymbol{AA},\boldsymbol{AB},\boldsymbol{BA},\boldsymbol{BB}\}}\left.\frac{\mathrm{d}}{\mathrm{d}u}\right|_{u=0}\pi_{\circlearrowleft(\boldsymbol{\xi})}(\boldsymbol{y})\right)^{-1}. \tag{S18}$$

The following lemma holds true:

Lemma 1 *For any function $\varphi:\{0,1\}^N\times\{0,1\}^N\to\mathbb{R}$,*

$$\begin{aligned}\mathbb{E}_{RMC(\boldsymbol{\xi})}^{\circ}[\varphi]=K^{\circ}&\Big[\varphi(\boldsymbol{\xi})-\widehat{\xi}^{[1]}\widehat{\xi}^{[2]}\varphi(\boldsymbol{A},\boldsymbol{A})-\widehat{\xi}^{[1]}(1-\widehat{\xi}^{[2]})\varphi(\boldsymbol{A},\boldsymbol{B})\\ &-(1-\widehat{\xi}^{[1]})\widehat{\xi}^{[2]}\varphi(\boldsymbol{B},\boldsymbol{A})-(1-\widehat{\xi}^{[1]})(1-\widehat{\xi}^{[2]})\varphi(\boldsymbol{B},\boldsymbol{B})\Big]\\ &+\sum_{(R,\alpha)}p_{(R,\alpha)}^{\circ}\mathbb{E}_{RMC(\boldsymbol{\xi})}^{\circ}[\varphi_{\tilde{\alpha}}].\end{aligned} \tag{S19}$$

We recall that $\varphi_{\tilde{\alpha}}$ is the extension of the off-spring to parent map that maps the nodes in R to their parents and the nodes not in R to themselves. As indicated in Ref. [1], the proof of Eq. (S19) is an adaptation of the proof of Lemma 1 in Ref. [3]. Note that $\varphi(\boldsymbol{x})$ in Lemma 1 in Ref. [3] is replaced with function $\varphi(\boldsymbol{\xi})-\widehat{\xi}^{[1]}\widehat{\xi}^{[2]}\varphi(\boldsymbol{A},\boldsymbol{A})-\widehat{\xi}^{[1]}(1-\widehat{\xi}^{[2]})\varphi(\boldsymbol{A},\boldsymbol{B})-(1-\widehat{\xi}^{[1]})\widehat{\xi}^{[2]}\varphi(\boldsymbol{B},\boldsymbol{A})-(1-\widehat{\xi}^{[1]})(1-\widehat{\xi}^{[2]})\varphi(\boldsymbol{B},\boldsymbol{B})$ in Eq. (S19), as mentioned in Ref. [1]. For any of the four absorbing states, $\varphi(\boldsymbol{\xi})-\widehat{\xi}^{[1]}\widehat{\xi}^{[2]}\varphi(\boldsymbol{A},\boldsymbol{A})-\widehat{\xi}^{[1]}(1-\widehat{\xi}^{[2]})\varphi(\boldsymbol{A},\boldsymbol{B})-(1-\widehat{\xi}^{[1]})\widehat{\xi}^{[2]}\varphi(\boldsymbol{B},\boldsymbol{A})-(1-\widehat{\xi}^{[1]})(1-\widehat{\xi}^{[2]})\varphi(\boldsymbol{B},\boldsymbol{B})$ is equal to 0, which is the requirement for using Lemma 1 in Ref. [3]. We also use the fact that $\mathbb{E}[1]=1$ to derive the last term on the right-hand side of Eq. (S19).

A.3 Condition for the cooperator and mutant to be selected in a general form

In this section, we derive recurrence relations for deducing conditions for selecting cooperation in the game layer (i.e., layer 1) and mutant in the constant-selection layer (i.e., layer 2). The payoff matrix of the donation game, which is used in layer 1, is given by

$$\begin{array}{cc} & \begin{array}{cc} \mathrm{C} & \mathrm{D} \end{array} \\ \begin{array}{c} \mathrm{C} \\ \mathrm{D} \end{array} & \begin{pmatrix} b-c & -c \\ b & 0 \end{pmatrix} \end{array}, \tag{S20}$$

where the entries of the matrix represent the payoff to the row player. Recall that $p_{ij} = \frac{w_{ij}}{s_i}$, where w_{ij} is the (i,j) entry of the adjacency matrix of the undirected network layer 1, and s_i is the degree of the ith node in layer 1. The payoff for the kth replica node in layer 1 is given by

$$u_k^{[1]}(\boldsymbol{x}) = \sum_{\ell=1}^{N} \left(-c \cdot p_{k\ell} \cdot x_k^{[1]} + b \cdot p_{k\ell} \cdot x_\ell^{[1]} \right) = \sum_{\ell=1}^{N} \left(-C_{k\ell} \cdot x_k^{[1]} + B_{\ell k} \cdot x_\ell^{[1]} \right), \tag{S21}$$

where $C_{kl} = c \cdot p_{kl}$ and $B_{lk} = b \cdot p_{k\ell}$. We write the payoff matrix in the case of constant selection as

$$\begin{array}{cc} & \begin{array}{cc} \mathrm{M} & \mathrm{R} \end{array} \\ \begin{array}{c} \mathrm{M} \\ \mathrm{R} \end{array} & \begin{pmatrix} r & r \\ 1 & 1 \end{pmatrix} \end{array}, \tag{S22}$$

where M and R stand for mutant and resident types, respectively. The payoff for the kth replica node in layer 2 is given by

$$u_k^{[2]}(\boldsymbol{x}) = x_k^{[2]}\,(r-1) + 1. \tag{S23}$$

The total payoff for the kth individual is given by

$$u_k(\boldsymbol{x}) = u_k^{[1]}(\boldsymbol{x}) + u_k^{[2]}(\boldsymbol{x}) = \sum_{\ell=1}^{N} \left(-C_{k\ell} \cdot x_k^{[1]} + B_{\ell k} \cdot x_\ell^{[1]} \right) + x_k^{[2]}\,(r-1) + 1. \tag{S24}$$

We define $\mathbf{F} = (F_1, \ldots, F_N)$, where we remind that F_i is the fecundity of the ith node. The marginal effect of the individual k's fitness on the probability that i replaces j in layer L [9] is denoted by

$$m_{k;ij}^{[L]} = \left. \frac{\partial e_{ij}^{[L]}}{\partial F_k} \right|_{\mathbf{F}=\mathbf{1}}, \tag{S25}$$

where $\mathbf{1} = (1, \ldots, 1)$. We find that

$$\left. \frac{\mathrm{d}}{\mathrm{d}\delta} e_{ij}^{[L]}(\boldsymbol{x}) \right|_{\delta=0} = \sum_{k=1}^{N} \left. \frac{\partial e_{ij}^{[L]}}{\partial F_k} \right|_{\mathbf{F}=1} \cdot \left. \frac{\mathrm{d}F_k}{\mathrm{d}\delta} \right|_{\delta=0} = \sum_{k=1}^{N} m_{k;ij}^{[L]} u_k(\boldsymbol{x}) = \sum_{k=1}^{N} m_{k;ij}^{[L]} \left[u_k^{[1]}(\boldsymbol{x}) + u_k^{[2]}(\boldsymbol{x}) \right]. \tag{S26}$$

Thus, it follows from the definition of $\widehat{\Delta}_{\mathrm{sel}}^{[L]}(\boldsymbol{x})$ (i.e., Eq. (S7)) that

$$\begin{aligned} \left. \frac{\mathrm{d}}{\mathrm{d}\delta} \widehat{\Delta}_{\mathrm{sel}}^{[L]}(\boldsymbol{x}) \right|_{\delta=0} &= \sum_{i,j,k=1}^{N} \pi_i^{[L]} m_{k;ji}^{[L]} \left(x_j^{[L]} - x_i^{[L]} \right) \left[u_k^{[1]}(\boldsymbol{x}) + u_k^{[2]}(\boldsymbol{x}) \right] \\ &= \sum_{i,j,k=1}^{N} \pi_i^{[L]} m_{k;ji}^{[L]} \left(x_j^{[L]} - x_i^{[L]} \right) \left\{ \left[\sum_{\ell=1}^{N} \left(-C_{k\ell} \cdot x_k^{[1]} + B_{\ell k} \cdot x_\ell^{[1]} \right) \right] + \left[x_k^{[2]}\,(r-1) + 1 \right] \right\} \\ &= \sum_{i,j,k=1}^{N} \pi_i^{[L]} m_{k;ji}^{[L]} \left\{ - \left[\sum_{\ell=1}^{N} \left(x_j^{[L]} x_k^{[1]} - x_i^{[L]} x_k^{[1]} \right) C_{k\ell} - \left(x_j^{[L]} x_\ell^{[1]} - x_i^{[L]} x_\ell^{[1]} \right) B_{\ell k} \right] \right. \\ &\quad \left. + \left[\left(x_j^{[L]} x_k^{[2]} - x_i^{[L]} x_k^{[2]} \right) (r-1) + \left(x_j^{[L]} - x_i^{[L]} \right) \right] \right\}. \end{aligned} \tag{S27}$$

We denote by $x_{ij}^{\boldsymbol{\xi}[L,L']} \equiv \mathbb{E}_{\mathrm{RMC}(\boldsymbol{\xi})}\left[x_i^{[L]} x_j^{[L']}\right]$ the probability that individual i in layer L and individual j in layer L' are both of type A in the neutral RMC distribution. By taking the expectation of both sides of Eq. (S27) and combining the result with Eq. (S16) and the definition of the RMC distribution, we obtain

$$\begin{aligned}\frac{\mathrm{d}}{\mathrm{d}\delta}\rho_{\boldsymbol{A}}^{[L]}(\boldsymbol{\xi})\bigg|_{\delta=0} =&\frac{1}{K^\circ}\mathbb{E}^\circ_{\mathrm{RMC}(\boldsymbol{\xi})}\left[\frac{\mathrm{d}}{\mathrm{d}\delta}\widehat{\Delta}_{\mathrm{sel}}^{[L]}\right]_{\delta=0} \\ =&\frac{1}{K^\circ}\sum_{i,j,k=1}^{N}\pi_i^{[L]} m_{k;ji}^{[L]}\left\{-\left[\sum_{\ell=1}^{N}\left(x_{jk}^{\boldsymbol{\xi}[L,1]}-x_{ik}^{\boldsymbol{\xi}[L,1]}\right)C_{k\ell}-\left(x_{j\ell}^{\boldsymbol{\xi}[L,1]}-x_{i\ell}^{\boldsymbol{\xi}[L,1]}\right)B_{\ell k}\right]\right. \\ &\left.+\left(x_{jk}^{\boldsymbol{\xi}[L,2]}-x_{ik}^{\boldsymbol{\xi}[L,2]}\right)(r-1)+\mathbb{E}_{\mathrm{RMC}(\boldsymbol{\xi})}\left[x_j^{[L]}-x_i^{[L]}\right]\right\}. \end{aligned} \tag{S28}$$

Let

$$\beta_{ij}^{\boldsymbol{\xi}[L]} := \frac{x_{ij}^{\boldsymbol{\xi}[LL]}}{K^\circ}, \quad L \in \{1,2\}. \tag{S29}$$

Owing to Lemma 1 in Ref. [1], $\beta_{ij}^{\boldsymbol{\xi}[1]}$ satisfies the following recurrence relation including when $i=j$:

$$\beta_{ij}^{\boldsymbol{\xi}[1]} = \xi_i^{[1]}\xi_j^{[1]} - \widehat{\xi}^{[1]} + \sum_{(R^{[1]},\alpha^{[1]})} p^\circ_{(R^{[1]},\alpha^{[1]})}\beta^{\boldsymbol{\xi}[1]}_{\tilde{\alpha}^{[1]}(i)\tilde{\alpha}^{[1]}(j)}. \tag{S30}$$

For the "cross terms" which are associated to the two layers jointly, we let

$$\gamma_{ij}^{\boldsymbol{\xi}[12]} := \frac{x_{ij}^{\boldsymbol{\xi}[12]}}{K^\circ} \tag{S31}$$

and

$$\gamma_{ij}^{\boldsymbol{\xi}[21]} := \frac{x_{ij}^{\boldsymbol{\xi}[21]}}{K^\circ}. \tag{S32}$$

Owing to Lemma 1 in Ref. [1], $\gamma_{ij}^{\boldsymbol{\xi}[12]}$ satisfies the recurrence relation given by

$$\gamma_{ij}^{\boldsymbol{\xi}[12]} = \xi_i^{[1]}\xi_j^{[2]} - \widehat{\xi}^{[1]}\widehat{\xi}^{[2]} + \sum_{(R^{[1]},\alpha^{[1]}),(R^{[2]},\alpha^{[2]})} p^\circ_{(R^{[1]},\alpha^{[1]})}p^\circ_{(R^{[2]},\alpha^{[2]})}\gamma^{\boldsymbol{\xi}[1,2]}_{\tilde{\alpha}^{[1]}(i)\tilde{\alpha}^{[2]}(j)} \tag{S33}$$

including when $i=j$. Equation (S33) implies that $\gamma_{ij}^{\boldsymbol{\xi}[12]} = \gamma_{ij}^{\boldsymbol{\xi}[21]}$. To compute $\mathbb{E}_{\mathrm{RMC}(\boldsymbol{\xi})}\left[x_j^{[L]}-x_i^{[L]}\right]$ on the right-hand side of Eq. (S28), we let

$$\eta_i^{\boldsymbol{\xi}[1]} := \mathbb{E}_{\mathrm{RMC}(\boldsymbol{\xi})}\left[\frac{x_i^{[1]}}{K^\circ}\right]. \tag{S34}$$

Owing to Lemma 1 in Ref. [1], $\eta_1^{\boldsymbol{\xi}[1]}, \ldots, \eta_N^{\boldsymbol{\xi}[1]}$ satisfy the recurrence relation given by

$$\eta_i^{\boldsymbol{\xi}[1]} = \xi_i^{[1]} - \widehat{\xi}^{[1]}\widehat{\xi}^{[2]} - \widehat{\xi}^{[1]}\left(1-\widehat{\xi}^{[2]}\right) + \sum_{(R^{[1]},\alpha^{[1]})} p^\circ_{(R^{[1]},\alpha^{[1]})}\eta^{\boldsymbol{\xi}[1]}_{\tilde{\alpha}^{[1]}(i)}. \tag{S35}$$

By setting $L=1$ in Eq. (S28) and using Eqs. (S29), (S31), and (S34), we obtain

$$\begin{aligned}\frac{\mathrm{d}}{\mathrm{d}\delta}\rho_{\boldsymbol{A}}^{[1]}(\boldsymbol{\xi})\bigg|_{\delta=0} = &\sum_{i,j,k=1}^{N}\pi_i^{[1]} m_{k;ji}^{[1]}\left\{-\left[\sum_{\ell=1}^{N}\left(\beta_{jk}^{\boldsymbol{\xi}[1]}-\beta_{ik}^{\boldsymbol{\xi}[1]}\right)C_{k\ell}-\left(\beta_{jl}^{\boldsymbol{\xi}[1]}-\beta_{il}^{\boldsymbol{\xi}[1]}\right)B_{\ell k}\right]\right. \\ &\left.+\left[\left(\gamma_{jk}^{\boldsymbol{\xi}[1,2]}-\gamma_{ik}^{\boldsymbol{\xi}[1,2]}\right)(r-1)+\left(\eta_j^{\boldsymbol{\xi}[1]}-\eta_i^{\boldsymbol{\xi}[1]}\right)\right]\right\}. \end{aligned} \tag{S36}$$

If $\beta_{ij}^{\boldsymbol{\xi}[1]}$ is a solution to Eq. (S30), then $\beta_{ij}^{\boldsymbol{\xi}[1]} + C'$ is also a solution to Eq. (S30) for any constant C'. Similarly, if $\gamma_{ij}^{\boldsymbol{\xi}[1,2]}$ is a solution to Eq. (S33), then $\gamma_{ij}^{\boldsymbol{\xi}[1,2]} + C''$ is also a solution to Eq. (S33) for any constant C''. The authors of Ref. [1] also pointed out that it does not matter which solution is used because only the differences of $\beta_{ij}^{\boldsymbol{\xi}[1]}$ and $\gamma_{ij}^{\boldsymbol{\xi}[1,2]}$ are used in Eq. (S36). By the fixation axiom in Refs. [1–3], the space of solutions is two-dimensional. In Ref. [1], they enforced two additional arbitrary constraints, which altogether ensure that the solution is unique. We impose these conditions, which are

$$\sum_{i=1}^{N} \pi_i^{[1]} \beta_{ii}^{\boldsymbol{\xi}[1]} = 0 \tag{S37}$$

and

$$\sum_{i=1}^{N} \pi_i^{[1]} \gamma_{ii}^{\boldsymbol{\xi}[1,2]} = 0. \tag{S38}$$

In our case, we have the term $\eta_j^{\boldsymbol{\xi}[1]} - \eta_i^{\boldsymbol{\xi}[1]}$, which is not present in Ref. [1]. Therefore, we also impose that $\sum_{i=1}^{N} \eta_i^{\boldsymbol{\xi}[1]} = 0$ to ensure uniqueness of the solution, which is again justified because only the pairwise difference $\eta_j^{\boldsymbol{\xi}[1]} - \eta_i^{\boldsymbol{\xi}[1]}$ matters.

Equation (S15) implies that selection favors cooperation in the limit of weak selection when starting from state $\boldsymbol{\xi}$ if and only if

$$\left. \frac{\mathrm{d}}{\mathrm{d}\delta} \rho_{\boldsymbol{A}}^{[1]}\left(\boldsymbol{\xi}\right) \right|_{\delta=0} > 0. \tag{S39}$$

By substituting Eq. (S36) in Eq. (S39), we obtain

$$\begin{aligned} \sum_{i,j,k=1}^{N} \pi_i^{[1]} m_{k,ji}^{[1]} \Bigg\{ - \left[\sum_{\ell=1}^{N} \left(\beta_{jk}^{\boldsymbol{\xi}[1]} - \beta_{ik}^{\boldsymbol{\xi}[1]} \right) C_{k\ell} - \left(\beta_{jl}^{\boldsymbol{\xi}[1]} - \beta_{il}^{\boldsymbol{\xi}[1]} \right) B_{\ell k} \right] \\ + \left[\left(\gamma_{jk}^{\boldsymbol{\xi}[1,2]} - \gamma_{ik}^{\boldsymbol{\xi}[1,2]} \right) (r-1) + \left(\eta_j^{\boldsymbol{\xi}[1]} - \eta_i^{\boldsymbol{\xi}[1]} \right) \right] \Bigg\} > 0. \end{aligned} \tag{S40}$$

Text B Evolution of the cooperator and mutant under the dB-dB rule

In this section, we derive the condition under which the cooperator is favored in layer 1 and that under which the mutant is favored in layer 2, assuming the dB rule in both layers, i.e., dB-dB rule. The derivation amounts to calculating individual quantities in Eq. (S40) and simplifying the expressions to facilitate interpretation and numerical computations.

B.1 Preliminaries

For the dB rule, we obtain

$$e_{ji}^{[1]} = \frac{1}{N} \frac{w_{ij}^{[1]} F_i(x)}{\sum_{\ell=1}^{N} w_{\ell i} F_\ell(x)}. \tag{S41}$$

By substituting Eq. (S41) in Eq. (S25), we obtain

$$\begin{aligned} m_{k;ji} = \left.\frac{\partial e_{ji}}{\partial F_k}\right|_{\mathbf{F}=\mathbf{1}} &= \left.\frac{\partial}{\partial F_k} \frac{1}{N} \frac{w_{ji} F_j(x)}{\sum_{\ell=1}^{N} w_{\ell i} F_\ell(x)}\right|_{\mathbf{F}=\mathbf{1}} \\ &= \begin{cases} -\frac{1}{N} w_{ji} F_j(x) \left.\frac{w_{ki}}{\left[\sum_{\ell=1}^{N} w_{\ell i} F_\ell(x)\right]^2}\right|_{\mathbf{F}=\mathbf{1}} & \text{if } j \neq k \\ \frac{1}{N} w_{ki} \left.\frac{\left[\sum_{\ell=1}^{N} w_{\ell i} F_\ell(x)\right] - F_k(x) w_{ki}}{\left[\sum_{\ell=1}^{N} w_{\ell i} F_\ell(x)\right]^2}\right|_{\mathbf{F}=\mathbf{1}} & \text{if } j = k \end{cases} \\ &= \begin{cases} -\frac{1}{N} w_{ji} \frac{w_{ki}}{\left(\sum_{\ell=1}^{N} w_{\ell i}\right)^2} & \text{if } j \neq k \\ \frac{1}{N} w_{ki} \frac{\left(\sum_{\ell=1}^{N} w_{\ell i}\right) - w_{ki}}{\left[\sum_{\ell=1}^{N} w_{\ell i} F_\ell(x)\right]^2} & \text{if } j = k \end{cases} \\ &= \begin{cases} -\frac{1}{N} p_{ij} p_{ik} & \text{if } j \neq k \\ \frac{1}{N} p_{ij} (1 - p_{ik}) & \text{if } j = k \end{cases} \\ &= \frac{1}{N} p_{ij} \left(\delta_{j,k} - p_{ik}\right). \end{aligned} \tag{S42}$$

We have dropped the superscript $[L]$ for $m_{k;ji}^{[L]}$ and $w_{ij}^{[L]}$ in Eq. (S42) because the calculations are identical for any L.

B.2 Conditions for the cooperator to be selected

To compute the condition under which the cooperator is favored, Eq. (S40), we introduce a short-hand notation

$$f_{ijk} \equiv -\beta_{ij}^{\boldsymbol{\xi}[1]} C_{jk} + \beta_{ik}^{\boldsymbol{\xi}[1]} B_{kj} + \gamma_{ij}^{\boldsymbol{\xi}[1,2]} (r-1) + \eta_i^{\boldsymbol{\xi}[1]}. \tag{S43}$$

By substituting Eq. (S43) in Eq. (S36), we obtain

$$
\begin{aligned}
\frac{\mathrm{d}}{\mathrm{d}\delta}\rho^{[1]}_{\boldsymbol{A}}\left(\boldsymbol{\xi}\right)\bigg|_{\delta=0} &= \sum_{i,j,k,\ell=1}^{N} \pi_i^{[1]} m^{[1]}_{k;ji}(f_{jkl}-f_{ikl}) \\
&= \sum_{i,j,k,\ell=1}^{N} \pi_i^{[1]} \frac{1}{N} p^{[1]}_{ij}(\delta_{j,k}-p^{[1]}_{ik})(f_{jkl}-f_{ikl}) \\
&= \frac{1}{N}\sum_{i,j,k,\ell=1}^{N} \pi_i^{[1]} p^{[1]}_{ij}\delta_{j,k} f_{jkl} - \frac{1}{N}\sum_{i,j,k,\ell=1}^{N} \pi_i^{[1]} p^{[1]}_{ij} p^{[1]}_{ik} f_{jkl} \\
&\quad - \frac{1}{N}\sum_{i,j,k,\ell=1}^{N} \pi_i^{[1]} p^{[1]}_{ij}\delta_{j,k} f_{ikl} + \frac{1}{N}\sum_{i,j,k,\ell=1}^{N} \pi_i^{[1]} p^{[1]}_{ij} p^{[1]}_{ik} f_{ikl} \\
&= \frac{1}{N}\sum_{i,j,\ell=1}^{N} \pi_i^{[1]} p^{[1]}_{ij} f_{jjl} - \frac{1}{N}\sum_{i,j,k,\ell=1}^{N} \pi_i^{[1]} p^{[1]}_{ji} p^{[1]}_{ik} f_{jkl} \\
&\quad - \frac{1}{N}\sum_{i,j,\ell=1}^{N} \pi_i^{[1]} p^{[1]}_{ij} f_{ijl} + \frac{1}{N}\sum_{i,k,\ell=1}^{N} \pi_i^{[1]} \left(\sum_{j=1}^{N} p^{[1]}_{ij}\right) p^{[1]}_{ik} f_{ikl} \\
&= \frac{1}{N}\sum_{j,\ell=1}^{N} \pi_j^{[1]} \left(\sum_{i=1}^{N} p^{[1]}_{ji}\right) f_{jjl} - \frac{1}{N}\sum_{j,k,\ell=1}^{N} \pi_j^{[1]} \left(p^{[1]}\right)^{(2)}_{jk} f_{jkl} \\
&\quad \cancel{- \frac{1}{N}\sum_{i,j,\ell=1}^{N} \pi_i^{[1]} p^{[1]}_{ij} f_{ijl}} + \cancel{\frac{1}{N}\sum_{i,k,\ell=1}^{N} \pi_i^{[1]} p^{[1]}_{ik} f_{ikl}} \\
&= \frac{1}{N}\sum_{j,\ell=1}^{N} \pi_j^{[1]} f_{jjl} - \frac{1}{N}\sum_{i,j,\ell=1}^{N} \pi_i^{[1]} \left(p^{[1]}\right)^{(2)}_{ij} f_{ijl} \\
&= \frac{1}{N}\sum_{i,\ell=1}^{N} \pi_i^{[1]} \left[-\beta^{\boldsymbol{\xi}[1]}_{ii} C_{il} + \beta^{\boldsymbol{\xi}[1]}_{il} B_{li} + \gamma^{\boldsymbol{\xi}[1,2]}_{ii}(r-1) + \eta^{\boldsymbol{\xi}[1]}_{i}\right] \\
&\quad - \frac{1}{N}\sum_{i,j,\ell=1}^{N} \pi_i^{[1]} \left(p^{[1]}\right)^{(2)}_{ij} \left[-\beta^{\boldsymbol{\xi}[1]}_{ij} C_{jl} + \beta^{\boldsymbol{\xi}[1]}_{il} B_{lj} + \gamma^{\boldsymbol{\xi}[1,2]}_{ij}(r-1) + \eta^{\boldsymbol{\xi}[1]}_{i}\right],
\end{aligned} \tag{S44}
$$

where $\left(p^{[1]}\right)^{(n)}_{ij}$ is the probability that the random walker in layer 1 moves from replica node i to replica node j in n steps. To derive the first equality in Eq. (S44), we used Eq. (S42). To derive the third equality in Eq. (S44), we used $\pi_i^{[1]} p^{[1]}_{ij} = \pi_j^{[1]} p^{[1]}_{ji}$, $\forall\ i,\ j$. Equation (S44) implies that $\frac{\mathrm{d}}{\mathrm{d}\delta}\rho^{[1]}_{\boldsymbol{A}}\left(\boldsymbol{\xi}\right)\Big|_{\delta=0} > 0$ holds true if and only if

$$
\begin{aligned}
&\sum_{i=1}^{N} \pi_i^{[1]} \sum_{\ell=1}^{N} \left[-\beta^{\boldsymbol{\xi}[1]}_{ii} C_{il} + \beta^{\boldsymbol{\xi}[1]}_{il} B_{li} + \gamma^{\boldsymbol{\xi}[1,2]}_{ii}\left(r-1\right) + \eta^{\boldsymbol{\xi}[1]}_{i}\right] > \\
&\sum_{i,j=1}^{N} \pi_i^{[1]} \left(p^{[1]}\right)^{(2)}_{ij} \sum_{\ell=1}^{N} \left[-\beta^{\boldsymbol{\xi}[1]}_{ij} C_{jl} + \beta^{\boldsymbol{\xi}[1]}_{il} B_{lj} + \gamma^{\boldsymbol{\xi}[1,2]}_{ij}\left(r-1\right) + \eta^{\boldsymbol{\xi}[1]}_{i}\right].
\end{aligned} \tag{S45}
$$

By substituting $C_{kl} = c \cdot p_{kl}^{[1]}$ and $B_{lk} = b \cdot p_{lk}^{[1]}$ in Eq. (S45), we obtain

$$\sum_{i=1}^{N} \pi_i^{[1]} \sum_{\ell=1}^{N} \left[-\beta_{ii}^{\boldsymbol{\xi}[1]} c \cdot p_{il}^{[1]} + \beta_{il}^{\boldsymbol{\xi}[1]} b \cdot p_{il}^{[1]} + \gamma_{ii}^{\boldsymbol{\xi}[1,2]} (r-1) + \eta_i^{\boldsymbol{\xi}[1]} \right] >$$
$$\sum_{i,j=1}^{N} \pi_i^{[1]} \left(p^{[1]} \right)_{ij}^{(2)} \sum_{\ell=1}^{N} \left[-\beta_{ij}^{\boldsymbol{\xi}[1]} c \cdot p_{jl}^{[1]} + \beta_{il}^{\boldsymbol{\xi}[1]} b \cdot p_{jl}^{[1]} + \gamma_{ij}^{\boldsymbol{\xi}[1,2]} (r-1) + \eta_i^{\boldsymbol{\xi}[1]} \right]. \tag{S46}$$

To simplify Eq. (S46), we define

$$\theta_n^{\boldsymbol{\xi}[1]} := \sum_{i,j=1}^{N} \pi_i^{[1]} \left(p^{[1]} \right)_{ij}^{(n)} \beta_{ij}^{\boldsymbol{\xi}[1]} \tag{S47}$$

and

$$\phi_{n,m}^{\boldsymbol{\xi}[1,2]} := \sum_{i,j=1}^{N} \pi_i^{[1]} \left(p^{[1,2]} \right)_{ij}^{(n,m)} \gamma_{ij}^{\boldsymbol{\xi}[1,2]}, \tag{S48}$$

where $\left(p^{[1,2]}\right)_{ij}^{(n,m)}$ is the probability that the random walker moves from replica node i to replica node j when the first n steps of the random walk occur in layer 1 and the subsequent m steps occur in layer 2. By substituting Eqs. (S47) and (S48) in Eq. (S46) and using the fact that

$$\sum_{i,j=1}^{N} \pi_i^{[1]} \left(p^{[1]} \right)_{ij}^{(n)} \eta_i^{\boldsymbol{\xi}[1]} = \sum_{i=1}^{N} \pi_i^{[1]} \eta_i^{\boldsymbol{\xi}[1]} \sum_{j=1}^{N} \left(p^{[1]} \right)_{ij}^{(n)} = \sum_{i=1}^{N} \pi_i^{[1]} \eta_i^{\boldsymbol{\xi}[1]} = 0 \tag{S49}$$

for any n, we obtain

$$c\theta_2^{\boldsymbol{\xi}[1]} + b \left(\theta_1^{\boldsymbol{\xi}[1]} - \theta_3^{\boldsymbol{\xi}[1]} \right) - (r-1)\, \phi_{2,0}^{\boldsymbol{\xi}[1,2]} > 0, \tag{S50}$$

which is Eq. (3) in the main text. It should be noted that $\eta_i^{\boldsymbol{\xi}[1]}$ in Eq. (S46) has been canceled out.

Next, we solve the recurrence equations Eqs. (S30), (S33), and (S35) to determine $\theta_n^{\boldsymbol{\xi}[1]}$, $n \in \{1,2,3\}$ and $\phi_{n,m}^{\boldsymbol{\xi}[1,2]}$ through Eq. (S47) and (S48), respectively. The rest of the derivation is identical to section 2.1.1 of the ESM for Ref. [1] because these recurrences were derived in Ref. [1] for any dynamics using the dB rule, except Eq. (S57). Therefore, we omit some details. Note that Eq. (S57) contains the recurrence for the η term, which is absent in their case. For $i = j$, Eq. (S30) reduces to

$$\beta_{ii}^{\boldsymbol{\xi}[1]} = \xi_i^{[1]} - \widehat{\xi}^{[1]} + \frac{1}{N} \sum_{k=1}^{N} p_{ik}^{[1]} \beta_{kk}^{\boldsymbol{\xi}[1]} + \left(1 - \frac{1}{N} \right) \beta_{ii}^{\boldsymbol{\xi}[1]}, \tag{S51}$$

which gives

$$\beta_{ii}^{\boldsymbol{\xi}[1]} = N \left(\xi_i^{[1]} - \widehat{\xi}^{[1]} \right) + \sum_{k=1}^{N} p_{ik}^{[1]} \beta_{kk}^{\boldsymbol{\xi}[1]}. \tag{S52}$$

For $i \neq j$, we obtain

$$\beta_{ij}^{\boldsymbol{\xi}[1]} = \xi_i^{[1]} \xi_j^{[1]} - \widehat{\xi}^{[1]} + \frac{1}{N} \sum_{k=1}^{N} p_{ik}^{[1]} \beta_{kj}^{\boldsymbol{\xi}[1]} + \frac{1}{N} \sum_{k=1}^{N} p_{jk}^{[1]} \beta_{ik}^{\boldsymbol{\xi}[1]} + \left(1 - \frac{2}{N} \right) \beta_{ij}^{\boldsymbol{\xi}[1]}, \tag{S53}$$

which gives

$$\beta_{ij}^{\boldsymbol{\xi}[1]} = \frac{N}{2} \left(\xi_i^{[1]} \xi_j^{[1]} - \widehat{\xi}^{[1]} \right) + \frac{1}{2} \sum_{k=1}^{N} p_{ik}^{[1]} \beta_{kj}^{\boldsymbol{\xi}[1]} + \frac{1}{2} \sum_{k=1}^{N} p_{jk}^{[1]} \beta_{ik}^{\boldsymbol{\xi}[1]}. \tag{S54}$$

For $i, j \in \{1, \ldots, N\}$, Eq. (S33) reduces to

$$\begin{aligned}\gamma_{ij}^{\boldsymbol{\xi}[1,2]} = {} & \xi_i^{[1]}\xi_j^{[2]} - \widehat{\xi}^{[1]}\widehat{\xi}^{[2]} + \frac{1}{N^2}\sum_{k_1,k_2=1}^{N} p_{ik_1}^{[1]} p_{jk_2}^{[2]} \gamma_{k_1k_2}^{\boldsymbol{\xi}[1,2]} \\ & + \frac{1}{N}\left(1 - \frac{1}{N}\right)\sum_{k_1=1}^{N} p_{ik_1}^{[1]}\gamma_{k_1j}^{\boldsymbol{\xi}[1,2]} + \frac{1}{N}\left(1 - \frac{1}{N}\right)\sum_{k_2=1}^{N} p_{jk_2}^{[2]}\gamma_{ik_2}^{\boldsymbol{\xi}[1,2]} \\ & + \left(1 - \frac{1}{N}\right)^2 \gamma_{ij}^{\boldsymbol{\xi}[1,2]}, \end{aligned} \tag{S55}$$

which gives

$$\begin{aligned}\gamma_{ij}^{\boldsymbol{\xi}[1,2]} = {} & \frac{N^2}{2N-1}\left(\xi_i^{[1]}\xi_j^{[2]} - \widehat{\xi}^{[1]}\widehat{\xi}^{[2]}\right) + \frac{1}{2N-1}\sum_{k_1,k_2=1}^{N} p_{ik_1}^{[1]} p_{jk_2}^{[2]} \gamma_{k_1k_2}^{\boldsymbol{\xi}[1,2]} \\ & + \frac{N-1}{2N-1}\sum_{k_1=1}^{N} p_{ik_1}^{[1]}\gamma_{k_1j}^{\boldsymbol{\xi}[1,2]} + \frac{N-1}{2N-1}\sum_{k_2=1}^{N} p_{jk_2}^{[2]}\gamma_{ik_2}^{\boldsymbol{\xi}[1,2]}. \end{aligned} \tag{S56}$$

For $i \in \{1, \ldots, N\}$, Eq. (S35) reduces to

$$\eta_i^{\boldsymbol{\xi}[1]} = \xi_i^{[1]} - \widehat{\xi}^{[1]}\widehat{\xi}^{[2]} - \widehat{\xi}^{[1]}\left(1 - \widehat{\xi}^{[2]}\right) + \frac{1}{N}\sum_{k=1}^{N} p_{ik}^{[1]}\eta_k^{\boldsymbol{\xi}[1]} + \left(1 - \frac{1}{N}\right)\eta_i^{\boldsymbol{\xi}[1]}, \tag{S57}$$

which gives

$$\eta_i^{\boldsymbol{\xi}[1]} = N\left[\xi_i^{[1]} - \widehat{\xi}^{[1]}\widehat{\xi}^{[2]} - \widehat{\xi}^{[1]}\left(1 - \widehat{\xi}^{[2]}\right)\right] + \sum_{k=1}^{N} p_{ik}^{[1]}\eta_k^{\boldsymbol{\xi}[1]}. \tag{S58}$$

By solving the recurrences given by Eqs. (S52), (S54), (S56), and (S58), and substituting the solutions in Eqs. (S47) and (S48) and the obtained $\theta_n^{\boldsymbol{\xi}[1]}$ and $\phi_{n,m}^{\boldsymbol{\xi}[1,2]}$ in Eq. (S50), we obtain the conditions under which selection favors cooperation.

B.3 Conditions for the mutant to be selected

To avoid complication of the equations, in this section, we swap the two layers such that constant selection occurs in layer 1 and the donation game occurs in layer 2. Then, Eq. (S36) becomes

$$\begin{aligned}\left.\frac{\mathrm{d}}{\mathrm{d}\delta}\rho_{\boldsymbol{A}}^{[1]}(\boldsymbol{\xi})\right|_{\delta=0} = {} & \sum_{i,j,k=1}^{N} \frac{1}{N}\pi_i^{[1]} m_{k;ji}^{[1]} \Bigg\{\left[\left(\beta_{jk}^{\boldsymbol{\xi}[1]} - \beta_{ik}^{\boldsymbol{\xi}[1]}\right)(r-1) + \left(\eta_j^{\boldsymbol{\xi}[1]} - \eta_i^{\boldsymbol{\xi}[1]}\right)\right] \\ & + \sum_{\ell=1}^{N}\left[-\left(\gamma_{jk}^{\boldsymbol{\xi}[1,2]} - \gamma_{ik}^{\boldsymbol{\xi}[1,2]}\right)C_{k\ell} + \left(\gamma_{j\ell}^{\boldsymbol{\xi}[1,2]} - \gamma_{i\ell}^{\boldsymbol{\xi}[1,2]}\right)B_{\ell k}\right]\Bigg\}. \end{aligned} \tag{S59}$$

Similarly, Eq. (S46) becomes

$$\begin{aligned} & \sum_{i=1}^{N} \pi_i^{[1]} \sum_{\ell=1}^{N}\left[\beta_{ii}^{\boldsymbol{\xi}[1]}(r-1) + \eta_i^{\boldsymbol{\xi}[1]} - \gamma_{ii}^{\boldsymbol{\xi}[1,2]} c p_{k\ell}^{[2]} + \gamma_{i\ell}^{\boldsymbol{\xi}[1,2]} b p_{i\ell}^{[2]}\right] > \\ & \sum_{i,j=1}^{N} \pi_i^{[1]} \left(p^{[1]}\right)_{ij}^{(2)} \sum_{\ell=1}^{N}\left[\beta_{ij}^{\boldsymbol{\xi}[1]}(r-1) + \eta_i^{\boldsymbol{\xi}[1]} - \gamma_{ij}^{\boldsymbol{\xi}[1,2]} c p_{j\ell}^{[2]} + \gamma_{i\ell}^{\boldsymbol{\xi}[1,2]} b p_{j\ell}^{[2]}\right]. \end{aligned} \tag{S60}$$

By carrying out calculations similar to those in Ref. [1], we find that Equation (S60) simplifies to

$$- (r-1)\, \theta_2^{\boldsymbol{\xi}[1]} + c\phi_{2,0}^{\boldsymbol{\xi}[1,2]} + b\left(\phi_{0,1}^{\boldsymbol{\xi}[1,2]} - \phi_{2,1}^{\boldsymbol{\xi}[1,2]}\right) > 0. \tag{S61}$$

Equation (S61) is equivalent to Eq. (4) in the main text; note that Eq. (4) is shown in terms of the original layer convention (i.e., the donation game occurs in layer 1, and the constant-selection dynamics occurs in layer 2).

Text C Evolution of cooperator and mutant under the dB-Bd rule

In this section, we derive the conditions for the cooperation and mutant type to be favored when updating occurs according to the death-Birth rule in the game layer (i.e., layer 1) and the Birth-death rule in constant-selection layer (i.e., layer 2), i.e., under the dB-Bd rule.

C.1 Preliminaries

For the Bd rule, we obtain

$$e_{ji}^{[2]} = \frac{w_{ji}}{\sum_{\ell=1}^{N} w_{j\ell}^{[2]}} \cdot \frac{F_j(x)}{\sum_{\ell=1}^{N} F_\ell(x)} \tag{S62}$$

for layer 2. By substituting Eq. (S62) in Eq. (S25), we obtain

$$\begin{aligned} m_{k;ji}^{[2]} = \left.\frac{\partial e_{ji}}{\partial F_k}\right|_{\mathbf{F}=\mathbf{1}} &= \frac{w_{ji}^{[2]}}{\sum_{\ell=1}^{N} w_{j\ell}^{[2]}} \left.\frac{\partial}{\partial F_k} \frac{F_j(x)}{\sum_{\ell=1}^{N} F_\ell(x)}\right|_{\mathbf{F}=\mathbf{1}} \\ &= \begin{cases} p_{ji}^{[2]} F_j(x) \left.\frac{-1}{\left[\sum_{\ell=1}^{N} F_\ell(x)\right]^2}\right|_{\mathbf{F}=\mathbf{1}} & \text{if } j \neq k \\ p_{ji}^{[2]} \left.\frac{\left[\sum_{\ell=1}^{N} F_\ell(x)\right] - F_k(x)}{\left[\sum_{\ell=1}^{N} w_{\ell i} F_\ell(x)\right]^2}\right|_{\mathbf{F}=\mathbf{1}} & \text{if } j = k \end{cases} \\ &= \begin{cases} -\frac{1}{N} p_{ji}^{[2]} & \text{if } j \neq k \\ \frac{N-1}{N^2} p_{ji}^{[2]} & \text{if } j = k \end{cases} \\ &= \frac{1}{N}\left(\delta_{j,k} - \frac{1}{N}\right) p_{ji}^{[2]}. \end{aligned} \tag{S63}$$

C.2 Conditions for the cooperator to be selected

Because layer 1 uses the dB rule, the condition under which the cooperator is selected remains in the same form, i.e., Eq. (S50). Furthermore, $\theta_n^{\boldsymbol{\xi}[1]}$, $n \in \{1,2,3\}$ remains the same as that under the dB-dB rule. This is because, in Eq. (S47), $\left(p^{[1]}\right)_{ij}^{(n)}$ only depends on the network structure in layer 1, $\pi_i^{[1]}$ depends on the network structure in layer 1 and the updating rule used in layer 1, and $\beta_{ij}^{\boldsymbol{\xi}[1]}$ only depends on the network structure and updating rule in layer 1. For obtaining $\beta_{ij}^{\boldsymbol{\xi}[1]}$, the recurrence equations to be used are Eqs. (S52) and (S54).

In contrast, the value of $\phi_{2,0}^{\boldsymbol{\xi}[1,2]}$ changes from the case of the dB-dB rule because $\gamma_{ij}^{\boldsymbol{\xi}[1,2]}$ in Eq. (S48) depends on the updating rule in layer 2 as well as the network structure of both layers. It should be noted that $\left(p^{[1,2]}\right)_{ij}^{(n,m)}$ still only depends on the network structure in both layers and hence is not influenced by the change in the updating rule in layer 2. We derive $\gamma_{ij}^{\boldsymbol{\xi}[1,2]}$ in section C.3 (see the self-consistent equation (S73) for $\gamma_{ij}^{\boldsymbol{\xi}[1,2]}$; note that $\gamma_{ij}^{\boldsymbol{\xi}[1,2]} = \gamma_{ij}^{\boldsymbol{\xi}[2,1]}$).

C.3 Conditions for the mutant to be selected

Similarly to Eq. (S43), in terms of the original layer-label convention (i.e., the donation game occurs in layer 1, and the constant-selection dynamics occurs in layer 2), we set

$$f_{ijk} \equiv -\gamma_{ij}^{\boldsymbol{\xi}[2,1]} C_{jk} + \gamma_{ik}^{\boldsymbol{\xi}[2,1]} B_{kj} + \beta_{ij}^{\boldsymbol{\xi}[2]}(r-1) + \eta_i^{\boldsymbol{\xi}[2]}. \tag{S64}$$

Note that the reproductive value under the Bd rule, which is assumed for layer 2 here, is given by [9–11]

$$\pi_i^{[2]} = \frac{\left(s_i^{[2]}\right)^{-1}}{\sum_{\ell=1}^{N}\left(s_\ell^{[2]}\right)^{-1}}. \tag{S65}$$

Therefore, we obtain

$$\begin{aligned}
\left.\frac{\mathrm{d}}{\mathrm{d}\delta}\rho_{\boldsymbol{A}}^{[2]}(\boldsymbol{\xi})\right|_{\delta=0} &= \sum_{i,j,k,\ell=1}^{N} \pi_i^{[2]} m_{k;ji}^{[2]} \left(f_{jk\ell} - f_{ik\ell}\right) \\
&= \frac{1}{N}\sum_{i,j=1}^{N}\pi_i^{[2]} p_{ji}^{[2]} \sum_{\ell=1}^{N}\left[-\gamma_{ii}^{\boldsymbol{\xi}[2,1]} C_{i\ell} + \gamma_{i\ell}^{\boldsymbol{\xi}[2,1]} B_{\ell i} + \beta_{ii}^{\boldsymbol{\xi}[2]}(r-1) + \eta_i^{\boldsymbol{\xi}[2]}\right] \\
&\quad - \frac{1}{N}\sum_{i,j=1}^{N}\pi_i^{[2]} p_{ji}^{[2]} \sum_{\ell=1}^{N}\left[-\gamma_{ik}^{\boldsymbol{\xi}[2,1]} C_{k\ell} + \gamma_{i\ell}^{\boldsymbol{\xi}[2,1]} B_{\ell j} + \beta_{ik}^{\boldsymbol{\xi}[2]}(r-1) + \eta_i^{\boldsymbol{\xi}[2]}\right].
\end{aligned} \tag{S66}$$

For detailed derivation of Eq. (S66), see Eq. (SI.60) in Ref. [1]. Equation (S66) implies that $\left.\frac{\mathrm{d}}{\mathrm{d}\delta}\rho_{\boldsymbol{A}}^{[2]}(\boldsymbol{\xi})\right|_{\delta=0} > 0$ if and only if

$$\begin{aligned}
&\sum_{i,j=1}^{N}\pi_i^{[2]} p_{ji}^{[2]} \sum_{\ell=1}^{N}\left[-\gamma_{ii}^{\boldsymbol{\xi}[2,1]} c p_{i\ell}^{[1]} + \gamma_{i\ell}^{\boldsymbol{\xi}[2,1]} b p_{i\ell}^{[1]} + \beta_{ii}^{\boldsymbol{\xi}[2]}(r-1) + \eta_i^{\boldsymbol{\xi}[2]}\right] > \\
&\sum_{i,j=1}^{N}\pi_i^{[2]} p_{ji}^{[2]} \sum_{\ell=1}^{N}\left[-\gamma_{ij}^{\boldsymbol{\xi}[2,1]} c p_{j\ell}^{[1]} + \gamma_{i\ell}^{\boldsymbol{\xi}[2,1]} b p_{j\ell}^{[1]} + \beta_{ij}^{\boldsymbol{\xi}[2]}(r-1) + \eta_i^{\boldsymbol{\xi}[2]}\right].
\end{aligned} \tag{S67}$$

In Eq. (S67), the $\eta_i^{\boldsymbol{\xi}[2]}$ terms cancel out, leading to

$$\begin{aligned}
&\sum_{i,j=1}^{N}\pi_i^{[2]} p_{ji}^{[2]} \sum_{\ell=1}^{N}\left[-\gamma_{ii}^{\boldsymbol{\xi}[2,1]} c p_{i\ell}^{[1]} + \gamma_{i\ell}^{\boldsymbol{\xi}[2,1]} b p_{i\ell}^{[1]} + \beta_{ii}^{\boldsymbol{\xi}[2]}(r-1)\right] > \\
&\sum_{i,j=1}^{N}\pi_i^{[2]} p_{ji}^{[2]} \sum_{\ell=1}^{N}\left[-\gamma_{ij}^{\boldsymbol{\xi}[2,1]} c p_{j\ell}^{[1]} + \gamma_{i\ell}^{\boldsymbol{\xi}[2,1]} b p_{j\ell}^{[1]} + \beta_{ij}^{\boldsymbol{\xi}[2]}(r-1)\right].
\end{aligned} \tag{S68}$$

To compute Eq. (S68), we follow Ref. [1] to obtain $\beta_{ij}^{\boldsymbol{\xi}[2]}$ and $\gamma_{ij}^{\boldsymbol{\xi}[21]}$ as solutions of recursive equations as follows.

For $i = j$, we obtain

$$\begin{aligned}
\beta_{ii}^{\boldsymbol{\xi}[2]} &= \xi_i^{[2]} - \widehat{\xi}^{[2]} + \sum_{\left(R^{[1]},\alpha^{[1]}\right)} p^{\circ}_{\left(R^{[2]},\alpha^{[2]}\right)} \beta_{\widetilde{\alpha}^{[2]}(i),\widetilde{\alpha}^{[2]}(i)}^{\boldsymbol{\xi}[2]} \\
&= \xi_i^{[2]} - \widehat{\xi}^{[2]} + \frac{1}{N}\sum_{k=1}^{N} p_{ki}^{[2]}\beta_{kk}^{\boldsymbol{\xi}[2]} + \left(1 - \frac{1}{N}\sum_{k=1}^{N} p_{ki}^{[2]}\right)\beta_{ii}^{\boldsymbol{\xi}[2]},
\end{aligned} \tag{S69}$$

which gives

$$\beta_{ii}^{\boldsymbol{\xi}[2]} = \frac{N\left(\xi_i^{[2]} - \widehat{\xi}_i^{[2]}\right) + \sum_{k=1}^{N} p_{ki}^{[2]}\beta_{kk}^{\boldsymbol{\xi}[2]}}{\sum_{k=1}^{N} p_{ki}^{[2]}}. \tag{S70}$$

For $i \neq j$, we obtain

$$\beta_{ij}^{\boldsymbol{\xi}[2]} = \frac{N\left(\xi_i^{[2]}\xi_j^{[2]} - \widehat{\xi}_i^{[2]}\right) + \sum_{k=1}^{N} p_{ki}^{[2]}\beta_{kj}^{\boldsymbol{\xi}[2]} + \sum_{k=1}^{N} p_{kj}^{[2]}\beta_{ik}^{\boldsymbol{\xi}[2]}}{\sum_{k=1}^{N} p_{ki}^{[2]} + \sum_{k=1}^{N} p_{kj}^{[2]}}. \tag{S71}$$

Finally, we obtain

$$\begin{aligned}
\gamma_{ij}^{\boldsymbol{\xi}[2,1]} &= \xi_i^{[1]}\xi_j^{[2]} - \widehat{\xi}^{[1]}\widehat{\xi}^{[2]} + \sum_{\substack{(R^{[1]},\alpha^{[1]}),\\(R^{[2]},\alpha^{[2]})}} p^{\boldsymbol{\xi}[1]}_{\left(R^{[1]},\alpha^{[1]}\right)} p^{\boldsymbol{\xi}[2]}_{\left(R^{[2]},\alpha^{[2]}\right)} \gamma^{\boldsymbol{\xi}[2,1]}_{\widetilde{\alpha}^{[1]}(i),\widetilde{\alpha}^{[2]}(j)} \\
&= \xi_i^{[1]}\xi_j^{[2]} - \widehat{\xi}^{[1]}\widehat{\xi}^{[2]} + \frac{1}{N^2}\sum_{k_1,k_2=1}^{N} p_{ik_1}^{[1]} p_{k_2 j}^{[2]} \gamma_{k_1 k_2}^{\boldsymbol{\xi}[2,1]} \\
&\quad + \frac{1}{N}\left(1 - \frac{1}{N}\right)\sum_{k_1=1}^{N} p_{ik_1}^{[1]}\gamma_{k_1 j}^{\boldsymbol{\xi}[2,1]} \\
&\quad + \frac{1}{N}\left(1 - \frac{1}{N}\sum_{k_2=1}^{N} p_{k_2 j}^{[2]}\right)\sum_{k_2=1}^{N} p_{k_2 j}^{[2]}\gamma_{ik_2}^{\boldsymbol{\xi}[2,1]} \\
&\quad + \left(1 - \frac{1}{N}\right)\left(1 - \frac{1}{N}\sum_{k_2=1}^{N} p_{k_2 j}^{[2]}\right)\gamma_{ij}^{\boldsymbol{\xi}[2,1]},
\end{aligned} \tag{S72}$$

which gives

$$\gamma_{ij}^{\xi[21]} = \frac{N^2\left(\xi_i^{[1]}\xi_j^{[2]} - \xi_i^{[1]}\xi_j^{[2]}\right) + \sum_{k_1,k_2=1}^{N} p_{ik_1}^{[1]} p_{k_2 j}^{[2]}\gamma_{k_1 k_2}^{\boldsymbol{\xi}[2,1]} + \left(N - \sum_{k_2=1}^{N} p_{k_2 j}^{[2]}\right)\sum_{k_1=1}^{N} p_{ik_1}^{[1]}\gamma_{k_1 j}^{\boldsymbol{\xi}[2,1]} + (N-1)\sum_{k_2=1}^{N} p_{k_2 j}^{[2]}\gamma_{ik_2}^{\boldsymbol{\xi}[2,1]}}{N + N\sum_{k_2=1}^{N} p_{k_2 j}^{[2]} - \sum_{k_2=1}^{N} p_{k_2 j}^{[2]}}. \tag{S73}$$

Text D Interpretation of Eqs. (3) and (4)

Our main theoretical results, Eqs. (3) and (4) in the main text, which are Eqs. (S50) and (S61), are an outcome of detailed mathematical analysis (see Text B). In this section, we provide a more intuitive explanation of Eqs. (3) and (4). A similar explanation is provided in Ref. [1] for two-layer networks in both of which the donation game is played (Fig. 2 and the associated text in Ref. [1]).

Following Ref. [1], consider an individual i that is chosen to update the strategy in layer 1. Cooperation is favored in layer 1 if a cooperating neighbor of i, denoted by j, has a larger expected payoff than a uniformly randomly chosen neighbor of i in layer 1, denoted by ℓ. Individual j receives an average benefit $b\theta_1^{\boldsymbol{\xi}[1]}$ from its neighbor, denoted by k, in layer 1. Note that $\theta_1^{\boldsymbol{\xi}[1]}$ is the probability that k is a cooperator in layer 1. Individual j pays cost $c\theta_0^{\boldsymbol{\xi}[1]}$ because it is a cooperator in layer 1. The payoff to j from layer 2 is equal to $r\phi_{0,0}^{\boldsymbol{\xi}[1,2]} + \left(1 - \phi_{0,0}^{\boldsymbol{\xi}[1,2]}\right)$. Note that $\phi_{0,0}^{\boldsymbol{\xi}[1,2]}$ is the probability that j's strategy is the same between layers 1 and 2; in our analysis, this means j is a mutant in layer 2 given that j is a cooperator in layer 1. Therefore, the total payoff of j is equal to $b\theta_1^{\boldsymbol{\xi}[1]} - c\theta_0^{\boldsymbol{\xi}[1]} + r\phi_{0,0}^{\boldsymbol{\xi}[1,2]} + \left(1 - \phi_{0,0}^{\boldsymbol{\xi}[1,2]}\right)$.

Similarly, individual ℓ receives an average benefit $b\theta_3^{\boldsymbol{\xi}[1]}$ from its neighbor, denoted by k', in layer 1. Note that $\theta_3^{\boldsymbol{\xi}[1]}$ is the probability that k' is a cooperator in layer 1 because k' is a three-step neighbor of cooperator j (i.e., $j \to i \to \ell \to k'$). Individual ℓ pays cost $c\theta_2^{\boldsymbol{\xi}[1]}$ because ℓ is a two-step neighbor of j and hence is a cooperator with probability $\theta_2^{\boldsymbol{\xi}[1]}$. The payoff to ℓ from layer 2 is equal to $r\phi_{2,0}^{\boldsymbol{\xi}[1,2]} + \left(1 - \phi_{2,0}^{\boldsymbol{\xi}[1,2]}\right)$. Note that $\phi_{2,0}^{\boldsymbol{\xi}[1,2]}$ is the probability that ℓ is a mutant in layer 2 given that j is a cooperator in layer 1. Therefore, the total payoff of ℓ is equal to $b\theta_3^{\boldsymbol{\xi}[1]} - c\theta_2^{\boldsymbol{\xi}[1]} + r\phi_{2,0}^{\boldsymbol{\xi}[1,2]} + \left(1 - \phi_{2,0}^{\boldsymbol{\xi}[1,2]}\right)$.

Therefore, selection favors cooperation in layer 1 if

$$b\theta_1^{\boldsymbol{\xi}[1]} - c\theta_0^{\boldsymbol{\xi}[1]} + r\phi_{0,0}^{\boldsymbol{\xi}[1,2]} + \left(1 - \phi_{0,0}^{\boldsymbol{\xi}[1,2]}\right) > b\theta_3^{\boldsymbol{\xi}[1]} - c\theta_2^{\boldsymbol{\xi}[1]} + r\phi_{2,0}^{\boldsymbol{\xi}[1,2]} + \left(1 - \phi_{2,0}^{\boldsymbol{\xi}[1,2]}\right). \tag{S74}$$

By substituting $n = 0$ in Eq. (S47), we obtain

$$\theta_0^{\boldsymbol{\xi}[1]} = \sum_{i,j=1}^{N} \pi_i^{[1]} \left(p^{[1]}\right)_{ij}^{(0)} \beta_{ij}^{\boldsymbol{\xi}[1]} = \sum_{i=1}^{N} \pi_i^{[1]} \beta_{ii}^{\boldsymbol{\xi}[1]} = 0. \tag{S75}$$

The last equality comes from Eq. (S37). By substituting $m = n = 0$ in Eq. (S48), we obtain

$$\phi_{0,0}^{\boldsymbol{\xi}[1,2]} = \sum_{i,j=1}^{N} \pi_i^{[1]} \left(p^{[1,2]}\right)_{ij}^{(0,0)} \gamma_{ij}^{\boldsymbol{\xi}[1,2]} = \sum_{i=1}^{N} \pi_i^{[1]} \gamma_{ii}^{\boldsymbol{\xi}[1,2]} = 0. \tag{S76}$$

The last equality comes from Eq. (S38). By substituting Eqs. (S75) and (S76) in Eq. (S74), we obtain

$$c\theta_2^{\boldsymbol{\xi}[1]} + b\left(\theta_1^{\boldsymbol{\xi}[1]} - \theta_3^{\boldsymbol{\xi}[1]}\right) - (r-1)\,\phi_{2,0}^{\boldsymbol{\xi}[1,2]} > 0, \tag{S77}$$

which is Eqs. (3) and (S77).

One can derive a parallel explanation of the condition under which the mutant is favored. We assume that individual i updates its type in layer 2, that j is a mutant neighbor of i in layer 2, and that ℓ is a random neighbor of i in layer 2. Selection favors the mutant in layer 2 if

$$r\theta_0^{\boldsymbol{\xi}[2]} + \left(1 - \theta_0^{\boldsymbol{\xi}[2]}\right) + b\phi_{1,0}^{\boldsymbol{\xi}[1,2]} - c\phi_{0,0}^{\boldsymbol{\xi}[1,2]} > r\theta_2^{\boldsymbol{\xi}[2]} + \left(1 - \theta_2^{\boldsymbol{\xi}[2]}\right) + b\phi_{1,2}^{\boldsymbol{\xi}[1,2]} - c\phi_{0,2}^{\boldsymbol{\xi}[1,2]}. \tag{S78}$$

By substituting Eqs. (S75) and (S76) in Eq. (S78), we obtain

$$-(r-1)\,\theta_2^{\boldsymbol{\xi}[2]} + c\phi_{0,2}^{\boldsymbol{\xi}[1,2]} + b\left(\phi_{1,0}^{\boldsymbol{\xi}[1,2]} - \phi_{1,2}^{\boldsymbol{\xi}[1,2]}\right) > 0, \tag{S79}$$

which is Eq. (4).

Text E Impact of the network structure and initial condition on promotion of cooperation

E.1 Impact of the network structure

To explore what structural properties of networks promote prosociality, for all the single-layer six-node connected networks, we measured the correlation between each pair of the five indices: $\theta_1^{\boldsymbol{\xi}[1]} - \theta_3^{\boldsymbol{\xi}[1]}$ (which we denote by $\theta_1 - \theta_3$ in this section, as we do in the main text), $n_{\text{init,C}}$, which is the number of initial conditions out of the six initial conditions (i.e., choice of the single node that initially has the cooperator) for which $(b/c)^* > 0$, mean degree, clustering coefficient, and mean closeness centrality. Quantities $\theta_1 - \theta_3$ and $n_{\text{init,C}}$ are measures of prosociality for a network. The other three indices quantify the network structure. We regarded the 112 six-node networks as samples based on which we compute the Pearson correlation coefficient. We only consider the dB-dB rule for simplicity.

We show the Pearson correlation coefficients in Fig A. As reported in the main text, $\theta_1 - \theta_3$ and $n_{\text{init,C}}$ are strongly positively correlated. These two quantities are strongly negatively correlated with the three network metrics. Therefore, cooperation is easier for networks with a smaller mean degree, smaller clustering coefficient, and smaller mean closeness. Because the effect of the small mean degree of the network on enhancing fixation of cooperation is established [12, 13], we also compute partial correlation coefficients by partialing out the effect of the mean degree. The partial correlation with $\theta_1 - \theta_3$ is equal to -0.46 and -0.65 for the clustering coefficient and mean closeness, respectively. The partial correlation with $n_{\text{init,C}}$ is equal to -0.23 and -0.35 for the clustering coefficient and mean closeness, respectively. Therefore, the result that a small clustering coefficient and small closeness centrality tend to promote cooperation is not a byproduct of the effect of the small mean degree.

The result that the small clustering coefficient promotes cooperation contradicts a common notion of spatial reciprocity with which clustering of cooperators in networks enhances cooperation [14–16]. We do not know the reason for this discrepancy. The discrepancy may be because here we investigate the fixation probability under weak selection, whereas a majority of studies on spatial reciprocity has examined metastable states under strong selection.

Next, we examine the extent to which the coupling with the constant-selection layer changes cooperation. Under the dB-dB updating rule, we now consider all the $31 \times 112 = 3,584$ ordered pairs of connected six-node networks as layers 1 and 2 of the two-layer network. For layer 1, we only consider networks that yield $(b/c)^* > 0$ for any initial condition. For layer 2, we consider all the connected six-node networks. We examined how the sensitivity of $(b/c)^*$ in layer 1 to the change in the mutant's fitness in layer 2, quantified by $|\mathrm{d}(b/c)^*/\mathrm{d}r|$, depends on the network structure of layers 1 and 2. As we did for Fig A, we compute the Pearson correlation coefficient between $|\mathrm{d}(b/c)^*/\mathrm{d}r|$ and each network metric, regarding each two-layer network as a sample.

We show the results in Table A. We find that $|\mathrm{d}(b/c)^*/\mathrm{d}r|$ is strongly correlated with several metrics of the layer-1 network. In contrast, it is uncorrelated with all of the layer-2 metrics (with the absolute value of the Pearson correlation coefficient < 0.045). A large mean degree tends to yield a large mean closeness, small diameter, and large algebraic connectivity. Therefore, a primary implication of Table A is that a large mean degree of layer 1, not layer 2, makes $(b/c)^*$ of the two-layer network relatively sensitive to variations in r.

We repeated the same analysis for larger model and empirical two-layer networks in which the layer-1 network allows cooperation (i.e., $(b/c)^* > 0$). Specifically, we used two-layer ER and BA networks with $N = 15$, 30, 50, 75, and 100 individuals, one instance each, and the LLF network. We excluded the VC7 network because its layer-1 network yields $(b/c)^* < 0$. As a result, we have 11 two-layer networks, which we regard as samples for computing the Pearson correlation coefficient between $|\mathrm{d}(b/c)^*/\mathrm{d}r|$ and metrics of network structure. We show the results in Table B. We only show the correlation between $|\mathrm{d}(b/c)^*/\mathrm{d}r|$ and the metrics of the layer-1 network, not the layer-2 network, because in the two-layer ER and BA networks, layers 1 and 2 are statistically the same (e.g., the two layers would have similar variances of

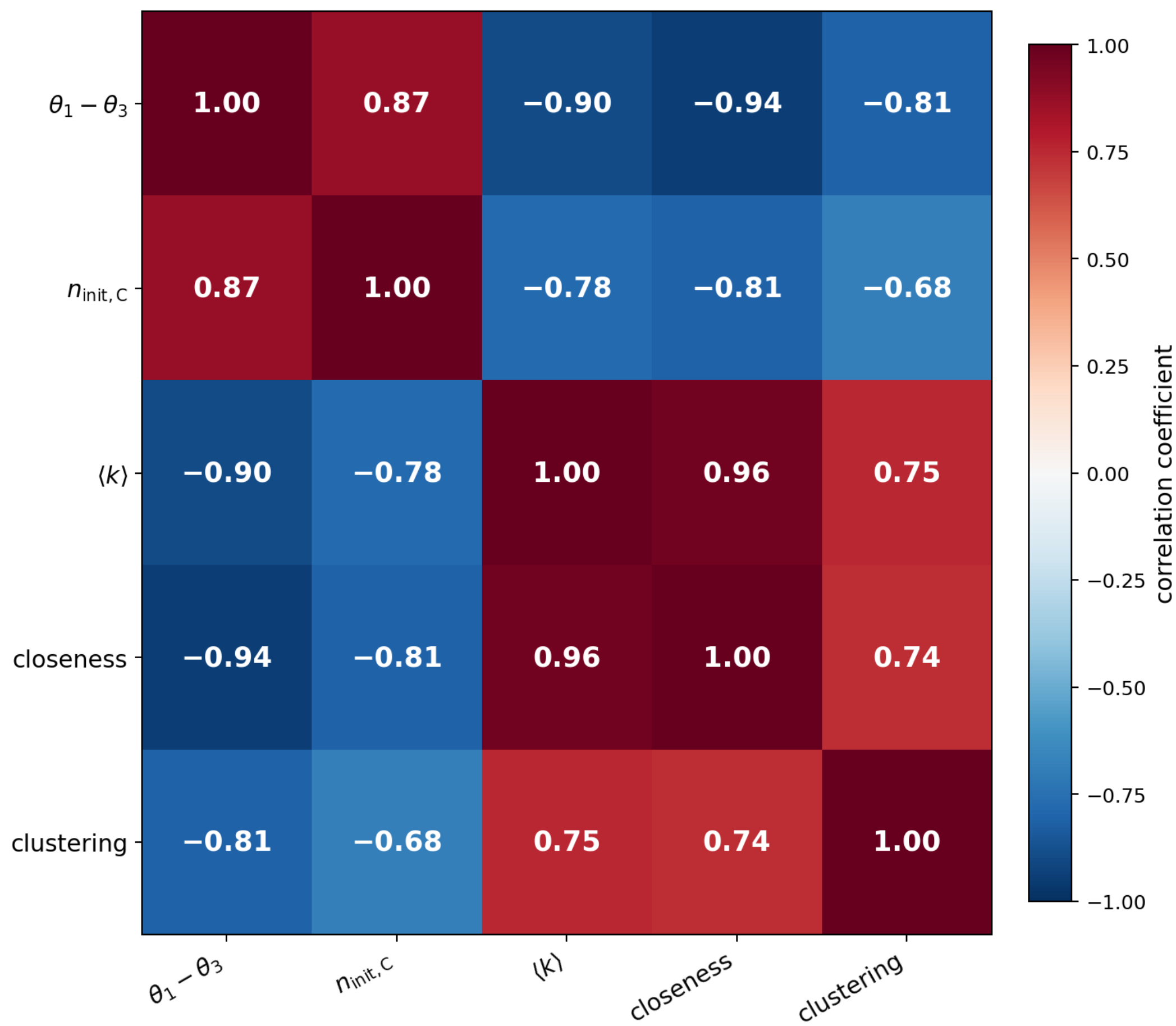

Fig A. Association between prosociality and network structure for one-layer networks with six nodes under the dB-dB rule. The values shown are the Pearson correlation coefficient between pairs of metrics. Two metrics are related to prosociality (i.e., $\theta_1 - \theta_3$ and $n_{\mathrm{init,C}}$). The other three metrics quantify network structure (i.e., mean degree (denoted by $\langle k \rangle$), clustering coefficient, and mean closeness centrality).

Table A. Relationship between $|\mathrm{d}(b/c)^*/\mathrm{d}r|$ and properties of the two-layer network. Each value shown is the Pearson correlation coefficient between $|\mathrm{d}(b/c)^*/\mathrm{d}r|$ and each network metric measured for one network layer. We used the two-layer networks in which the layer-1 network has $(b/c)^* > 0$ regardless of the initial condition. For larger networks, we used the ER and BA networks with $N = 15$, 30, 50, 75, and 100 nodes, and the LLF network. We excluded the VC7 network because its layer-1 network has $(b/c)^* < 0$.

network size	six-node		larger
network layer	1	2	1
mean degree	0.855	−0.040	0.641
maximum degree	0.284	−0.039	0.481
variance of degree	−0.081	−0.014	0.736
mean closeness	0.831	−0.044	0.750
clustering coefficient	0.286	−0.029	0.834
diameter	−0.532	0.036	−0.532
algebraic connectivity	0.722	−0.034	0.812

the degree). We find how properties of network layer 1 affect $|\mathrm{d}(b/c)^*/\mathrm{d}r|$ is largely similar to the case of networks with $N = 6$ individuals, except the effect of the variance of the degree. This discrepancy may be because the degree range of networks with $N = 6$ under the constraint that the network has just a single connected component is inherently limited.

E.2 Impact of the initial condition

In this section, we investigate the impact of the initial condition on the ease of cooperation for single-layer networks. To this end, we focus on the 32 out of the 112 six-node networks for which all the six initial conditions (i.e., the node on which the single cooperator initially resides, denoted by v) yield $(b/c)^* > 0$, such that cooperation occurs for a sufficiently large b/c. We removed the ring network, which is one of the 32 networks. This is because all nodes in the ring network are vertex-transitive (i.e., structurally the same due to symmetry), and therefore one cannot ask the impact of the initial condition (i.e., the location of the single initial cooperator) on fixation of cooperation. For each of the remaining 31 networks, we computed the Pearson correlation coefficient between $(b/c)^*$ given the initial condition, v, and v's centrality. The node centralities used are the degree, local clustering coefficient, closeness centrality, and betweenness centrality. We confirmed that, except for the local clustering coefficient, for each of the 31 networks, some nodes have different centrality values such that one can compute the Pearson correlation coefficient. For the local clustering coefficient, all nodes have value 0 for some networks because they are triangle-free. We excluded these networks because one cannot compute the correlation coefficient. This left us 16 networks for examining the effect of the local clustering coefficient on $(b/c)^*$. We also note that nodes with degree 1 are assigned with the local clustering coefficient value of 0 for simplicity. We include the betweenness centrality in this analysis while we exclude the mean betweenness centrality in Fig A. This is because we consider that it is not appropriate to compare the betweenness centrality across different networks. When computing the correlation coefficient for a given network, we regard each of the six nodes as sample, while we are aware that the computed correlation coefficients are not sufficiently reliable because they are only based on six samples.

We show the obtained correlation coefficients in Fig B. We find that the initial cooperator located at a node with a large degree or closeness tends to suppress fixation of cooperation (by raising $(b/c)^*$), albeit modestly. The betweenness centrality is not correlated with $(b/c)^*$. Nodes with a high local clustering coefficients tend to suppress cooperation as well.

We have also investigated the correlation between $(b/c)^*$ and the centralities of v in larger single-layer ER, BA, and empirical networks allowing cooperation (i.e., $(b/c)^* > 0$). We show the results in Table B. Although the node's degree in the BA networks is not positively correlated with $(b/c)^*$, the results are

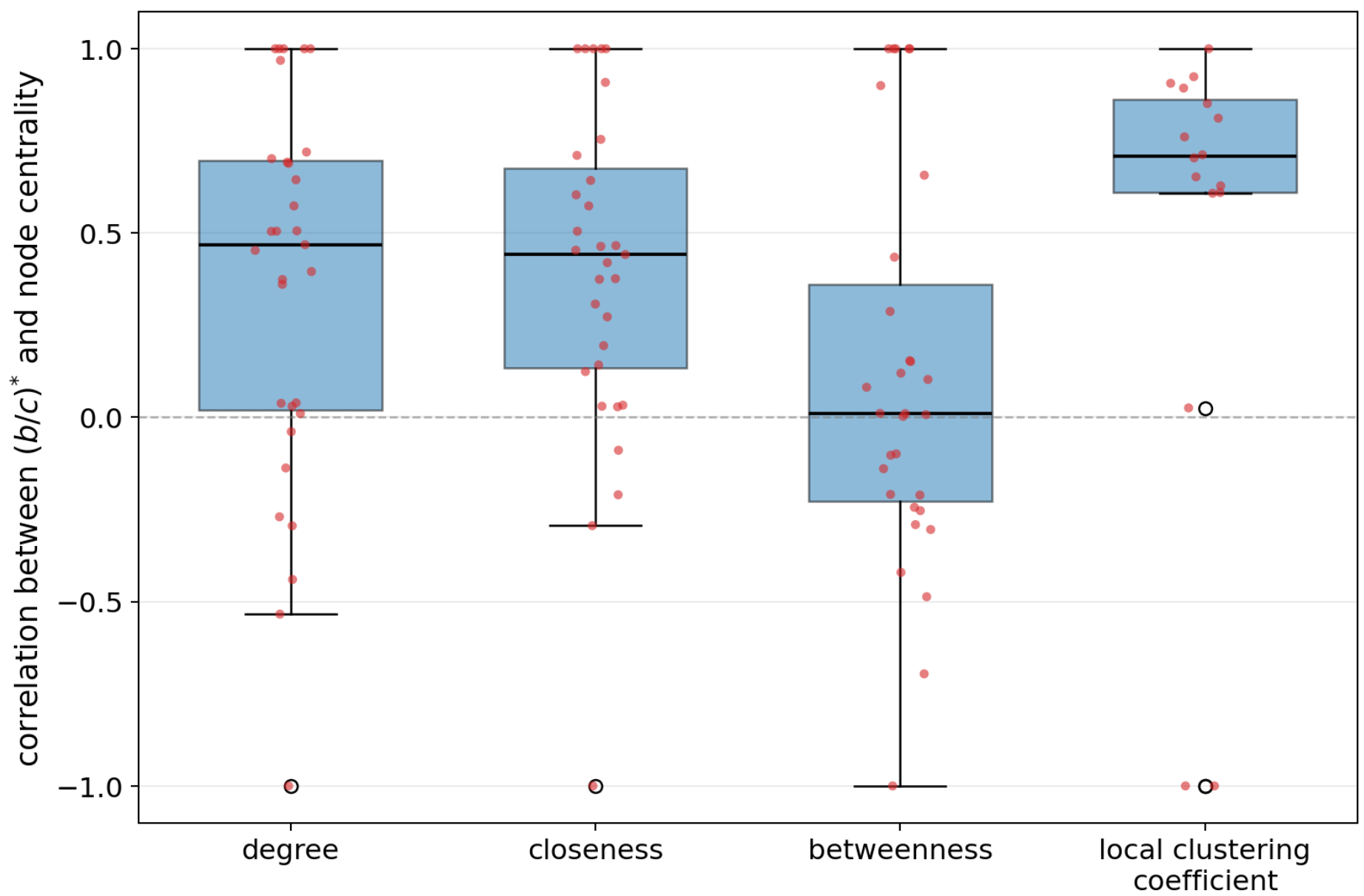

Fig B. Correlation between $(b/c)^*$ and the initial condition for the six-node networks that yield $(b/c)^* > 0$ regardless of the initial condition. A filled dot represents a network, jittered horizontally to avoid overlaps. An open circle represents an outlier. The vertical axis represents the Pearson correlation coefficient between $(b/c)^*$ and the node's centrality. The rectangular boxes span from the first quartile (Q_1) to the third quartile (Q_3). The thick horizontal lines represent the median. The whiskers span from the minimum without outliers ($Q_1 - 1.5 \times \text{IQR}$) to the maximum without outliers ($Q_3 + 1.5 \times \text{IQR}$), where $\text{IQR} = Q_3 - Q_1$.

largely consistent with those for the networks with $N = 6$ nodes shown in Fig B.

Table B. Correlation between $(b/c)^*$ and the initial condition for larger networks. Each value shown is the Pearson correlation coefficient between $(b/c)^*$ and the centrality of the node that initially has a cooperator. We denote by "local clus." the local clustering coefficient. We used the single-layer networks that yield $(b/c)^* > 0$ regardless of the initial condition, which excluded layer 1 of the VC7 network. Layer 1 of the LLF network is included. The local clustering coefficient cell is not filled for the ER network with $N = 15$ because the network instance we obtained is triangle-free.

network	degree	closeness	betweenness	local clus.
ER, $N = 15$	0.46	0.91	0.87	N/A
ER, $N = 30$	0.46	0.67	0.00	0.25
ER, $N = 50$	0.54	0.78	0.13	0.34
ER, $N = 75$	0.49	0.63	0.16	0.30
ER, $N = 100$	0.55	0.67	0.18	0.31
BA, $N = 15$	0.15	0.30	0.13	0.27
BA, $N = 30$	−0.09	0.19	−0.06	0.63
BA, $N = 50$	−0.11	0.28	−0.06	0.51
BA, $N = 75$	−0.10	0.43	−0.06	0.58
BA, $N = 100$	−0.09	0.38	−0.06	0.40
LLF	0.80	0.77	0.51	−0.17

Text F Strategy-type correlation between the two layers during evolutionary dynamics

In this section, we numerically investigate possible correlation between the individual's strategy in the game network layer (i.e., cooperation or defection) and its type in the constant-selection network layer (i.e., resident or mutant) during the evolutionary dynamics. We focus on two-layer networks with $N = 6$ of which the layer-1 network fosters cooperation as single-layer network (i.e., $(b/c)^* > 0$). We use five such two-layer networks chosen as follows. We first impose that the average of $\theta_1^{\boldsymbol{\xi}[1]} - \theta_3^{\boldsymbol{\xi}[1]}$ over the six correlated initial conditions, or the initial conditions in which the initial cooperator in layer 1 and the initial mutant in layer 2 are the same individual, is larger than 0.1. We impose this condition because $\theta_1^{\boldsymbol{\xi}[1]} - \theta_3^{\boldsymbol{\xi}[1]}$ is the denominator of $|\mathrm{d}(b/c)^*/\mathrm{d}r|$ and we decide to avoid the cases which involves the division by a quantity near 0. Then, among the qualified two-layer networks, we choose the five networks that are the largest in terms of the average of $\left|\phi_{2,0}^{\boldsymbol{\xi}[1,2]}\right|$ over the six correlated initial conditions divided by the average of $\left|\theta_1^{\boldsymbol{\xi}[1]} - \theta_3^{\boldsymbol{\xi}[1]}\right|$ over the six correlated initial conditions. These five networks make $|\mathrm{d}(b/c)^*/\mathrm{d}r| = \left|\phi_{2,0}^{\boldsymbol{\xi}[1,2]} / \left(\theta_1^{\boldsymbol{\xi}[1]} - \theta_3^{\boldsymbol{\xi}[1]}\right)\right|$ large and therefore enhance prosociality by relatively a large amount when one couples the donation game network layer with the constant-selection network layer. It turns out that all the five networks have $\mathrm{d}(b/c)^*/\mathrm{d}r < 0$. Therefore, we set $r = 4$ such that $(b/c)^*$ for each of these two-layer networks remains positive and is smaller than $(b/c)^*$ for the one-layer network.

For each of the five two-layer networks, we use two initial conditions, each with just one cooperator in layer 1 and one mutant in layer 2. In the first initial condition, the same individual is the cooperator in layer 1 and the mutant in layer 2. As we already mentioned earlier in this section, we call this initial condition correlated. In the second initial condition, the cooperator in layer 1 and the mutant in layer 2 are different individuals. We all this initial condition uncorrelated. Then, we tracked the Pearson correlation coefficient between the state of nodes in layer 1 (by encoding defector and cooperator into 0 and 1, respectively) and that of nodes in layer 2 (by encoding resident and mutant into 0 and 1, respectively). The correlated initial condition has the correlation coefficient equal to 1 initially. The interest is whether or not high correlation is sustained during evolutionary dynamics, especially in runs that end up with fixation of cooperation. Likewise, we are interested in whether correlation between the state in layer 1 and layer 2 builds up during evolutionary dynamics starting with the uncorrelated initial condition. We run 5×10^5 simulations with each initial condition (i.e., either the correlated or uncorrelated initial condition) for each of the five two-layer networks. Then, we compute the average correlation coefficient across surviving runs (i.e., runs that have not reached the fixation of cooperation or defection or the fixation of residents and mutants; for computing the correlation coefficient, layer 1 has to have at least one cooperator and one defector, and layer 2 has to have at least one resident and one mutant) at each time point. We use the dB-dB rule and set the selection strength to $\delta = 0.02$. We set $c = 1$. We set b such that b/c is 1.5 times larger than $(b/c)^*$ for the two-layer network with the initial condition used in the panel (i.e., correlated or uncorrelated). This b value is favorable to cooperators, but due to the sufficiently weak selection (i.e., $\delta = 0.02$), the fixation probability of cooperation observed in the following numerical simulations is not much distant from that for the neutral dynamics (i.e., $\delta = 0$).

We show the dynamics of the state correlation for the five two-layer networks and two initial conditions in Fig C. For the correlated initial condition, shown in Fig C(a)–(e), the correlation decays towards zero over time no matter whether the cooperator or defector eventually fixates. The deviation of the correlation from 0 near the end of the evolutionary dynamics is due to the small number of runs surviving to that time, so we regard it as statistical noise. Therefore, the state-to-state correlation between the two layers does not underlie the enhanced fixation probability for cooperation. In the case of the uncorrelated initial condition, shown in Fig C(f)–(j), strong state-to-state correlation does not build up through evolutionary dynamics, while we see transient Pearson correlation coefficient values up to ≈ 0.25 in Fig C(f), (h), and (i). However, even in these three networks, the runs in which cooperation fixates do not show notable tendencies. Therefore, although the present numerical simulations are of a small scale and not

exhaustive, we suggest that state-to-state correlation is not a mechanism of fixation of cooperation in two-layer networks.

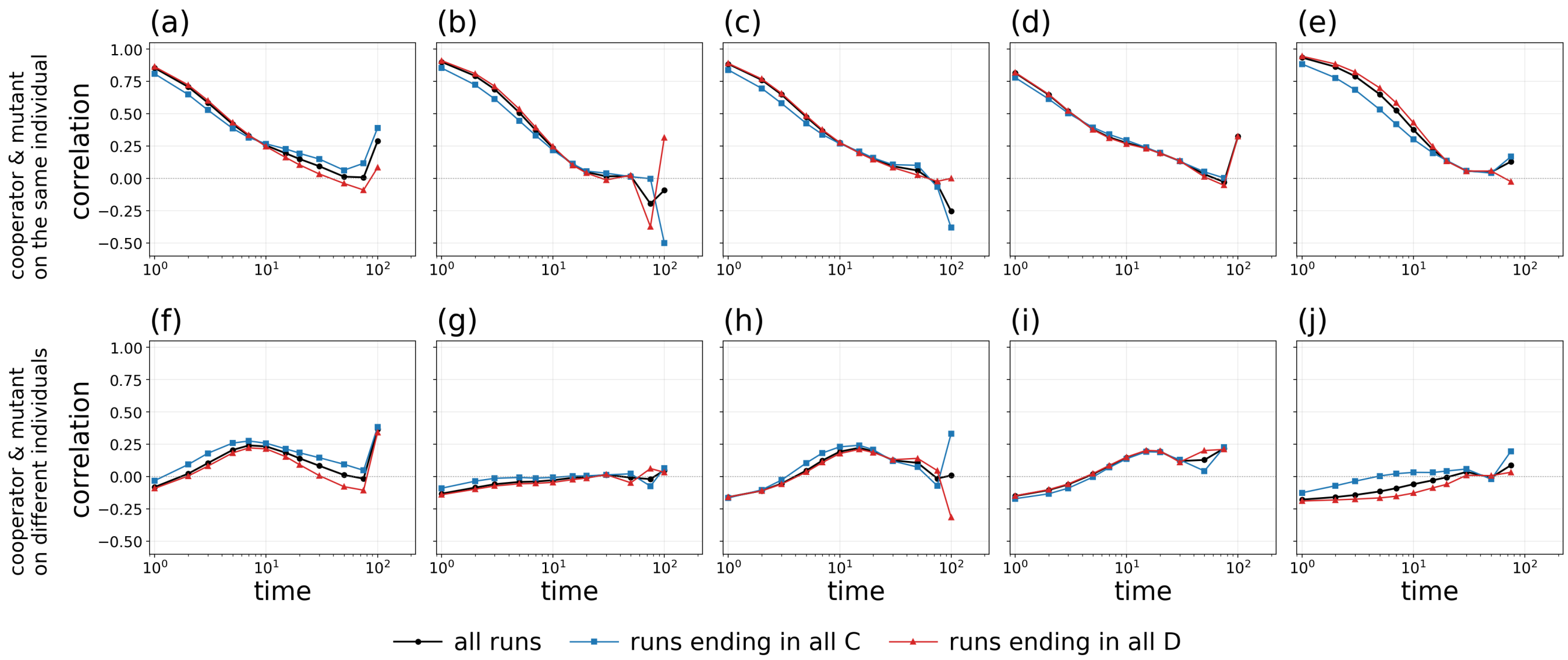

Fig C. Dynamics of the correlation between the states in layer 1 (i.e., defection or cooperation) and those in layer 2 (i.e., resident or mutant). We use five two-layer networks with $N = 6$ individuals. (a)–(e): Correlated initial condition. (f)–(j): Uncorrelated initial condition. Panels (a) and (f), for example, are for the same two-layer network. At each time during the evolutionary dynamics, we show the correlation computed based on all the 5×10^5 runs, all runs that ended up with fixation of cooperation, and all runs that ended up with fixation of defection, shown by the lines in different colors. The $(b/c)^*$ value for the layer-1 network is equal to 28.32 in (a), (b), (e), (f), (g), and (j); the layer-1 network is identical in these three two-layer networks. (Note that we use the same two-layer network in (a) and (f), we use another two-layer network in (b) and (g), and so forth.) The $(b/c)^*$ value for the layer-1 network is equal to 10.72 in (c), (d), (h), and (i); the layer-1 network is identical in these two two-layer networks. The $(b/c)^*$ value for the two-layer network with $r = 4$ is equal to: (a) 6.75, (b) 8.11, (c) 2.65, (d) 1.80, (e) 7.53, (f) 26.47, (g) 31.31, (h) 10.23, (i) 11.43, and (j) 32.45. Note that $(b/c)^*$ increases for the two-layer network relative to the one-layer network in (g), (i), and (j), making cooperation harder in two-layer than one-layer networks. This is not a contradiction because we have chosen these five two-layer networks to maximize the decrease in $(b/c)^*$ by layer coupling for the correlated initial condition; this does not guarantee that $(b/c)^*$ decreases by layer coupling for the uncorrelated initial condition used in (f)–(j). We set $c = 1$ in all panels. We set b to (a) 10.12, (b) 12.17, (c) 3.98, (d) 2.70, (e) 11.29, (f) 39.71, (g) 46.96, (h) 15.34, (i) 17.15, and (j) 48.68. These b values yield $b/c \approx 1.5 \times (b/c)^*$ for each two-layer network. The numerically obtained fixation probability for cooperation, which is the fraction of all runs that terminate with the fixation of cooperation, is: (a) 0.143, (b) 0.144, (c) 0.073, (d) 0.071, (e) 0.143, (f) 0.142, (g) 0.142, (h) 0.072, (i) 0.073, and (j) 0.142.

Text G Effects of the network structure and initial condition in layer 1 on $\frac{\partial r^*}{\partial b}$ and $\frac{\partial r^*}{\partial c}$

To explore reasons for striped patterns in Fig 3 in the main text, we computed the distribution of $\left(\frac{\partial r^*}{\partial b}, \frac{\partial r^*}{\partial c}\right)$ for arbitrarily selected four layer-1 networks with $N = 6$ individuals. For each layer-1 network, we first fixed an initial condition in layer 1, i.e., the sole replica node initially hosting a cooperator. Then, for all combinations of one of the four arbitrarily selected layer-2 network, its isomorphism, and initial condition (i.e., the sole replica node in layer 2 that initially hosts the mutant), we computed $\left(\frac{\partial r^*}{\partial b}, \frac{\partial r^*}{\partial c}\right)$. We avoided using all possible networks as the layer-2 network because doing so would make scattergrams too crowded.

We show $\left(\frac{\partial r^*}{\partial b}, \frac{\partial r^*}{\partial c}\right)$ for each pair of the layer-1 network and its initial condition in Fig D. Each filled dot represents the result for a pair of the layer-2 network and its initial condition. The first six panels show the results for one arbitrarily selected layer-1 network. Each of the six panels corresponds to an initial condition in layer 1. We visualize the layer-1 network and its initial condition within each panel; we recall that the black and orange circles represent the resident and mutant replica nodes, respectively. The next six panels show the results for a second layer-1 network, and so forth. Some panels are blank because the corresponding initial condition is the same as one that appeared in an earlier panel due to the symmetry of the network. For example, in the top row of Fig D, nodes 1 and 2 are structurally equivalent. Therefore, placing the initial cooperator on node 1 versus node 2 produces exactly the same result, which is why the second panel does not show any dots.

We find that different layer-1 networks and their initial conditions produce different sets of stripes of the data points in general. Some stripes are only present in some of the panels. Therefore, we conclude that the network structure and initial condition in layer 1 are partial determinants of the stripe patterns observed in Fig 3 in the main text.

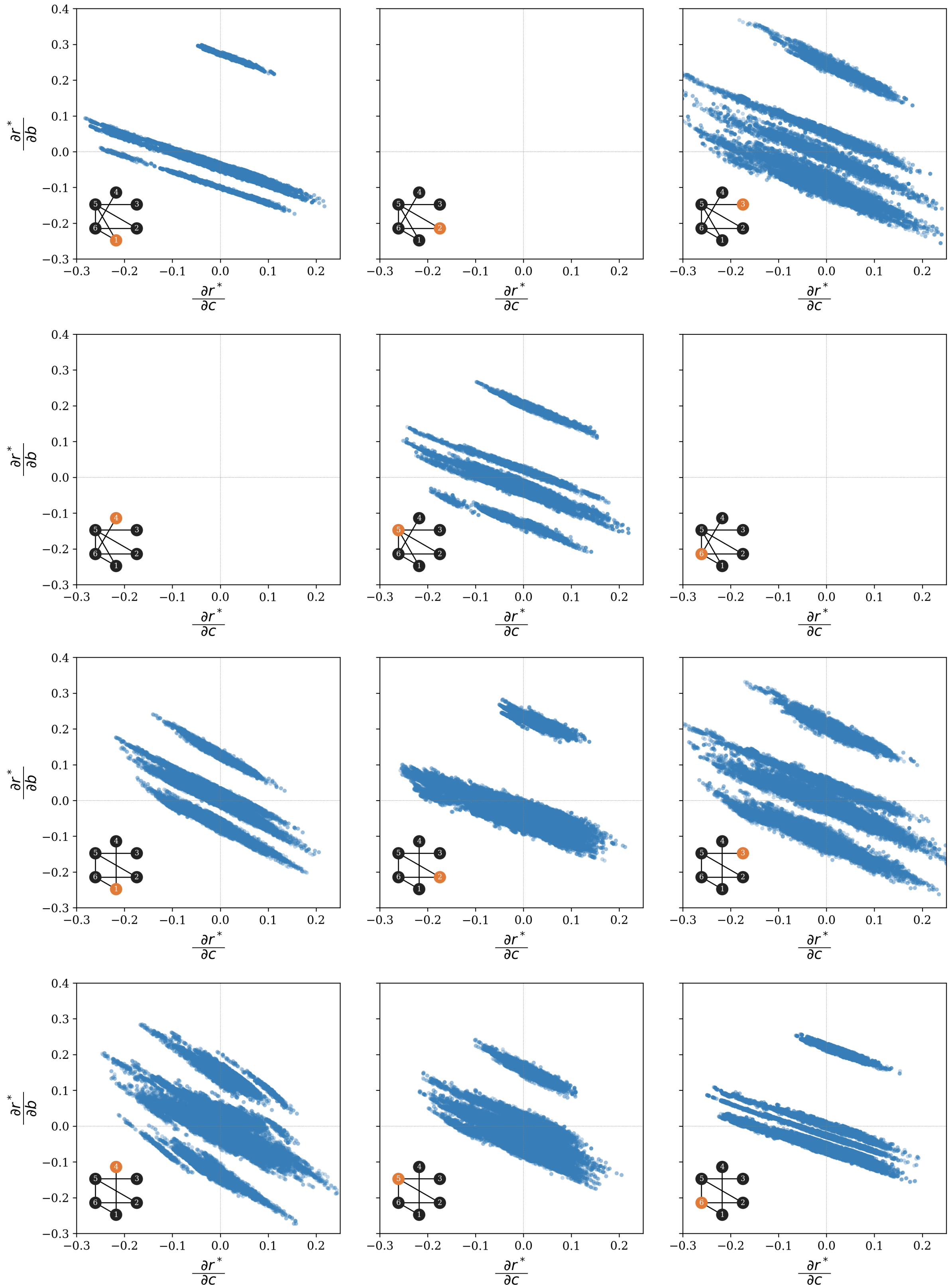

Fig D. Distribution of $\frac{\partial r^*}{\partial b}$ and $\frac{\partial r^*}{\partial c}$ over layer-2 networks and their initial conditions. The visualization of the network within each panel shows the layer-1 network and its initial condition used in the panel. Each filled dot represents a pair of the layer-2 network and its initial condition. Dots are not present in four of the 24 panels because these four cases are the same as a pair of the layer-1 network and its initial condition that appears in an earlier panel.

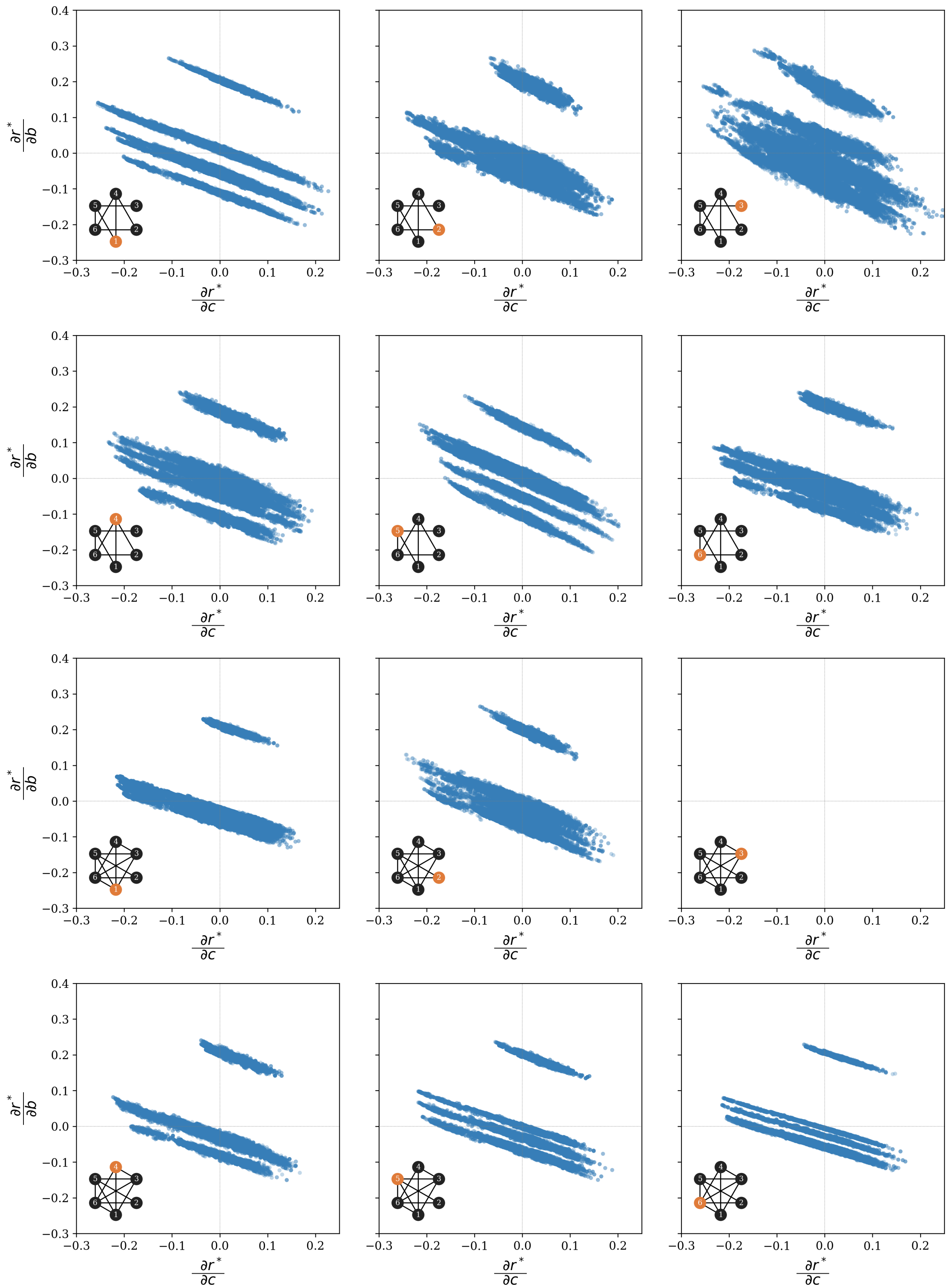

Fig D. (Continued.)

Text H Evolution of the cooperator and mutant on the coupled ring network

We examine the coupled ring network depicted in Fig 4A in the main text under the dB-dB rule. The expressions for $\theta_i^{\boldsymbol{\xi}[1]}$ and $\phi_{m,n}^{\boldsymbol{\xi}[1,2]}$ were derived in Ref. [1]. By following their work, we set $\xi_1^{[1]} = 1$, $\xi_j^{[1]} = 0$ for $j \neq 1$, $\xi_i^{[2]} = 1$, and $\xi_j^{[2]} = 0$ for $j \neq i$. Due to the symmetry of the ring network, this setting covers all possible initial conditions although we assumed $i = 1$ in Fig 4A for demonstration. Let d denote the distance between the node with the initial cooperator in layer 1 and that with the initial mutant in layer 2 when we superpose the two identical rings, one from each layer, to regard the superposition as a single ring network. We obtain

$$\theta_1^{\boldsymbol{\xi}[1]} = -\frac{N-1}{2}, \tag{S80}$$

$$\theta_2^{\boldsymbol{\xi}[1]} = -\frac{N-2}{2}, \tag{S81}$$

$$\theta_3^{\boldsymbol{\xi}[1]} = -\frac{3(N-2)}{4}, \tag{S82}$$

$$\phi_{0,1}^{\boldsymbol{\xi}[1,2]} = -\sum_{\ell=1}^{N-1} \frac{\cos\left(\frac{2\pi\ell d}{N}\right)}{2N - 1 + \cos\left(\frac{2\pi\ell}{N}\right)}, \tag{S83}$$

$$\phi_{2,0}^{\boldsymbol{\xi}[1,2]} = -2(N-1)\phi_{0,1}^{\boldsymbol{\xi}[1,2]} - N\delta_{d,0} + 1, \tag{S84}$$

$$\phi_{2,1}^{\boldsymbol{\xi}[1,2]} = (4N^2 - 6N + 3)\phi_{0,1}^{\boldsymbol{\xi}[1,2]} - \frac{N}{2}\delta_{d,1} + 2N(N-1)\delta_{d,0} - 2N + 3. \tag{S85}$$

By substituting Eqs. (S80)–(S85) in Eq. (3) in the main text, we obtain the condition under which selection favors the cooperator in layer 1 as follows:

$$\begin{aligned} 0 &< c\theta_2^{\boldsymbol{\xi}[1]} + b\left(\theta_1^{\boldsymbol{\xi}[1]} - \theta_3^{\boldsymbol{\xi}[1]}\right) - (r-1)\phi_{2,0}^{\boldsymbol{\xi}[1,2]} \\ &= \frac{b}{4}(N-4) - \frac{c}{2}(N-2) + (r-1)\left[2(N-1)\phi_{0,1}^{\boldsymbol{\xi}[1,2]} + N\delta_{d,0} - 1\right], \end{aligned} \tag{S86}$$

which we can rearrange to

$$\frac{b}{c} > \left(\frac{b}{c}\right)^* \equiv \frac{(N-2)}{2(N-4)} - \frac{4(r-1)}{(N-4)c}\left[2(N-1)\phi_{0,1}^{\boldsymbol{\xi}[1,2]} + N\delta_{d,0} - 1\right] \tag{S87}$$

assuming $N \geq 4$. Equation (S87) suggests that $(b/c)^*$ for the coupled ring network can be moved from the value for the one-layer ring, $(N-2)/\left[2(N-4)\right]$, by choosing $r \neq 1$. Equation (S83) leads to

$$\lim_{N\to\infty} \phi_{0,1}^{\boldsymbol{\xi}[1,2]} = -\int_0^1 \frac{\cos(2\pi dx)}{2}\mathrm{d}x = 0. \tag{S88}$$

By combining Eqs. (S87) and (S88), we obtain

$$\lim_{N\to\infty} \left(\frac{b}{c}\right)^* = \begin{cases} \frac{1}{2} & \text{if } d > 0, \\ \frac{1}{2} - \frac{4(r-1)}{c} & \text{if } d = 0. \end{cases} \tag{S89}$$

Therefore, on large coupled ring networks, the coupling with the constant-selection layer considerably impacts the emergence of cooperation if and only if the initial cooperator and mutant are on the same individual.

Similarly, by substituting Eqs. (S80)–(S85) in Eq. (S61) , we obtain the condition under which selection favors the mutant in layer 2 as follows:

$$
\begin{aligned}
0 &< -(r-1)\theta_2^{\boldsymbol{\xi}[1]} + c\,\phi_{2,0}^{\boldsymbol{\xi}[1,2]} + b\Big(\phi_{0,1}^{\boldsymbol{\xi}[1,2]} - \phi_{2,1}^{\boldsymbol{\xi}[1,2]}\Big) \\
&= \frac{N-2}{2}\,(r-1) - 2(N-1)\,[b(2N-1)+c]\,\phi_{0,1}^{\boldsymbol{\xi}[1,2]} - N\,[2b(N-1)+c]\,\delta_{d,0} + \frac{bN}{2}\,\delta_{d,1} + c + b(2N-3),
\end{aligned}
\tag{S90}
$$

which is equivalent to

$$
r > 1 + \frac{4(N-1)}{N-2}\,[b(2N-1)+c]\,\phi_{0,1}^{\boldsymbol{\xi}[1,2]} + \frac{2N}{N-2}\,[2b(N-1)+c]\,\delta_{d,0} - \frac{bN}{N-2}\,\delta_{d,1} - \frac{c}{N-2} + \frac{b(2N-3)}{N-2}.
\tag{S91}
$$

In terms of the original layer labels, with which layer 1 is the donation game layer and layer 2 is the constant-selection dynamics layer, Eqs. (S90) and (S91) read

$$
\begin{aligned}
0 &< -(r-1)\theta_2^{\boldsymbol{\xi}[2]} + c\,\phi_{0,2}^{\boldsymbol{\xi}[1,2]} + b\Big(\phi_{1,0}^{\boldsymbol{\xi}[1,2]} - \phi_{1,2}^{\boldsymbol{\xi}[1,2]}\Big) \\
&= \frac{N-2}{2}\,(r-1) - 2(N-1)\,[b(2N-1)+c]\,\phi_{1,0}^{\boldsymbol{\xi}[1,2]} - N\,[2b(N-1)+c]\,\delta_{d,0} + \frac{bN}{2}\,\delta_{d,1} + c + b(2N-3)
\end{aligned}
\tag{S92}
$$

and

$$
r > 1 + \frac{4(N-1)}{N-2}\,[b(2N-1)+c]\,\phi_{1,0}^{\boldsymbol{\xi}[1,2]} + \frac{2N}{N-2}\,[2b(N-1)+c]\,\delta_{d,0} - \frac{bN}{N-2}\,\delta_{d,1} - \frac{c}{N-2} + \frac{b(2N-3)}{N-2},
\tag{S93}
$$

respectively.

Text I Evolution of the cooperator and mutant on the coupled complete graph

In this section, we derive the condition for favoring the cooperator and mutant when both layers are the complete graph with N nodes. We assume the unweighted complete graphs without self-loops. We also assume the initial condition used in Fig 4C in the main text, in which the individual cooperating in layer 1 coincides with the mutant in layer 2.

I.1 Derivation of $\theta_n^{\boldsymbol{\xi}[1]}$

We first compute

$$\theta_n^{\boldsymbol{\xi}[1]} = \sum_{i=1}^{N} \sum_{j=1}^{N} \pi_i^{[1]} \left(p^{[1]}\right)_{ij}^{(n)} \beta_{ij}^{\boldsymbol{\xi}[1]}, \tag{S94}$$

for the complete graph. The one-step transition probability matrix of the random walk, $P = \left(p_{ij}^{[1]}\right)$, is given by

$$P = \frac{1}{N-1} (J - I), \tag{S95}$$

where J is matrix with all its entries being 1, and I is the identity matrix. Matrix P has eigenvalues 1 and $\lambda := -\frac{1}{N-1}$ with multiplicities 1 and $N-1$, respectively. The stationary density of the random walk is given by

$$\pi_i^{[1]} = \frac{1}{N}, \quad i \in \{1, \ldots, N\}. \tag{S96}$$

Using $J^2 = NJ$ and the fact that J and I commute, we obtain

$$\begin{aligned}
P^n =& \frac{1}{(N-1)^n} \sum_{k=0}^{n} \binom{n}{k} J^k (-I)^{n-k} \\
=& \left(-\frac{1}{N-1}\right)^n I + \frac{J}{(N-1)^n} \sum_{k=1}^{n} \binom{n}{k} N^{k-1} (-1)^{n-k} \\
=& \frac{1}{N} J + \lambda^n \left(I - \frac{J}{N}\right),
\end{aligned} \tag{S97}$$

where $\binom{n}{k}$ is the binomial coefficient. By substituting Eqs. (S96) and (S97) in Eq. (S94), we obtain

$$\begin{aligned}
\theta_n^{\boldsymbol{\xi}[1]} =& \frac{1}{N} \sum_{i,j=1}^{N} (P^n)_{ij} \, \beta_{ij}^{\boldsymbol{\xi}[1]} \\
=& \frac{1}{N} \sum_{i,j=1}^{N} \left[\frac{1}{N} J_{ij} + \lambda^n \left(\delta_{ij} - \frac{1}{N} J_{ij}\right)\right] \beta_{ij}^{\boldsymbol{\xi}[1]} \\
=& \frac{1}{N^2} \sum_{i,j=1}^{N} J_{ij} \beta_{ij}^{\boldsymbol{\xi}[1]} + \frac{\lambda^n}{N} \left(\sum_{i=1}^{N} \beta_{ii}^{\boldsymbol{\xi}[1]} - \sum_{i,j=1}^{N} \frac{1}{N} J_{ij} \beta_{ij}^{\boldsymbol{\xi}[1]} \right) \\
=& \frac{1}{N^2} \sum_{i,j=1}^{N} \beta_{ij}^{\boldsymbol{\xi}[1]} + \frac{\lambda^n}{N} \left(\sum_{i=1}^{N} \beta_{ii}^{\boldsymbol{\xi}[1]} - \sum_{i,j=1}^{N} \frac{1}{N} \beta_{ij}^{\boldsymbol{\xi}[1]} \right).
\end{aligned} \tag{S98}$$

To simplify Eq. (S98), we note that substitution of $\pi_i = \frac{1}{N}, \forall i$ in Eq. (S37) yields $\sum_{i=1}^{N} \beta_{ii}^{\boldsymbol{\xi}[1]} = 0$. By substituting this equality in Eq. (S98), we obtain

$$
\begin{aligned}
\theta_n^{\boldsymbol{\xi}[1]} &= \frac{1}{N^2} \sum_{i,j=1}^{N} \beta_{ij}^{\boldsymbol{\xi}[1]} + \frac{\lambda^n}{N} \left(\sum_{i=1}^{N} \beta_{ii}^{\boldsymbol{\xi}[1]} - \sum_{i,j=1}^{N} \frac{1}{N} \beta_{ij}^{\boldsymbol{\xi}[1]} \right) \\
&= \frac{1}{N^2} \sum_{i,j=1;i\neq j}^{N} \beta_{ij}^{\boldsymbol{\xi}[1]} + \frac{\lambda^n}{N} \left(0 - \sum_{i,j=1;i\neq j}^{N} \frac{1}{N} \beta_{ij}^{\boldsymbol{\xi}[1]} \right) \\
&= \frac{1-\lambda^n}{N^2} \sum_{i,j=1;i\neq j}^{N} \beta_{ij}^{\boldsymbol{\xi}[1]} \\
&\equiv \frac{1-\lambda^n}{N^2} S_{\text{off}}.
\end{aligned} \tag{S99}
$$

We need to obtain S_{off} from Eq. (S54). To simplify Eq. (S54) for the complete graph, we first note that variables $\xi_i^{[1]}$ and $\xi_j^{[1]}$ are 0 or 1 depending on whether a defector or cooperator, respectively, occupies the node. Because we have only one cooperator in our initial condition, both $\xi_i^{[1]}$ and $\xi_j^{[1]}$, where $i \neq j$, cannot be 1, leading to $\xi_i^{[1]} \xi_j^{[1]} = 0$. The weighted reproductive value $\widehat{\xi}^{[1]} = 1/N$ comes from the definition of the RV-weighted frequency, Eq. (S4), and Eq. (S5). Because all the nodes in the complete graph have the same reproductive value due to symmetry and only one node has a cooperator, the RV-weighted frequency is equal to $1/N$. Therefore, we obtain

$$
\xi_i^{[1]} \xi_j^{[1]} - \hat{\xi}^{[1]} = -\frac{1}{N}. \tag{S100}
$$

By substituting Eq. (S100), $p_{ik}^{[1]} = 1/(N-1)$ for $k \neq i$, and $p_{ik}^{[1]} = 0$ for $k = i$ in Eq. (S54), we obtain

$$
\beta_{ij}^{\boldsymbol{\xi}[1]} = -\frac{1}{2} + \frac{1}{2} \sum_{k=1;k\neq i}^{N} \frac{\beta_{kj}^{\boldsymbol{\xi}[1]}}{N-1} + \frac{1}{2} \sum_{k=1;k\neq j}^{N} \frac{\beta_{ik}^{\boldsymbol{\xi}[1]}}{N-1}, \qquad (i \neq j). \tag{S101}
$$

By summing both sides of Eq. (S101) over the i and j values with $i \neq j$, we obtain

$$
S_{\text{off}} = -\frac{N(N-1)}{2} + \frac{1}{2} \frac{N-2}{N-1} S_{\text{off}} + \frac{1}{2} \frac{N-2}{N-1} S_{\text{off}}. \tag{S102}
$$

Equation (S102) leads to

$$
S_{\text{off}} = -\frac{N(N-1)^2}{2}. \tag{S103}
$$

Substitution Eq. (S103) in Eq. (S99) yields

$$
\theta_n^{\boldsymbol{\xi}[1]} = -\frac{(N-1)^2}{2N} \left[1 - \left(-\frac{1}{N-1} \right)^n \right], \tag{S104}
$$

where $n \in \{1, 2, \ldots\}$.

I.2 Derivation of $\phi_{n,m}^{\boldsymbol{\xi}[1,2]}$

We now compute

$$
\phi_{n,m}^{\boldsymbol{\xi}[1,2]} := \sum_{i,j=1}^{N} \pi_i^{[1]} \left(p^{[1,2]} \right)_{ij}^{(n,m)} \gamma_{ij}^{\boldsymbol{\xi}[1,2]}. \tag{S105}
$$

Because the two layers are both complete graph on N nodes, we obtain

$$\left(p^{[1,2]}\right)^{(n,m)} = P^n P^m = P^{n+m} \tag{S106}$$

and

$$\phi_{n,m}^{\boldsymbol{\xi}[1,2]} = \sum_{i,j=1}^{N} \pi_i^{[1]} \left(p^{[1,2]}\right)_{ij}^{(n,m)} \gamma_{ij}^{\boldsymbol{\xi}[1,2]} = \frac{1}{N} \sum_{i,j} (P^{n+m})_{ij}\, \gamma_{ij}^{\boldsymbol{\xi}[1,2]}, \tag{S107}$$

where we used $\pi_i^{[1]} = 1/N$.

To compute $\gamma_{ij}^{\boldsymbol{\xi}[1,2]}$, which we write γ_{ij} in the remainder of this section for notational simplicity, we set with $\xi_1^{[1]} = \xi_1^{[2]} = 1$ and $\xi_j^{[L]} = 0$ for $j \neq 1$, $L \in \{1, 2\}$. This assumption corresponds to the initial condition that we have assumed, in which the sole initial cooperator in layer 1 is the sole initial mutant in layer 2. We obtain $\widehat{\xi}^{[1]} = \widehat{\xi}^{[2]} = 1/N$ and $\vec{\xi}^{[1]}\vec{\xi}^{[2]\top} - \widehat{\xi}^{[1]}\widehat{\xi}^{[2]} J = U - \frac{1}{N^2} J$, where $\vec{\xi}^{[L]} = (\xi_1^{[L]}, \ldots, \xi_i^{[L]}, \ldots, \xi_N^{[L]})^\top$, $U := \boldsymbol{e}_1 \boldsymbol{e}_1^\top$, and vector $\boldsymbol{e}_1$ is the standard basis vector, i.e., $\boldsymbol{e}_1 = (1, 0, \ldots, 0)^\top$. The expression of γ_{ij} with $i \neq j$, shown in Eq. (S56), simplifies to

$$\gamma_{ij} = \frac{N^2}{2N-1} \left(U_{ij} - \frac{1}{N^2} \right) + \frac{1}{2N-1} (P\gamma P)_{ij} + \frac{N-1}{2N-1} \left((P\gamma)_{ij} + (\gamma P)_{ij} \right). \tag{S108}$$

We also note that $\sum_{i=1}^N \pi_i \gamma_{ii} = 0$ simplifies to

$$\frac{1}{N} \sum_{i=1}^{N} \gamma_{ii} = 0. \tag{S109}$$

Using Eqs. (S97), (S107), and (S109), we obtain

$$\begin{aligned}
\phi_{n,m} &= \frac{1}{N} \left[\sum_{i=1}^{N} \left(\frac{1}{N} + \frac{N-1}{N} \lambda^{n+m} \right) \gamma_{ii} + \sum_{i,j=1; i \neq j}^{N} \left(\frac{1}{N} - \frac{1}{N} \lambda^{n+m} \right) \gamma_{ij} \right] \\
&= \frac{1 - \lambda^{n+m}}{N^2} \sum_{i,j=1; i \neq j}^{N} \gamma_{ij} \\
&\equiv \frac{1 - \lambda^{n+m}}{N^2} \overline{S}_{\text{off}}.
\end{aligned} \tag{S110}$$

To calculate $\overline{S}_{\text{off}}$, we sum Eq. (S108) over all i and j with $i \neq j$. The sum of the first term on the right-hand side of Eq. (S108) becomes

$$\sum_{i,j=1; i \neq j}^{N} \frac{N^2}{2N-1} \left(U_{ij} - \frac{1}{N^2} \right) = \frac{N^2}{2N-1} \left[0 - \frac{N(N-1)}{N^2} \right] = -\frac{N(N-1)}{2N-1}. \tag{S111}$$

The sum of the second term on the right-hand side of Eq. (S108) is written as

$$\sum_{i,j=1; i \neq j}^{N} (P\gamma P)_{ij} = \sum_{k,\ell=1}^{N} \gamma_{k\ell}\, T(k, \ell), \tag{S112}$$

where

$$T(k, \ell) := \sum_{i,j=1; i \neq j}^{N} p_{ik} p_{j\ell}. \tag{S113}$$

Because

$$p_{ik} = \begin{cases} 0 & \text{if } i = k, \\ \frac{1}{N-1} & \text{if } i \neq k, \end{cases} \tag{S114}$$

$T(k,\ell)$ counts ordered pairs (i,j) with $i \neq j$, $i \neq k$, $j \neq \ell$ with weight $(N-1)^{-2}$. Among the $N(N-1)$ pairs of (i,j) with $i \neq j$, there are $2(N-1)$ pairs of (i,j) satisfying $i = k$ or $j = \ell$, if $k = \ell$. If $k \neq \ell$, there are $2(N-1)-1$ such (i,j) pairs. Therefore, we obtain

$$\#\{(i,j)\} = \begin{cases} (N-2)(N-1) & \text{if } k = \ell, \\ (N-2)(N-1)+1 & \text{if } k \neq \ell, \end{cases} \tag{S115}$$

where $\#\{(i,j)\}$ is the number of (i,j) pairs counted in $T(k,\ell)$. Because each (i,j) pair contributes weight $(N-1)^{-2}$, we obtain

$$T(k,\ell) = \begin{cases} \dfrac{N-2}{N-1} & \text{if } k = \ell, \\ \dfrac{N^2-3N+3}{(N-1)^2} & \text{if } k \neq \ell. \end{cases} \tag{S116}$$

By substituting Eq. (S116) in Eq. (S112) and using Eq. (S109), we obtain

$$\sum_{i,j=1;i\neq j}^{N} (P\gamma P)_{ij} = \frac{N^2-3N+3}{(N-1)^2}\,\overline{S}_{\text{off}}. \tag{S117}$$

To calculate the sum of the third term on the right-hand side of Eq. (S108), we first obtain

$$\begin{aligned} \sum_{i,j=1;i\neq j}^{N} (P\gamma)_{ij} &= \sum_{i,j=1;i\neq j}^{N} \sum_{k=1}^{N} p_{ik}\gamma_{kj} \\ &= \sum_{k,j=1}^{N} \gamma_{kj} \sum_{i=1;i\neq j}^{N} p_{ik} \\ &= \sum_{k,j=1}^{N} \gamma_{kj}\,(1-p_{jk}) \\ &= \overline{S}_{\text{off}} - \sum_{k,j=1}^{N} p_{jk}\gamma_{kj} \\ &= \overline{S}_{\text{off}} - \frac{\overline{S}_{\text{off}}}{N-1} \\ &= \frac{N-2}{N-1}\overline{S}_{\text{off}}. \end{aligned} \tag{S118}$$

We used Eq. (S109) to derive the third last equality. We used Eqs. (S109) and (S114) to derive the second last equality. By symmetry, the same holds true for $\sum_{i\neq j}(\gamma P)_{ij}$. Therefore, we obtain

$$\sum_{i,j=1;i\neq j}^{N} ((P\gamma)_{ij} + (\gamma P)_{ij}) = \frac{2(N-2)}{N-1}\overline{S}_{\text{off}}. \tag{S119}$$

By summing Eq. (S108) over all $i,j \in \{1,\ldots,N\}$ with $i \neq j$ and using Eqs. (S111), (S117), and (S119), we obtain

$$\overline{S}_{\text{off}} = -\frac{N(N-1)}{2N-1} + \frac{1}{2N-1}\cdot\frac{N^2-3N+3}{(N-1)^2}\overline{S}_{\text{off}} + \frac{N-1}{2N-1}\cdot\frac{2(N-2)}{N-1}\overline{S}_{\text{off}}. \tag{S120}$$

Equation (S120) leads to

$$\overline{S}_{\text{off}} = -\frac{(N-1)^3}{2N-3}. \tag{S121}$$

By substituting Eq. (S121) and $\lambda = -1/(N-1)$ in Eq. (S110), we obtain

$$\phi_{n,m} = -\frac{(N-1)^3}{N^2(2N-3)}\left[1 - \left(-\frac{1}{N-1}\right)^{n+m}\right]. \tag{S122}$$

I.3 Condition for favoring the cooperator and mutant

By substituting Eqs. (S104) and (S122) in Eq. (3) in the main text, we find that spite is favored on the coupled complete graph if

$$\frac{b}{c} < \left(\frac{b}{c}\right)^* = -(N-1) + \frac{r-1}{c} \cdot \frac{2(N-1)^2}{N(2N-3)}. \tag{S123}$$

When N is large, the first term on the right-hand of Eq. (S123) dominates the second term such that the coupling the two complete graphs does not alter the condition for spite substantially. However, the effect is large when N is small.

The condition for the selection of the mutant, Eq. (4) in the main text, reads

$$A = -\frac{2(N-1)}{N(2N-3)}\left(\frac{b}{N-1} + c\right). \tag{S124}$$

This result implies that r^* is substantially different from 1 if and only if N is small. We also find that the effect of c on r^* is $N-1$ times larger than that of b.

Text J Evolution of the cooperator and mutant on the coupled star graph

We derive the condition for favoring the cooperator and mutant in two-layer star graphs under the dB-dB rule. See Fig E for the network; we focus on this particular two-layer star graph and initial condition with varying N, as in the previous study [1] (see SI Fig 16 of their paper). The expressions for $\theta_i^{\boldsymbol{\xi}[1]}$ and $\phi_{m,n}^{\boldsymbol{\xi}[1,2]}$ were derived [1] as follows:

$$\theta_1^{\boldsymbol{\xi}[1]} = -\frac{2N-3}{2(N-1)}, \tag{S125}$$

$$\theta_2^{\boldsymbol{\xi}[1]} = -\frac{3(N-2)}{4(N-1)}, \tag{S126}$$

$$\theta_3^{\boldsymbol{\xi}[1]} = -\frac{2N-3}{2(N-1)}, \tag{S127}$$

$$\phi_{0,1}^{\boldsymbol{\xi}[1,2]} = -\frac{N\left(8N^5-52N^4+112N^3-107N^2+46N-8\right)}{8(2N-1)(3N-2)(N-1)^4}, \tag{S128}$$

$$\phi_{2,0}^{\boldsymbol{\xi}[1,2]} = -\frac{N^2(N-2)}{2(2N-1)(3N-2)(N-1)^2}, \tag{S129}$$

$$\phi_{2,1}^{\boldsymbol{\xi}[1,2]} = -\frac{N\left(8N^5-44N^4+84N^3-79N^2+38N-8\right)}{8(2N-1)(3N-2)(N-1)^4}. \tag{S130}$$

Note that $\theta_1^{\boldsymbol{\xi}[1]} = \theta_3^{\boldsymbol{\xi}[1]}$ because the star graph is a special case of the complete bipartite graph. By substituting Eqs. (S125)–(S130) in Eq. (3) in the main text, we obtain the condition under which selection favors the cooperator in layer 1 as follows:

$$\begin{aligned} 0 &< c\,\theta_2^{\boldsymbol{\xi}[1]} + b\left(\theta_1^{\boldsymbol{\xi}[1]} - \theta_3^{\boldsymbol{\xi}[1]}\right) - (r-1)\,\phi_{2,0}^{\boldsymbol{\xi}[1,2]} \\ &= -\frac{3c(N-2)}{4(N-1)} + \frac{N^2(N-2)(r-1)}{2(2N-1)(3N-2)(N-1)^2} \\ &= \frac{(N-2)\left[2N^2(r-1) - 3c\,(N-1)(2N-1)(3N-2)\right]}{4(N-1)^2(2N-1)(3N-2)}. \end{aligned} \tag{S131}$$

For $N > 2$, Eq. (S131) is equivalent to

$$2N^2(r-1) - 3c\,(N-1)(2N-1)(3N-2) > 0. \tag{S132}$$

Remarkably, the condition does not depend on b.

Similarly, by substituting Eqs. (S125)–(S130) in Eq. (S61) , we obtain the condition under which selection favors the mutant in the constant-selection layer as follows:

$$\begin{aligned} 0 &< -(r-1)\,\theta_2^{\boldsymbol{\xi}[1]} + c\,\phi_{2,0}^{\boldsymbol{\xi}[1,2]} + b\left(\phi_{0,1}^{\boldsymbol{\xi}[1,2]} - \phi_{2,1}^{\boldsymbol{\xi}[1,2]}\right) \\ &= \frac{3(N-2)(r-1)}{4(N-1)} - c\left[\frac{N^2(N-2)}{2(2N-1)(3N-2)(N-1)^2}\right] \\ &\quad + b\left[-\frac{N\left(8N^5-52N^4+112N^3-107N^2+46N-8\right)}{8(2N-1)(3N-2)(N-1)^4}\right. \\ &\quad \left. + \frac{N\left(8N^5-44N^4+84N^3-79N^2+38N-8\right)}{8(2N-1)(3N-2)(N-1)^4}\right] \\ &= \frac{(N-2)\left\{2N^2\left[b(2N-1)-c(N-1)\right] + 3(N-1)^2(2N-1)(3N-2)(r-1)\right\}}{4(N-1)^3(2N-1)(3N-2)}. \end{aligned} \tag{S133}$$

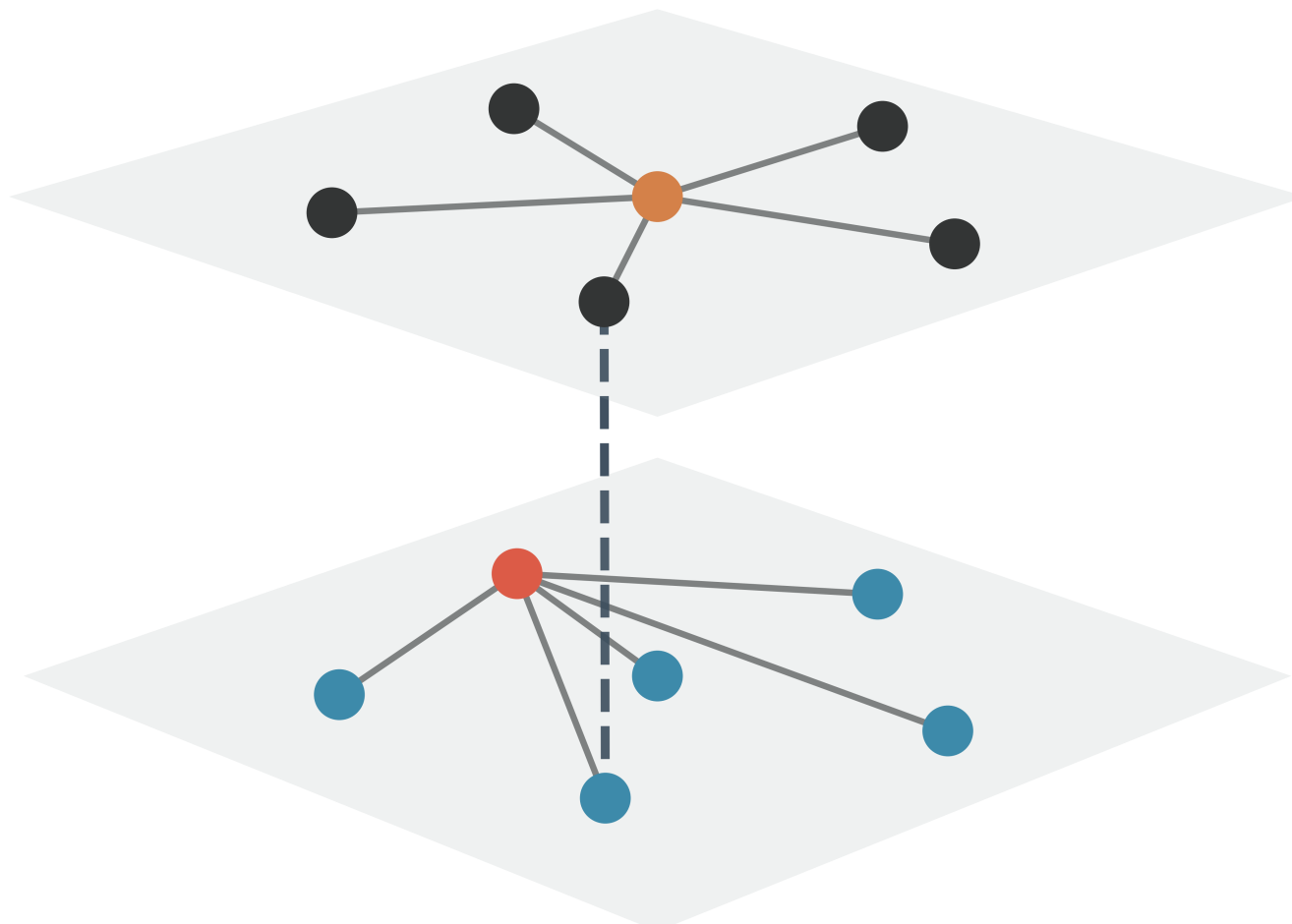

Fig E. Two-layer star graph and the initial condition that we analyze. Note that the hub node in the first layer is not the hub node in layer 2. The node colors are the same as those in Fig 1 in the main text; orange represents the cooperator in layer 1, black represents the defector layer 1, blue represents the resident in layer 2, and red represents the mutant in layer 2. The two replica nodes connected by the dashed line represent the same individual, shown as an example.

For $N > 2$, Eq. (S133) is equivalent to

$$r > 1 - \frac{2N^2\left[b(2N-1) - c(N-1)\right]}{3(N-1)^2(2N-1)(3N-2)}. \tag{S134}$$

We note that the mathematical expression right after the inequality in the first line of Eq. (S133) uses the swapped layer labels, with which the constant-selection dynamics occurs in layer 1 and the donation game occurs in layer 2. In terms of the original layer labels, with which the donation game layer occurs in layer 1 and the constant-selection dynamics occurs in layer 2, the same quantity is expressed as $-(r-1)\,\theta_2^{\boldsymbol{\xi}[2]} + c\,\phi_{0,2}^{\boldsymbol{\xi}[1,2]} + b\left(\phi_{1,0}^{\boldsymbol{\xi}[1,2]} - \phi_{1,2}^{\boldsymbol{\xi}[1,2]}\right)$.

Text K Mean and standard deviation of $\left|\frac{\mathbf{d}(b/c)^*}{\mathbf{d}r}\right|$ for two-layer ER and BA networks with $N = 15$ individuals

We showed in Fig 5 in the main text the median and the 5th and 95th percentiles of $|\mathrm{d}(b/c)^*/\mathrm{d}r|$ for two-layer ER and BA networks with $N = 15$ individuals under the dB-dB and dB-Bd updating rules. Fig F shows the mean and standard deviation of $|\mathrm{d}(b/c)^*/\mathrm{d}r|$ in the corresponding cases. We find that, while the mean does not behave monotonically with respect to the network density parameters (i.e., p_1, p_2, $\overline{m}_1$, and $\overline{m}_2$), the main trend that $|\mathrm{d}(b/c)^*/\mathrm{d}r|$ increases as the density of edges in layer 1 (i.e., p_1 or $\overline{m}_1$) increases remains the same as in Fig 5.

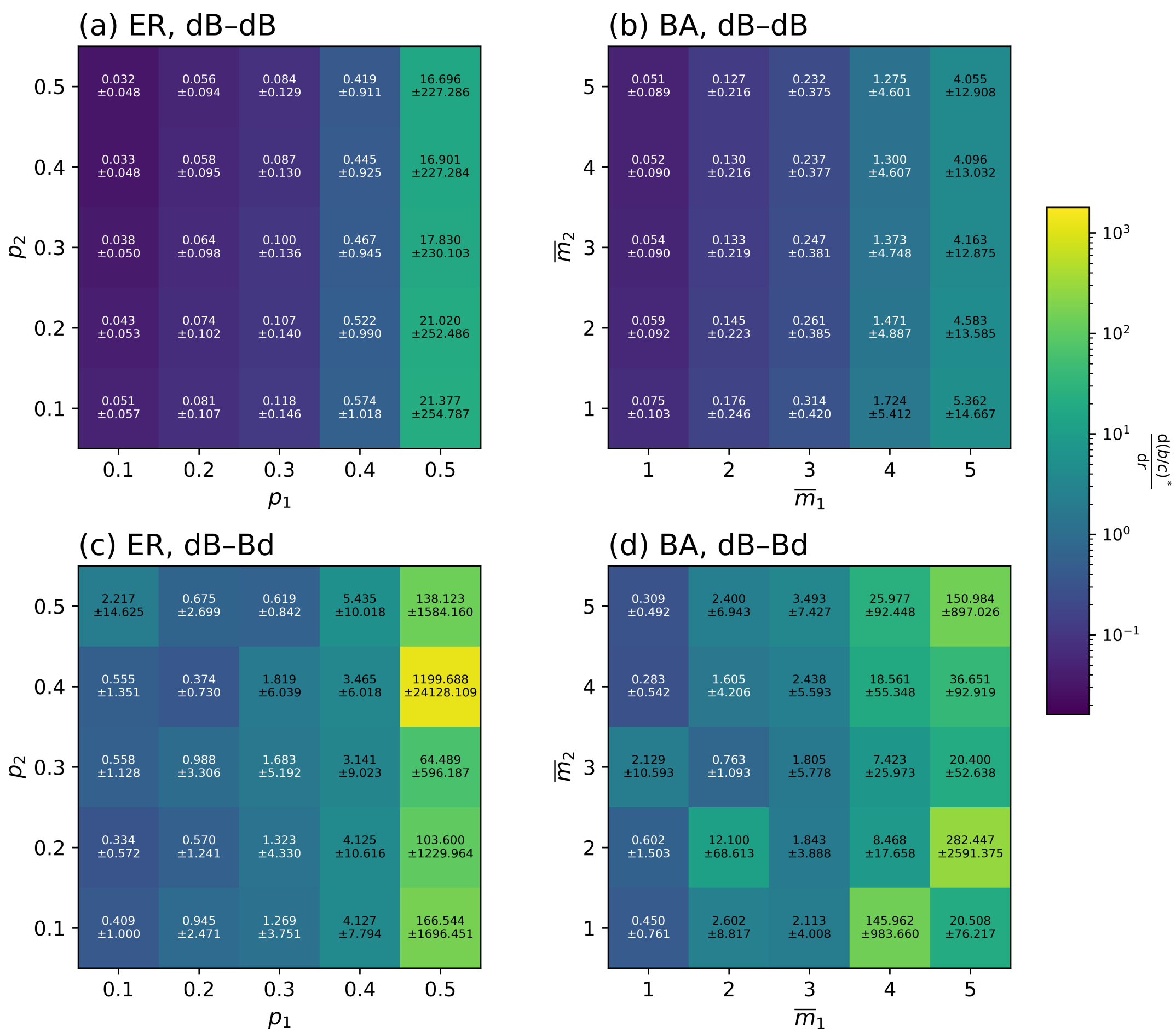

Fig F. Mean, μ, and standard deviation, σ, of $|\mathrm{d}(b/c)^*/\mathrm{d}r|$ for two-layer ER and BA networks with $N = 15$ individuals. (a) ER, dB-dB rule. (b) BA, dB-dB rule. (c) ER, dB-Bd rule. (d) BA, dB-Bd rule. For each (p_1, p_2) or $(\overline{m}_1, \overline{m}_2)$, we computed μ and σ on the basis of all the pairs of two-layer network and initial condition for which cooperation can be favored (i.e., $(b/c)^* > 0$ for the layer-1 network).

Text L Numerical simulations of the stochastic evolutionary dynamics

To validate our theoretical prediction, we carry out direct numerical simulations of the evolutionary dynamics in two-layer networks. For each combination of two-layer network, b/c, r, updating rule, and strength of selection, we use a particular initial condition, run simulations, and estimate the fixation probability for cooperation as the fraction of runs in which cooperation has fixated.

We compare the fixation probability between the theory and direct numerical simulations in Fig G for the two-layer ring network with $N = 10$ individuals used in Fig 4A in the main text and a two-layer BA network with $N = 15$ individuals. We set $r = 2.5$ and $r = 5$ for the ring and BA networks, respectively. We find that the numerically estimated fixation probability closely agrees with the theoretical prediction across b/c values for selection strength $\delta = 0.02$. When $\delta = 0.2$, we observe considerable discrepancies between the theoretical and numerical results, which is expected. For both δ values, coupling with the constant-selection network layer promotes cooperation relative to the one-layer network.

In the main text, we have mainly confined ourselves to small networks up to $N = 15$ individuals. Therefore, we have also examined the fixation probability for two-layer ER and BA networks with $N = 100$ individuals. We first confirmed that the theoretical results for the fixation probability for cooperators and mutants show a typical pattern that small networks show, for both dB-dB and dB-Bd updating rules (Fig H). In particular, $(b/c)^*$ is smaller (i.e., prosociality is promoted) with two coupled network layers if $r > 1$ for both networks and both updating rules. We then compared the fixation probability obtained by the weak selection theory and direct numerical simulations. The results, shown in Fig I, are qualitatively the same as those for smaller networks whose results are shown in Fig G.

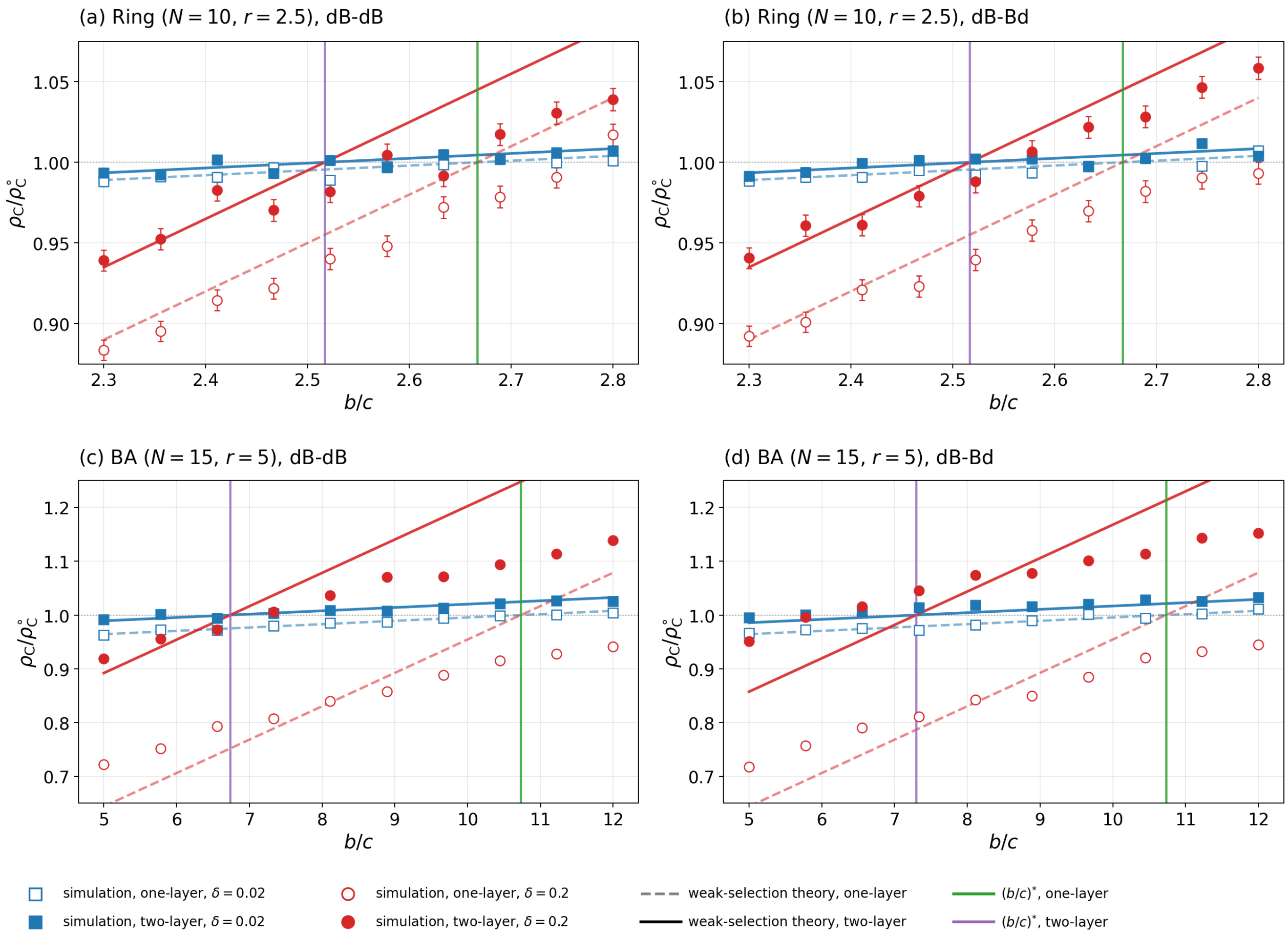

Fig G. Fixation probability for cooperation compared between the weak selection theory and direct numerical simulations on the ring network with $N = 10$ and a BA network with $N = 15$. (a) Ring, $N = 10$, $r = 2.5$, dB-dB. (b) Ring, $N = 10$, $r = 2.5$, dB-Bd. (c) BA, $N = 15$, $r = 5$, dB-dB. (d) BA, $N = 15$, $r = 5$, dB-Bd. The vertical lines represent the $(b/c)^*$ value from our weak selection theory. We obtained the theoretical lines for $\delta \in \{0.02, 0.2\}$ by substituting the δ value in Eq. (3) in the main text. The two-layer ring network is the same as the one used in Fig 4A in the main text. The results for the one-layer networks are the fixation probability for layer 1 separately considered. We set $\overline{m}_1 = \overline{m}_2 = 2$ for the BA network. We use a single instance of two-layer BA network with a fixed initial condition, which is shared between (c) and (d); this is also the case for to the two-layer ER and BA networks used in Figs H and I. We used one end node in the initial three-node chain in layer 1 as the location of the initial cooperator in the BA network. In all networks including the BA network, the initial cooperator in layer 1 and the initial mutant in layer 2 are the same individual. We use the same initial conditions in Figs H and I. On the vertical axis, ρ_C is the fixation probability for a single cooperator; ρ_C° is the reproductive value of the node (denoted by v) on which the single cooperator is initially located (i.e., fixation probability of a neutral mutant placed on v). Because we are considering undirected networks, ρ_C° is equal to the degree of v divided by the sum of the degree over all the nodes [10, 13]. To numerically compute the fixation probability, we ran 10^6 and 5×10^5 simulations for the ring and BA networks, respectively, at each b/c value for $\delta = 0.02$. For $\delta = 0.2$, we ran 2×10^5 and 10^5 simulations for the ring and BA networks, respectively. The number of runs is larger for $\delta = 0.02$ because a weaker selection strength necessitates more runs for accurate estimation of the fixation probability.

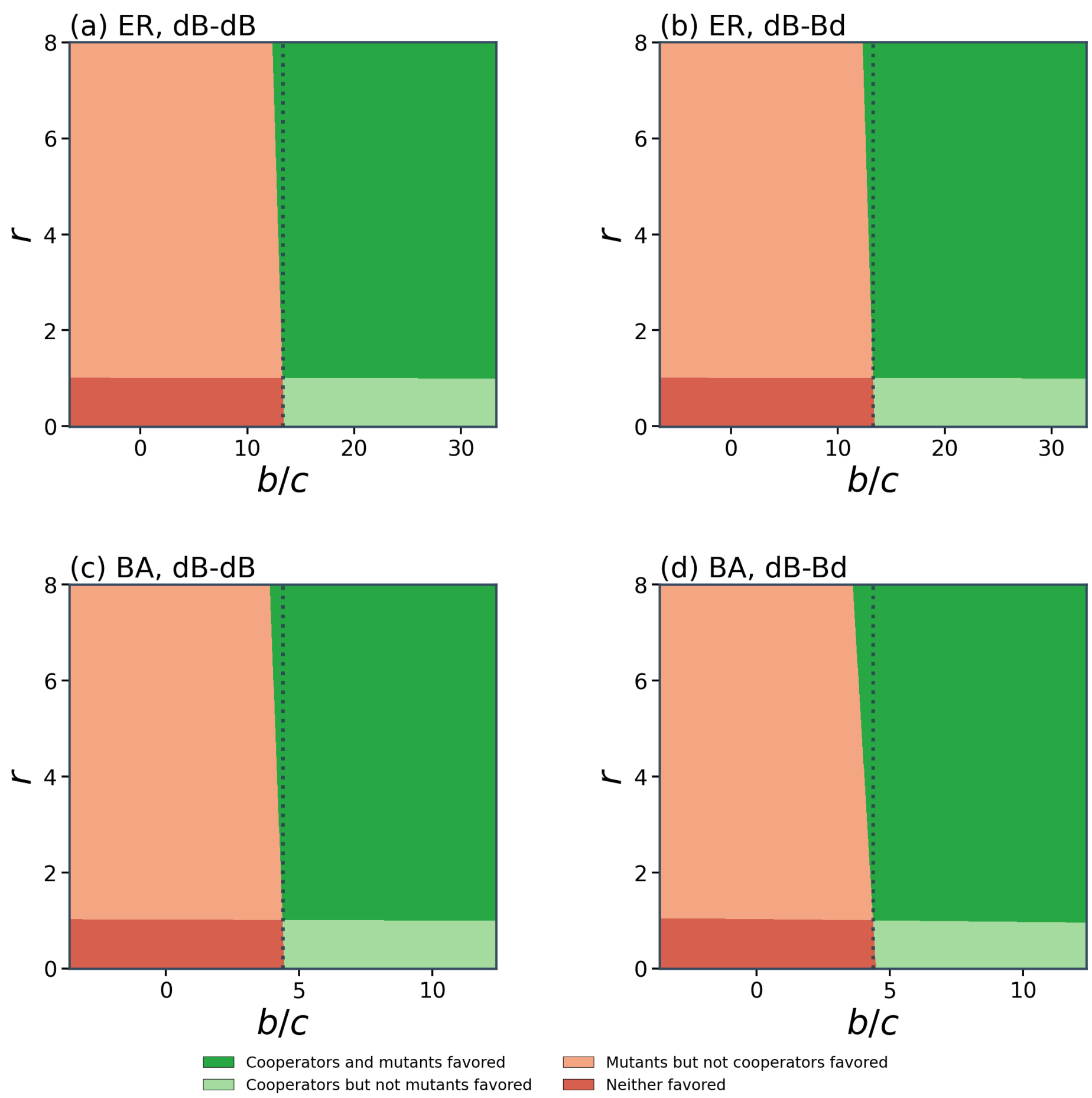

Fig H. Parameter regions in which the cooperator or mutant is selected in two-layer networks with $N = 100$ individuals. The vertical lines represent the $(b/c)^*$ value for the uncoupled layer-1 network. (a) ER, dB-dB. (b) ER, dB-Bd. (c) BA, dB-dB. (d) BA, dB-Bd. We set $p_1 = p_2 = 0.1$ for the ER network and $\overline{m}_1 = \overline{m}_2 = 2$ for the BA network.

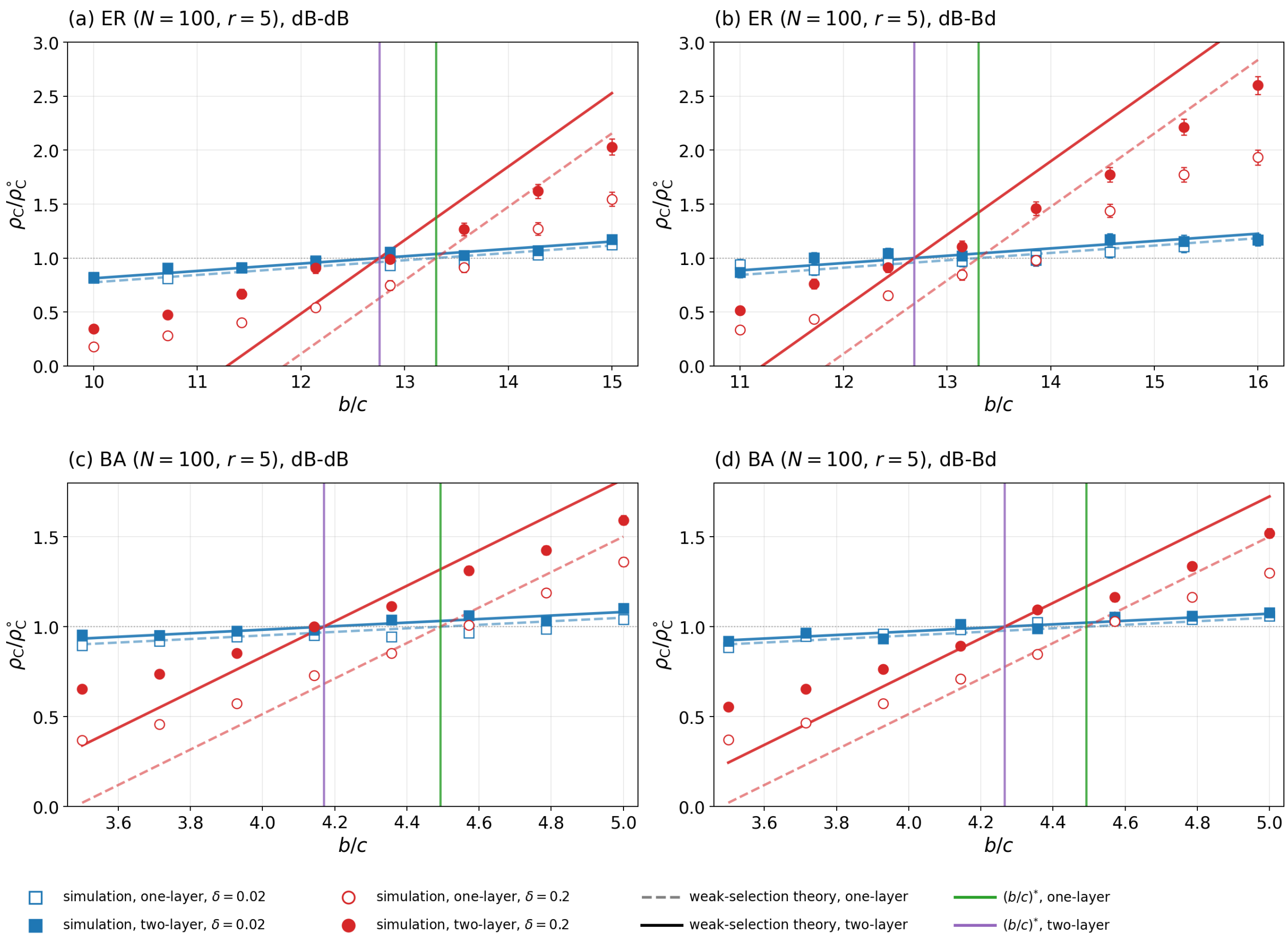

Fig I. Fixation probability for cooperation compared between the weak selection theory and direct numerical simulations on networks with $N = 100$. (a) ER, $N = 100$, dB-dB. (b) ER, $N = 100$, dB-Bd. (c) BA, $N = 100$, dB-dB. (d) BA, $N = 100$, dB-Bd. We set $r = 5$. See the caption of Fig G for legends. To numerically compute the fixation probability, we ran 2×10^5 and 3×10^5 simulations for the ER and BA networks, respectively, at each b/c value for $\delta = 0.02$ under the dB-dB rule. These cases yielded noisier evolutionary dynamics such that we used a larger number of runs than in the other cases. In all other cases, we ran 5×10^4 simulations.

Text M "Fixed benefits, fixed costs" goods scheme

We analyze the case of the ff goods scheme [9]. We first derive the conditions under which the cooperator and mutant are favored under the ff goods scheme. Then, we numerically investigate the conditions for the two-layer networks used in the main text.

M.1 Computation of $(b/c)^*$ and r^*

We recall that, under the pf goods analyzed in the main text, the payoff of the ith individual from layer 1 is given by

$$u_i^{[1]} = -cx_i^{[1]} + \sum_{j=1}^{N} b \frac{w_{ij}^{[1]}}{s_i^{[1]}} x_j^{[1]}. \tag{S135}$$

Under the ff goods, it changes to

$$u_i^{[1]} = -cx_i^{[1]} + \sum_{j=1}^{N} b \frac{w_{ij}^{[1]}}{s_j^{[1]}} x_j^{[1]}. \tag{S136}$$

The payoff from layer 2, i.e., $u_i^{[2]} = x_i^{[2]}(r-1)+1$, remains unchanged.

We rewrite Eq. (S136) as

$$\begin{aligned} u_k^{[1]}(\boldsymbol{x}) &= \sum_{\ell=1}^{N} \left(-c \cdot p_{k\ell}^{[1]} \cdot x_k^{[1]} + b \cdot \frac{w_{k\ell}^{[1]}}{s_\ell^{[1]}} \cdot x_\ell^{[1]} \right) \\ &= \sum_{\ell=1}^{N} \left(-c \cdot p_{k\ell} \cdot x_k^{[1]} + b \cdot \frac{w_{\ell k}^{[1]}}{s_\ell^{[1]}} \cdot x_\ell^{[1]} \right) \\ &= \sum_{\ell=1}^{N} \left(-c \cdot p_{k\ell}^{[1]} \cdot x_k^{[1]} + b \cdot p_{\ell k}^{[1]} \cdot x_\ell^{[1]} \right) \\ &= \sum_{\ell=1}^{N} \left(-C_{k\ell} \cdot x_k^{[1]} + B_{k\ell} \cdot x_\ell^{[1]} \right). \end{aligned} \tag{S137}$$

Then, Eq. (S36) becomes

$$\begin{aligned} \frac{\mathrm{d}}{\mathrm{d}\delta} \rho_A^{[1]}(\boldsymbol{\xi}) \bigg|_{\delta=0} = \sum_{i,j,k=1}^{N} \pi_i^{[1]} m_{k;ji}^{[1]} \Bigg\{ &- \left[\sum_{\ell=1}^{N} \left(\beta_{jk}^{\boldsymbol{\xi}[1]} - \beta_{ik}^{\boldsymbol{\xi}[1]} \right) C_{k\ell} - \left(\beta_{jl}^{\boldsymbol{\xi}[1]} - \beta_{il}^{\boldsymbol{\xi}[1]} \right) B_{k\ell} \right] \\ &+ \left[\left(\gamma_{jk}^{\boldsymbol{\xi}[1,2]} - \gamma_{ik}^{\boldsymbol{\xi}[1,2]} \right)(r-1) + \left(\eta_j^{\boldsymbol{\xi}[1]} - \eta_i^{\boldsymbol{\xi}[1]} \right) \right] \Bigg\}. \end{aligned} \tag{S138}$$

Note that the only difference between Eqs. (S36) and (S138) is the use of $B_{k\ell}$ instead of $B_{\ell k}$. Therefore, the condition for the cooperator to be selected in layer 1, i.e.,

$$\frac{\mathrm{d}}{\mathrm{d}\delta} \rho_A^{[1]}(\boldsymbol{\xi}) \bigg|_{\delta=0} > 0, \tag{S139}$$

is given by

$$\begin{aligned} \sum_{i,j,k=1}^{N} \pi_i^{[1]} m_{k;ji}^{[1]} \Bigg\{ &- \left[\sum_{\ell=1}^{N} \left(\beta_{jk}^{\boldsymbol{\xi}[1]} - \beta_{ik}^{\boldsymbol{\xi}[1]} \right) C_{k\ell} - \left(\beta_{jl}^{\boldsymbol{\xi}[1]} - \beta_{il}^{\boldsymbol{\xi}[1]} \right) B_{k\ell} \right] \\ &+ \left[\left(\gamma_{jk}^{\boldsymbol{\xi}[1,2]} - \gamma_{ik}^{\boldsymbol{\xi}[1,2]} \right)(r-1) + \left(\eta_j^{\boldsymbol{\xi}[1]} - \eta_i^{\boldsymbol{\xi}[1]} \right) \right] \Bigg\} > 0. \end{aligned} \tag{S140}$$

By substituting Eq. (S42) in Eq. (S140), we obtain

$$\sum_{i=1}^{N}\pi_i^{[1]}\sum_{\ell=1}^{N}\left[-\beta_{ii}^{\boldsymbol{\xi}[1]}C_{i\ell}+\beta_{i\ell}^{\boldsymbol{\xi}[1]}B_{i\ell}+\gamma_{ii}^{\boldsymbol{\xi}[1,2]}(r-1)+\eta_i^{\boldsymbol{\xi}[1]}\right]>$$
$$\sum_{i,j=1}^{N}\pi_i^{[1]}\left(p^{[1]}\right)_{ij}^{(2)}\sum_{\ell=1}^{N}\left[-\beta_{ij}^{\boldsymbol{\xi}[1]}C_{jl}+\beta_{il}^{\boldsymbol{\xi}[1]}B_{j\ell}+\gamma_{ij}^{\boldsymbol{\xi}[1,2]}(r-1)+\eta_i^{\boldsymbol{\xi}[1]}\right]. \tag{S141}$$

By substituting $C_{kl}=cp_{kl}^{[1]}$ and $B_{lk}=bp_{lk}^{[1]}$ in Eq. (S141), we obtain

$$\sum_{i=1}^{N}\pi_i^{[1]}\sum_{\ell=1}^{N}\left[-\beta_{ii}^{\boldsymbol{\xi}[1]}cp_{i\ell}^{[1]}+\beta_{i\ell}^{\boldsymbol{\xi}[1]}bp_{\ell i}^{[1]}+\gamma_{ii}^{\boldsymbol{\xi}[1,2]}(r-1)+\eta_i^{\boldsymbol{\xi}[1]}\right]>$$
$$\sum_{i,j=1}^{N}\pi_i^{[1]}\left(p^{[1]}\right)_{ij}^{(2)}\sum_{\ell=1}^{N}\left[-\beta_{ij}^{\boldsymbol{\xi}[1]}cp_{j\ell}^{[1]}+\beta_{i\ell}^{\boldsymbol{\xi}[1]}bp_{\ell j}^{[1]}+\gamma_{ij}^{\boldsymbol{\xi}[1,2]}(r-1)+\eta_i^{\boldsymbol{\xi}[1]}\right]. \tag{S142}$$

By substituting Eqs. (S47) and (S48) in Eq. (S142), we obtain

$$b\left\{\sum_{i=1}^{N}\pi_i^{[1]}\sum_{\ell=1}^{N}\beta_{i\ell}^{\boldsymbol{\xi}[1]}p_{\ell i}^{[1]}-\sum_{i,j=1}^{N}\pi_i^{[1]}\left(p^{[1]}\right)_{ij}^{(2)}\sum_{\ell=1}^{N}\beta_{i\ell}^{\boldsymbol{\xi}[1]}p_{\ell j}^{[1]}\right\}+c\theta_2^{\boldsymbol{\xi}[1]}-(r-1)\,\phi_{2,0}^{\boldsymbol{\xi}[1,2]}>0. \tag{S143}$$

Equation (S143) holds true for the game layer (i.e., layer 1) under the dB rule no matter whether the constant-selection layer (i.e., layer 2) obeys the dB or Bd rule. Under the dB-dB rule, the recurrence equations for computing $\beta_{ii}^{\boldsymbol{\xi}[1]},\beta_{ij}^{\boldsymbol{\xi}[1]}$ (with $i\neq j$), $\gamma_{ij}^{\boldsymbol{\xi}[1,2]}$, and $\eta_i^{\boldsymbol{\xi}[1]}$ are Eqs. (S52), (S54), (S56), and (S57), respectively. Under the dB-Bd rule, the recurrence equations are Eqs. (S52), (S54), (S72), and (S57).

The condition under which the mutant is selected in layer 2 (i.e., constant-selection layer), i.e.,

$$\left.\frac{\mathrm{d}}{\mathrm{d}\delta}\rho_A^{[2]}(\boldsymbol{\xi})\right|_{\delta=0}>0, \tag{S144}$$

is given by

$$\sum_{i,j,k=1}^{N}\pi_i^{[2]}m_{k;ji}^{[2]}\left\{-\left[\sum_{\ell=1}^{N}\left(\gamma_{jk}^{\boldsymbol{\xi}[2,1]}-\gamma_{ik}^{\boldsymbol{\xi}[2,1]}\right)C_{k\ell}-\left(\gamma_{jl}^{\boldsymbol{\xi}[2,1]}-\gamma_{il}^{\boldsymbol{\xi}[2,1]}\right)B_{k\ell}\right]\right.$$
$$\left.+\left[\left(\beta_{jk}^{\boldsymbol{\xi}[2]}-\beta_{ik}^{\boldsymbol{\xi}[2]}\right)(r-1)+\left(\eta_j^{\boldsymbol{\xi}[1]}-\eta_i^{\boldsymbol{\xi}[1]}\right)\right]\right\}>0. \tag{S145}$$

For the dB-dB updating rule, by substituting Eqs. (S47) and (S48) in Eq. (S145), we obtain

$$\sum_{i=1}^{N}\pi_i^{[2]}\sum_{\ell=1}^{N}\left[-\gamma_{ii}^{\boldsymbol{\xi}[2,1]}cp_{i\ell}^{[1]}+\gamma_{i\ell}^{\boldsymbol{\xi}[2,1]}bp_{\ell i}^{[1]}+\beta_{ii}^{\boldsymbol{\xi}[2]}(r-1)+\eta_i^{\boldsymbol{\xi}[2]}\right]>$$
$$\sum_{i,j=1}^{N}\pi_i^{[2]}\left(p^{[2]}\right)_{ij}^{(2)}\sum_{\ell=1}^{N}\left[-\gamma_{ij}^{\boldsymbol{\xi}[2,1]}cp_{j\ell}^{[1]}+\gamma_{i\ell}^{\boldsymbol{\xi}[2,1]}bp_{\ell j}^{[1]}+\beta_{ij}^{\boldsymbol{\xi}[2]}(r-1)+\eta_i^{\boldsymbol{\xi}[2]}\right]. \tag{S146}$$

For the dB-Bd updating rule, we similarly obtain

$$\sum_{i,j=1}^{N}\pi_i^{[2]}p_{ji}^{[2]}\sum_{\ell=1}^{N}\left[-\gamma_{ii}^{\boldsymbol{\xi}[2,1]}cp_{i\ell}^{[1]}+\gamma_{i\ell}^{\boldsymbol{\xi}[2,1]}bp_{i\ell}^{[1]}+\beta_{ii}^{\boldsymbol{\xi}[2]}(r-1)+\eta_i^{\boldsymbol{\xi}[2]}\right]>$$
$$\sum_{i,j=1}^{N}\pi_i^{[2]}p_{ji}^{[2]}\sum_{\ell=1}^{N}\left[-\gamma_{ij}^{\boldsymbol{\xi}[2,1]}cp_{j\ell}^{[1]}+\gamma_{i\ell}^{\boldsymbol{\xi}[2,1]}bp_{j\ell}^{[1]}+\beta_{ij}^{\boldsymbol{\xi}[2]}(r-1)+\eta_i^{\boldsymbol{\xi}[2]}\right]. \tag{S147}$$

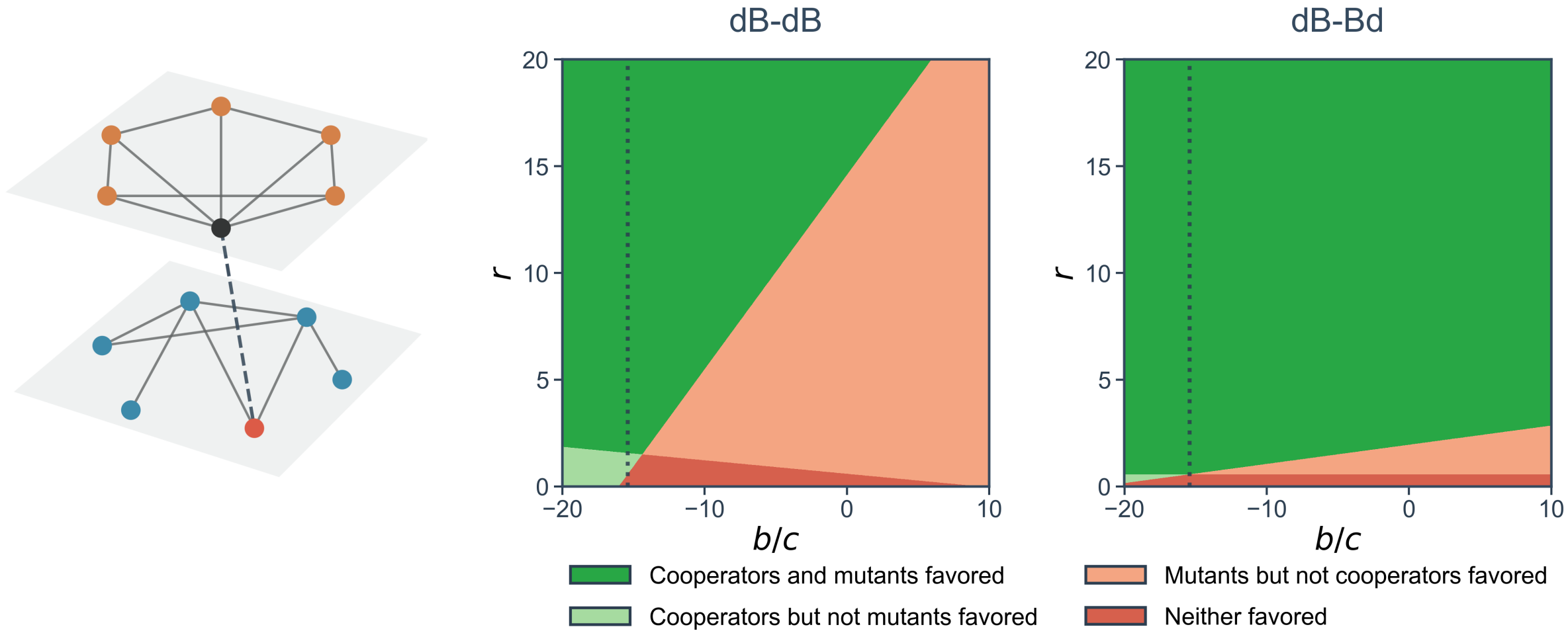

Fig J. Parameter regions in which the cooperator or mutant is favored under the ff good scheme for the two-layer network used in Fig 4B in the main text (replicated in the left panel of this figure). The vertical dotted lines indicate the $(b/c)^*$ value $(= -15.41)$ when the two layers are uncoupled.

Under the dB-dB rule, the recurrence equations for computing $\beta_{ii}^{\boldsymbol{\xi}[1]}$, $\beta_{ij}^{\boldsymbol{\xi}[1]}$ (with $i \neq j$), $\gamma_{ij}^{\boldsymbol{\xi}[1,2]}$, and $\eta_i^{\boldsymbol{\xi}[1]}$ are Eqs. (S52), (S54), (S56), and (S57), respectively. Under the dB-Bd rule, the recurrence equations are Eqs. (S70), (S70), (S72), and (S57).

M.2 Numerical results

In Fig J, we show the $(b/c)^*$ and r^* values for the two-layer network used in Fig 4B in the main text, but under the ff goods scheme. For the other three networks used in Fig 4, the $(b/c)^*$ value is the same between the ff and pf goods because the game layer (i.e., layer 1) in these two-layer networks are regular graphs; Eqs. (S135) and (S136) are identical for regular graphs. For the network shown in Fig J, we obtain qualitatively the same behavior of $(b/c)^*$ as a function of r between the two goods schemes; compare Fig J(b) and Fig 4B. The r^* value as a function of b/c is also similar between the ff and pf goods.

M.3 Random graphs

For the ff goods scheme, we analyzed the condition under which the cooperator and mutant are selected in two-layer ER and BA networks on 15 individuals.

We show in Fig K(a) and (b) the results for the two-layer ER and BA networks, respectively, under the dB-dB updating rule. We find that all the layer-1 networks enable cooperation, not spite (see the upper part of Fig K(a) and (b)). The responsiveness of $(b/c)^*$ as the constant selection changes, quantified by $|\mathrm{d}(b/c)^*/\mathrm{d}r|$, increases as the edge density in layer 1 increases or that in layer 2 decreases (see the lower part of Fig K(a) and (b)). These results are similar to those for the pf goods shown in Fig 5A and 5B in the main text. The results are qualitatively the same for the dB-Bd rule, with overall larger $|\mathrm{d}(b/c)^*/\mathrm{d}r|$ than the case of the dB-dB rule (see Fig K(c) and (d)). Finally, we have verified that the results do not substantially change when we switch the measurement of $|\mathrm{d}(b/c)^*/\mathrm{d}r|$ from the median and percentiles to the mean and standard deviation (see Fig L).

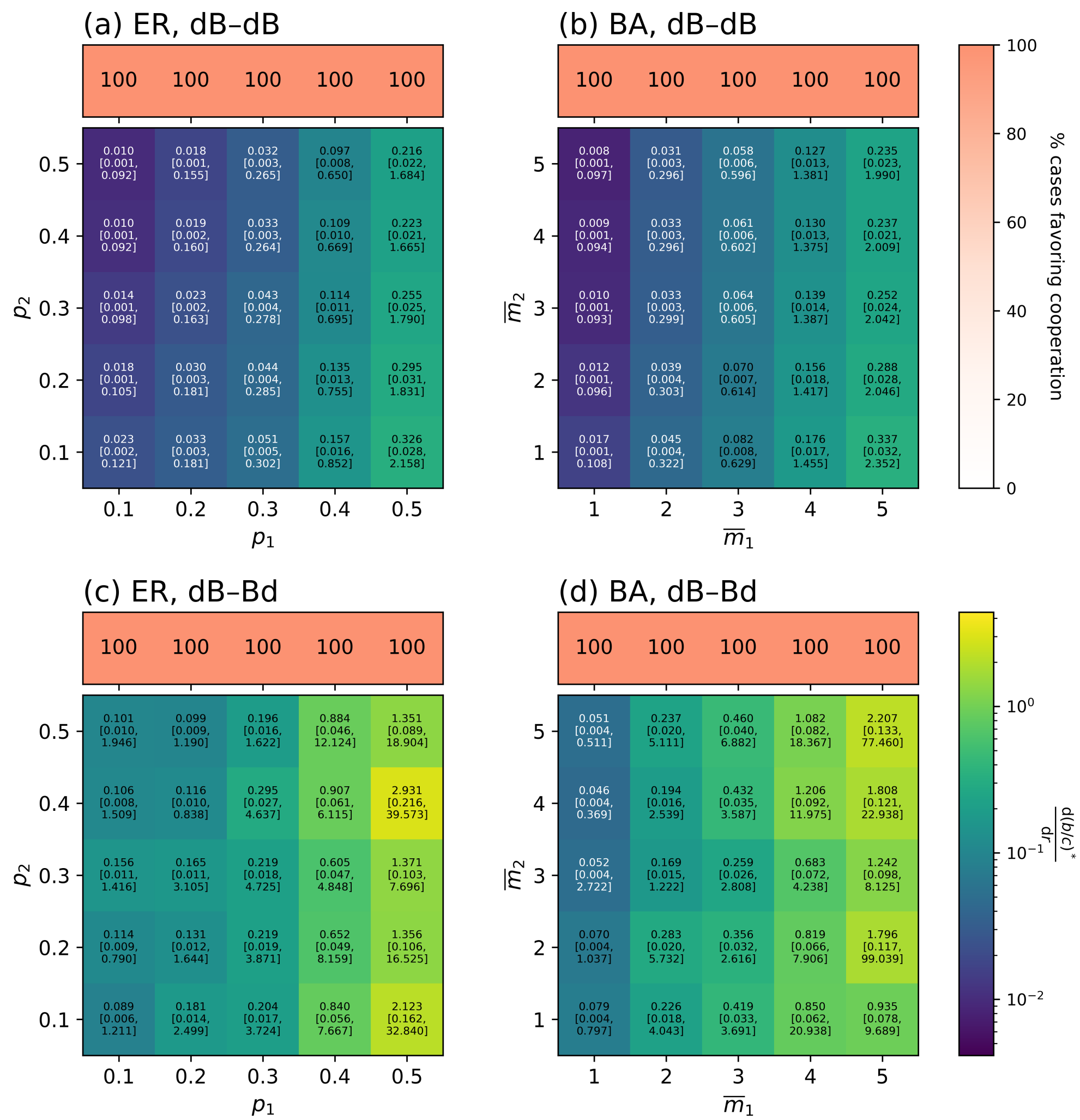

Fig K. Evolution of cooperation in two-layer ER and BA networks under the ff goods scheme. (a) ER, dB-dB rule. (b) BA, dB-dB rule. (c) ER, dB-Bd rule. (d) BA, dB-Bd rule. The upper part of each panel shows the fraction of pairs of single-layer network and initial condition that yield cooperation when $b/c > (b/c)^*$ for a threshold value $(b/c)^* > 0$. The fraction values are the same between (a) and (c) and between (b) and (d) because whether or not $(b/c)^* > 0$ in layer 1 does not depend on the updating rule used in layer 2. The lower part of each panel shows the median along with the 5th and 95th percentiles (in square brackets) of $|\mathrm{d}(b/c)^*/\mathrm{d}r|$ for pairs of two-layer network and initial condition. Each two-layer network is composed of $N = 15$ individuals.

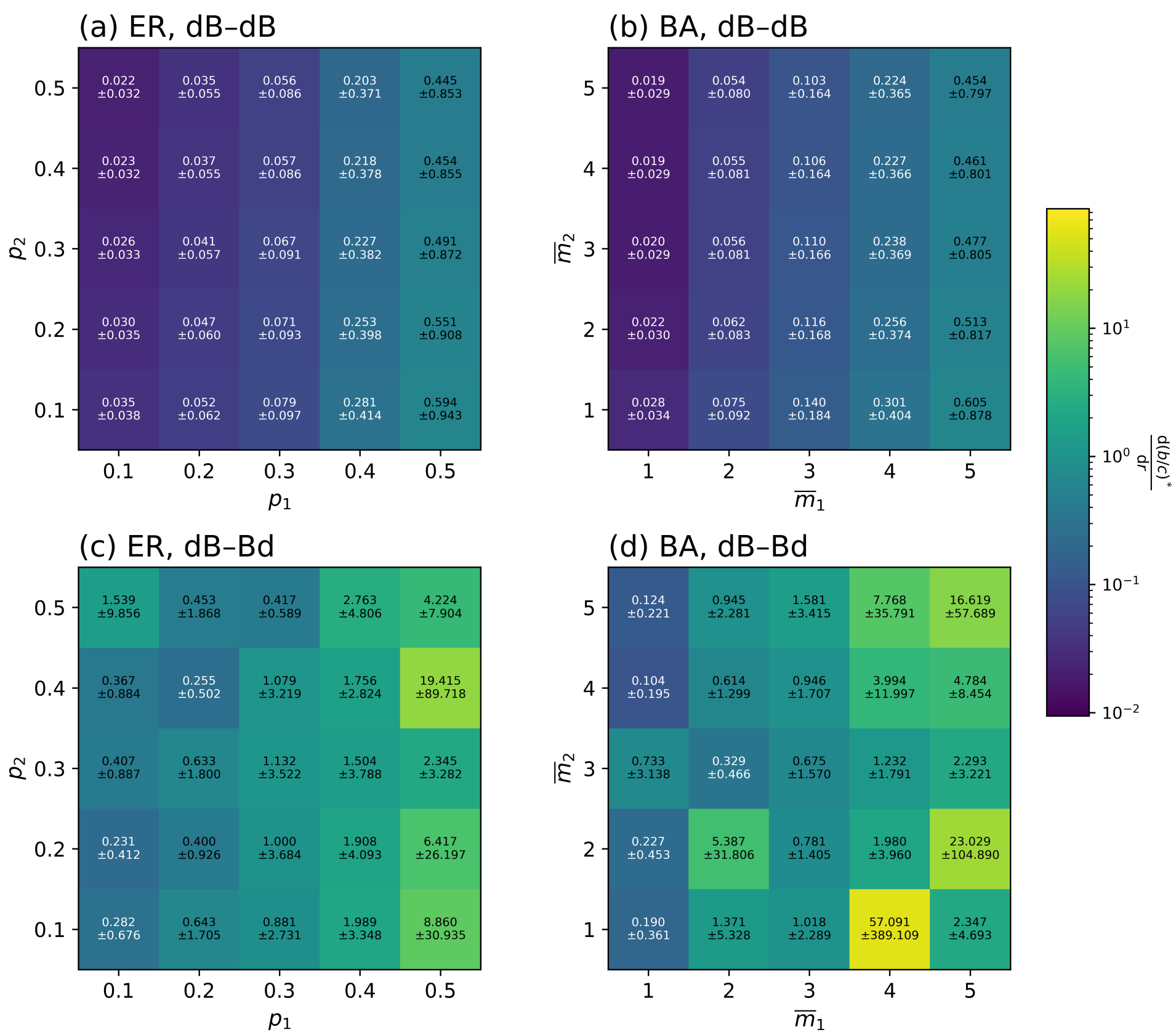

Fig L. Mean, μ, and standard deviation, σ, of $|\mathrm{d}(b/c)^*/\mathrm{d}r|$ for two-layer ER and BA networks with $N = 15$ individuals under the ff goods scheme. (a) ER, dB-dB rule. (b) BA, dB-dB rule. (c) ER, dB-Bd rule. (d) BA, dB-Bd rule. See the caption of Fig F for details of the computation.

M.4 Empirical networks

We show in Fig M the results for the VC7 and LLF law two-layer networks under the ff goods scheme. The results are qualitatively the same as those under the pf goods scheme. It should be noted that the quantitative difference between the ff and pf goods schemes is large in this case; for the VC7 network, we have obtained $(b/c)^* = -298.7$, which is far from $(b/c)^* = -89.5$ in the case of the pf goods shown in the main text; for the LLF law network, we have obtained $(b/c)^* = 28.66$ under the ff goods scheme, whereas the main text shows $(b/c)^* = 52.4$ under the pf goods scheme.

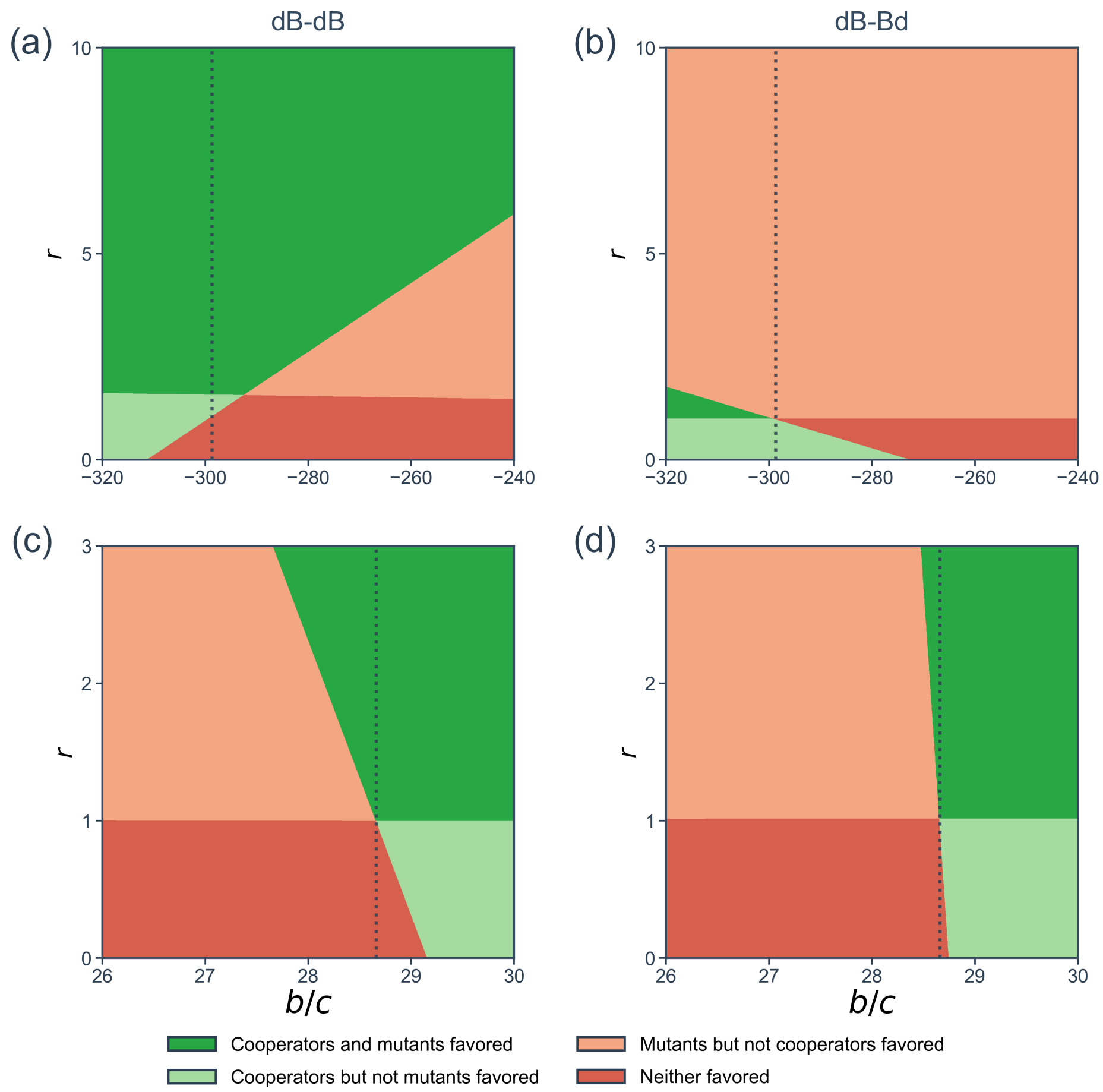

Fig M. Parameter regions in which the cooperator or mutant is selected in empirical two-layer networks under the ff goods scheme. The vertical lines represent the $(b/c)^*$ value for the uncoupled layer-1 network. (a) VC7, dB-dB. (b) VC7, dB-Bd. (c) LLF, dB-dB. (d) LLF, dB-Bd. The single-layer VC7 and LLF networks yield $(b/c)^* = -298.7$ and $(b/c)^* = 28.66$, respectively.

Text N Fixation time

We computed the fixation time for cooperation under the dB-dB rule combined with the pf good scheme. We used the four synthetic two-layer networks used in Fig 4 and the VC7 network. We did not use the LLF network because numerical estimation of the fixation time of cooperation when starting from a single cooperator is computationally demanding. We generated 1,600,000 runs that finished with fixation of cooperation in layer 1 no matter whether the resident or mutant type fixated in layer 2 for the four synthetic networks. We reduced the number of runs to 800,000 for the VC7 network. Then, we recorded the time to fixation of cooperation and averaged it over all the runs. It should be noted that we defined the fixation time as the time at which the cooperator has fixated regardless of whether or not fixation has been attained in layer 2. This choice is to enable fair comparison between the two-layer and one-layer networks; if we wait for the fixation of both layers, then the fixation time would be obviously shorter for one-layer networks than the two-layer networks whose layer-1 network is the same as the one-layer network under comparison. As reference, we also computed the mean fixation time for the one-layer network that is layer 1 of each two-layer network analyzed.

We show the mean fixation time for cooperation in Fig N. We find that the fixation time for the four synthetic two-layer networks is not much larger than that for the corresponding one-layer networks across the parameter values and the choice of two-layer network. For the VC7 network, the two-layer network needs about 1.6–2.8 more time to fixation than one-layer counterparts.

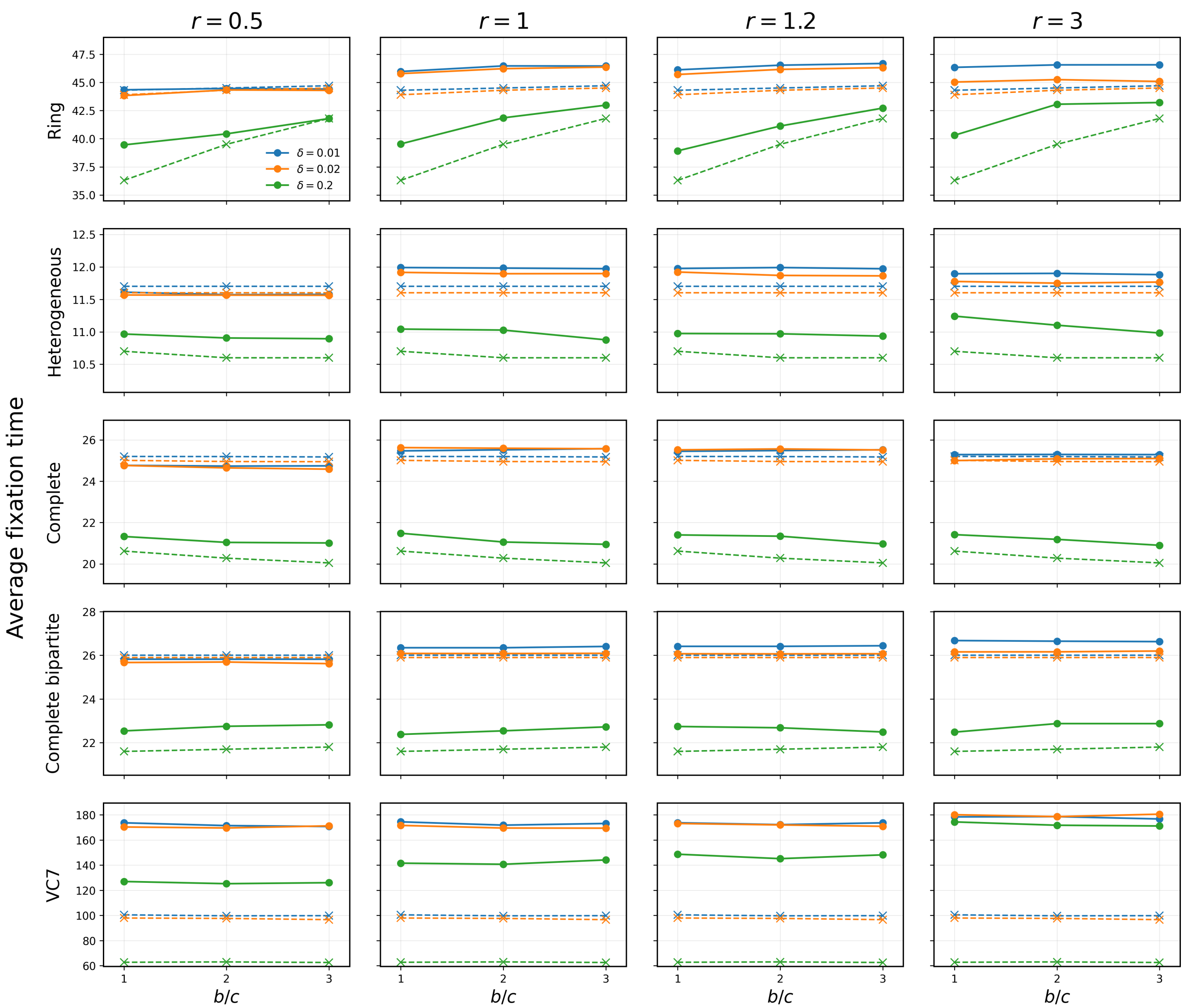

Fig N. Mean fixation time for cooperation in the four synthetic networks and the VC7 network. The ring, heterogeneous, complete, and complete bipartite networks refer to the two-layer networks used in Fig 4A, (b), (c), and (d), respectively. The solid and dashed lines represent the mean fixation time of cooperation in layer 1 of the two-layer networks and the single-layer counterparts, respectively. We used $r \in \{0.5, 1, 1.2, 3\}$.

Supplementary References

[1] Q. Su, A. McAvoy, Y. Mori, and J. B. Plotkin, Evolution of prosocial behaviours in multilayer populations, *Nature Human Behaviour*, 6, 338–348, 2022.

[2] B. Allen and A. McAvoy, A mathematical formalism for natural selection with arbitrary spatial and genetic structure, *Journal of Mathematical Biology*, 78, 1147–1210, 2019.

[3] A. McAvoy and B. Allen, Fixation probabilities in evolutionary dynamics under weak selection, *Journal of Mathematical Biology*, 82, 14, 2021.

[4] R. A. Fisher, *The Genetical Theory of Natural Selection: A Complete Variorum Edition*. Oxford University Press, Oxford, UK, 1999.

[5] P. D. Taylor, Allele-frequency change in a class-structured population, *American Naturalist*, 135, 95–106, 1990.

[6] P. D. Taylor, Inclusive fitness arguments in genetic models of behaviour, *Journal of Mathematical Biology*, 34, 654–674, 1996.

[7] A. Grafen, A theory of Fisher's reproductive value, *Journal of Mathematical Biology*, 53, 15–60, 2006.

[8] W. Maciejewski, Reproductive value in graph-structured populations, *Journal of Theoretical Biology*, 340, 285–293, 2014.

[9] A. McAvoy, B. Allen, and M. A. Nowak, Social goods dilemmas in heterogeneous societies, *Nature Human Behaviour*, 4, 819–831, 2020.

[10] V. Sood, T. Antal, and S. Redner, Voter models on heterogeneous networks, *Physical Review E*, 77, 041121, 2008.

[11] N. Masuda and H. Ohtsuki, Evolutionary dynamics and fixation probabilities in directed networks, *New Journal of Physics*, 11, 033012, 2009.

[12] H. Ohtsuki, C. Hauert, E. Lieberman, and M. A. Nowak, A simple rule for the evolution of cooperation on graphs and social networks, *Nature*, 441, 502–505, 2006.

[13] B. Allen, G. Lippner, Y.-T. Chen, B. Fotouhi, N. Momeni, S.-T. Yau, and M. A. Nowak, Evolutionary dynamics on any population structure, *Nature*, 544, 227–230, 2017.

[14] M. A. Nowak and R. M. May, Evolutionary games and spatial chaos, *Nature*, 359, 826–829, 1992.

[15] M. A. Nowak and R. M. May, The spatial dilemmas of evolution, *International Journal of Bifurcation and Chaos*, 3, 35–78, 1993.

[16] M. A. Nowak, *Evolutionary Dynamics*. Belknap Press of Harvard University Press, Cambridge, MA, 2006.
